# Specific and off-target immune responses following COVID-19 vaccination with ChAdOx1-S and BNT162b2 vaccines—an exploratory sub-study of the BRACE trial

**DOI:** 10.1016/j.ebiom.2024.105100

**Published:** 2024-04-24

**Authors:** Nicole L. Messina, Susie Germano, Rebecca McElroy, Rhian Bonnici, Branka Grubor-Bauk, David J. Lynn, Ellie McDonald, Suellen Nicholson, Kirsten P. Perrett, Laure F. Pittet, Rajeev Rudraraju, Natalie E. Stevens, Kanta Subbarao, Nigel Curtis, BRACE trial, Nigel Curtis, Andrew Davidson, Kaya Gardiner, Amanda Gwee, Tenaya Jamieson, Nicole Messina, Thilanka Morawakage, Susan Perlen, Kirsten Perrett, Laure Pittet, Amber Sastry, Jia Wei Teo, Francesca Orsini, Katherine Lee, Cecilia Moore, Suzanna Vidmar, Laure Pittet, Rashida Ali, Ross Dunn, Peta Edler, Grace Gell, Casey Goodall, Richard Hall, Ann Krastev, Nathan La, Ellie McDonald, Nick McPhate, Thao Nguyen, Jack Ren, Luke Stevens, Nicole Messina, Ahmed Alamrousi, Rhian Bonnici, Thanh Dang, Susie Germano, Jenny Hua, Rebecca McElroy, Monica Razmovska, Scott Reddiex, Xiaofang Wang, Jeremy Anderson, Kristy Azzopardi, Vicki Bennett-Wood, Anna Czajko, Nadia Mazarakis, Conor McCafferty, Frances Oppedisano, Belinda Ortika, Casey Pell, Leena Spry, Ryan Toh, Sunitha Velagapudi, Amanda Vlahos, Ashleigh Wee-Hee, Pedro Ramos, Karina De La Cruz, Dinusha Gamage, Anushka Karunanayake, Isabella Mezzetti, Benjamin Ong, Ronita Singh, Enoshini Sooriyarachchi, Suellen Nicholson, Natalie Cain, Rianne Brizuela, Han Huang, Veronica Abruzzo, Morgan Bealing, Patricia Bimboese, Kirsty Bowes, Emma Burrell, Joyce Chan, Jac Cushnahan, Hannah Elborough, Olivia Elkington, Kieran Fahey, Monique Fernandez, Catherine Flynn, Sarah Fowler, Marie Gentile Andrit, Bojana Gladanac, Catherine Hammond, Norine Ma, Sam Macalister, Emmah Milojevic, Jesutofunmi Mojeed, Jill Nguyen, Liz O’Donnell, Nadia Olivier, Isabelle Ooi, Stephanie Reynolds, Lisa Shen, Barb Sherry, Judith Spotswood, Jamie Wedderburn, Angela Younes, Donna Legge, Jason Bell, Jo Cheah, Annie Cobbledick, Kee Lim, Sonja Elia, Lynne Addlem, Anna Bourke, Clare Brophy, Nadine Henare, Narelle Jenkins, Francesca Machingaifa, Skye Miller, Kirsten Mitchell, Sigrid Pitkin, Kate Wall, Paola Villanueva, Nigel Crawford, Laure Pittet, Wendy Norton, Niki Tan, Thilakavathi Chengodu, Diane Dawson, Victoria Gordon, Tony Korman, Jess O’Bryan, Veronica Abruzzo, Sophie Agius, Samantha Bannister, Jess Bucholc, Alison Burns, Beatriz Camesella, John Carlin, Marianna Ciaverella, Maxwell Curtis, Stephanie Firth, Christina Guo, Matthew Hannan, Erin Hill, Sri Joshi, Katherine Lieschke, Megan Mathers, Sasha Odoi, Ashleigh Rak, Chris Richards, Leah Steve, Carolyn Stewart, Eva Sudbury, Helen Thomson, Emma Watts, Fiona Williams, Angela Young, Penny Glenn, Andrew Kaynes, Amandine Philippart De Floy, Sandy Buchanan, Thijs Sondag, Ivy Xie, Harriet Edmund, Bridie Byrne, Tom Keeble, Belle Ngien, Fran Noonan, Michelle Wearing-Smith, Alison Clarke, Pemma Davies, Oliver Eastwood, Alric Ellinghaus, Rachid Ghieh, Zahra Hilton, Emma Jennings, Athina Kakkos, Iris Liang, Katie Nicol, Sally O’Callaghan, Helen Osman, Gowri Rajaram, Sophia Ratcliffe, Victoria Rayner, Ashleigh Salmon, Angela Scheppokat, Aimee Stevens, Rebekah Street, Nicholas Toogood, Nicholas Wood, Twinkle Bahaduri, Therese Baulman, Jennifer Byrne, Candace Carter, Mary Corbett, Aiken Dao, Maria Desylva, Andrew Dunn, Evangeline Gardiner, Rosemary Joyce, Rama Kandasamy, Craig Munns, Lisa Pelayo, Ketaki Sharma, Katrina Sterling, Caitlin Uren, Clinton Colaco, Mark Douglas, Kate Hamilton, Adam Bartlett, Brendan McMullan, Pamela Palasanthiran, Phoebe Williams, Justin Beardsley, Nikki Bergant, Renier Lagunday, Kristen Overton, Jeffrey Post, Yasmeen Al-Hindawi, Sarah Barney, Anthony Byrne, Lee Mead, Marshall Plit, David Lynn, Saoirse Benson, Stephen Blake, Rochelle Botten, Tee Yee Chern, Georgina Eden, Liddy Griffith, Jane James, Miriam Lynn, Angela Markow, Domenic Sacca, Natalie Stevens, Steve Wesselingh, Catriona Doran, Simone Barry, Alice Sawka, Sue Evans, Louise Goodchild, Christine Heath, Meredith Krieg, Helen Marshall, Mark McMillan, Mary Walker, Peter Richmond, Nelly Amenyogbe, Christina Anthony, Annabelle Arnold, Beth Arrowsmith, Rym Ben-Othman, Sharon Clark, Jemma Dunnill, Nat Eiffler, Krist Ewe, Carolyn Finucane, Lorraine Flynn, Camille Gibson, Lucy Hartnell, Elysia Hollams, Heidi Hutton, Lance Jarvis, Jane Jones, Jan Jones, Karen Jones, Jennifer Kent, Tobias Kollmann, Debbie Lalich, Wenna Lee, Rachel Lim, Sonia McAlister, Fiona McDonald, Andrea Meehan, Asma Minhaj, Lisa Montgomery, Melissa O’Donnell, Jaslyn Ong, Joanne Ong, Kimberley Parkin, Glady Perez, Catherine Power, Shadie Rezazadeh, Holly Richmond, Sally Rogers, Nikki Schultz, Margaret Shave, Patrycja Skut, Lisa Stiglmayer, Alexandra Truelove, Ushma Wadia, Rachael Wallace, Justin Waring, Michelle England, Erin Latkovic, Laurens Manning, Susan Herrmann, Michaela Lucas, Marcus Lacerda, Paulo Henrique Andrade, Fabiane Bianca Barbosa, Dayanne Barros, Larissa Brasil, Ana Greyce Capella, Ramon Castro, Erlane Costa, Dilcimar de Souza, Maianne Dias, José Dias, Klenilson Ferreira, Paula Figueiredo, Thamires Freitas, Ana Carolina Furtado, Larissa Gama, Vanessa Godinho, Cintia Gouy, Daniele Hinojosa, Bruno Jardim, Tyane Jardim, Joel Junior, Augustto Lima, Bernardo Maia, Adriana Marins, Kelry Mazurega, Tercilene Medeiros, Rosangela Melo, Marinete Moraes, Elizandra Nascimento, Juliana Neves, Maria Gabriela Oliveira, Thais Oliveira, Ingrid Oliveira, Arthur Otsuka, Rayssa Paes, Handerson Pereira, Gabrielle Pereira, Christiane Prado, Evelyn Queiroz, Laleyska Rodrigues, Bebeto Rodrigues, Vanderson Sampaio, Anna Gabriela Santos, Daniel Santos, Tilza Santos, Evelyn Santos, Ariandra Sartim, Ana Beatriz Silva, Juliana Silva, Emanuelle Silva, Mariana Simão, Caroline Soares, Antonny Sousa, Alexandre Trindade, Fernando Val, Adria Vasconcelos, Heline Vasconcelos, Julio Croda, Carolinne Abreu, Katya Martinez Almeida, Camila Bitencourt de Andrade, Jhenyfer Thalyta Campos Angelo, Ghislaine Gonçalvez de Araújo Arcanjo, Bianca Maria Silva Menezes Arruda, Wellyngthon Espindola Ayala, Adelita Agripina Refosco Barbosa, Felipe Zampieri Vieira Batista, Fabiani de Morais Batista, Miriam de Jesus Costa, Mariana Garcia Croda, Lais Alves da Cruz, Roberta Carolina Pereira Diogo, Rodrigo Cezar Dutra Escobar, Iara Rodrigues Fernandes, Leticia Ramires Figueiredo, Leandro Galdino Cavalcanti Gonçalves, Sarita Lahdo, Joyce dos Santos Lencina, Guilherme Teodoro de Lima, Larissa Santos Matos, Bruna Tayara Leopoldina Meireles, Debora Quadros Moreira, Lilian Batista Silva Muranaka, Adriely de Oliveira, Karla Regina Warszawski de Oliveira, Matheus Vieira de Oliveira, Roberto Dias de Oliveira, Andrea Antonia Souza de Almeida dos Reis Pereira, Marco Puga, Caroliny Veron Ramos, Thaynara Haynara Souza da Rosa, Karla Lopes dos Santos, Claudinalva Ribeiro dos Santos, Dyenyffer Stéffany Leopoldina dos Santos, Karina Marques Santos, Paulo César Pereira da Silva, Paulo Victor Rocha da Silva, Débora dos Santos Silva, Patricia Vieira da Silva, Bruno Freitas da Rosa Soares, Mariana Gazzoni Sperotto, Mariana Mayumi Tadokoro, Daniel Tsuha, Hugo Miguel Ramos Vieira, Margareth Maria Pretti Dalcolmo, Cíntia Maria Lopes Alves da Paixão, Gabriela Corrêa E Castro, Simone Silva Collopy, Renato da Costa Silva, Samyra Almeida da Silveira, Alda Maria Da-Cruz, Alessandra Maria da Silva Passos de Carvalho, Rita de Cássia Batista, Maria Luciana Silva De Freitas, Aline Gerhardt de Oliveira Ferreira, Ana Paula Conceição de Souza, Paola Cerbino Doblas, Ayla Alcoforado da Silva dos Santos, Vanessa Cristine de Moraes dos Santos, Dayane Alves dos Santos Gomes, Anderson Lage Fortunato, Adriano Gomes-Silva, Monique Pinto Gonçalves, Paulo Leandro Garcia Meireless Junior, Estela Martins da Costa Carvalho, Fernando do Couto Motta, Ligia Maria Olivo de Mendonça, Girlene dos Santos Pandine, Rosa Maria Plácido Pereira, Ivan Ramos Maia, Jorge Luiz da Rocha, João Victor Paiva Romano, Glauce dos Santos, Erica Fernandes da Silva, Marilda Agudo Mendonça Teixeira de Siqueira, Ágatha Cristinne Prudêncio Soares, Marc Bonten, Sandra Franch Arroyo, Henny Ophorst-den Besten, Anna Boon, Karin M. Brakke, Axel Janssen, Marijke A.H. Koopmans, Toos Lemmens, Titia Leurink, Cristina Prat-Aymerich, Engelien Septer-Bijleveld, Kimberly Stadhouders, Darren Troeman, Marije van der Waal, Marjoleine van Opdorp, Nicolette van Sluis, Beatrijs Wolters, Jan Kluytmans, Jannie Romme, Wouter van den Bijllaardt, Linda van Mook, M.M.L (Miranda) van Rijen, Margreet Filius, Jet Gisolf, Frances Greven, Danique Huijbens, Robert Jan Hassing, Roos Pon, Lieke Preijers, Joke van Leusen, Harald Verheij, Wim Boersma, Evelien Brans, Paul Kloeg, Kitty Molenaar-Groot, Nhat Khanh Nguyen, Nienke Paternotte, Anke Rol, Lida Stooper, Helga Dijkstra, Esther Eggenhuizen, Lucas Huijs, Simone Moorlag, Mihai Netea, Eva Pranger, Esther Taks, Jaap ten Oever, Rob ter Heine, Kitty Blauwendraat, Bob Meek, Isil Erkaya, Houda Harbech, Nienke Roescher, Rifka Peeters, Menno te Riele, Carmen Zhou, Esther Calbo, Cristina Badia Marti, Emma Triviño Palomares, Tomás Perez Porcuna, Anabel Barriocanal, Ana Maria Barriocanal, Irma Casas, Jose Dominguez, Maria Esteve, Alicia Lacoma, Irene Latorre, Gemma Molina, Barbara Molina, Antoni Rosell, Sandra Vidal, Lydia Barrera, Natalia Bustos, Ines Portillo Calderón, David Gutierrez Campos, Jose Manuel Carretero, Angel Dominguez Castellano, Renato Compagnone, Encarnacion Ramirez de Arellano, Almudena de la Serna, Maria Dolores del Toro Lopez, Marie-Alix Clement Espindola, Ana Belen Martin Gutierrez, Alvaro Pascual Hernandez, Virginia Palomo Jiménez, Elisa Moreno, Nicolas Navarrete, Teresa Rodriguez Paño, Jesús Rodríguez-Baño, Enriqueta Tristán, Maria Jose Rios Villegas, Atsegiñe Canga Garces, Erika Castro Amo, Raquel Coya Guerrero, Josune Goikoetxea, Leticia Jorge, Cristina Perez, María Carmen Fariñas Álvarez, Manuel Gutierrez Cuadra, Francisco Arnaiz de las Revillas Almajano, Pilar Bohedo Garcia, Teresa Giménez Poderos, Claudia González Rico, Blanca Sanchez, Olga Valero, Noelia Vega, John Campbell, Anna Barnes, Helen Catterick, Tim Cranston, Phoebe Dawe, Emily Fletcher, Liam Fouracre, Alison Gifford, John Kirkwood, Christopher Martin, Amy McAnew, Marcus Mitchell, Georgina Newman, Abby O’Connell, Jakob Onysk, Lynne Quinn, Shelley Rhodes, Samuel Stone, Lorrie Symons, Harry Tripp, Adilia Warris, Darcy Watkins, Bethany Whale, Alex Harding, Gemma Lockhart, Kate Sidaway-Lee, John Campbell, Sam Hilton, Sarah Manton, Daniel Webber-Rookes, Rachel Winder, James Moore, Freya Bateman, Michael Gibbons, Bridget Knight, Julie Moss, Sarah Statton, Josephine Studham, Lydia Hall, Will Moyle, Tamsin Venton

**Affiliations:** a Infectious Diseases Group, Infection, Immunity and Global Health Theme, Murdoch Children’s Research Institute, Parkville, VIC, Australia; b Department of Paediatrics, The University of Melbourne, Parkville, VIC, Australia; c Department of Microbiology and Immunology, University of Melbourne at the Peter Doherty Institute for Infection and Immunity, Melbourne, VIC, Australia; d Paediatric Infectious Diseases Unit, Geneva University Hospitals and Faculty of Medicine, Geneva, Switzerland; e Viral Immunology Group, Adelaide Medical School, University of Adelaide and Basil Hetzel Institute for Translational Health Research, Adelaide, SA, Australia; f Precision Medicine Theme, South Australian Health and Medical Research Institute, Adelaide, SA, Australia; g Flinders Health and Medical Research Institute, Flinders University, Bedford Park, SA, Australia; h Victorian Infectious Diseases Reference Laboratory, The Royal Melbourne Hospital, The Peter Doherty Institute for Infection and Immunity, Melbourne, VIC, Australia; i Population Allergy Group, Murdoch Children’s Research Institute, Parkville, VIC, Australia; j Department of Allergy and Immunology, The Royal Children’s Hospital Melbourne, Parkville, VIC, Australia; k WHO Collaborating Centre for Reference and Research on Influenza, Peter Doherty Institute for Infection and Immunity, Elizabeth Street, Melbourne, VIC, Australia; l Infectious Diseases, The Royal Children’s Hospital Melbourne, Parkville, VIC, Australia

**Keywords:** Vaccine, Off-target, Immunoregulation, Cytokine, SARS-CoV-2, Heterologous immunity

## Abstract

**Background:**

The COVID-19 pandemic led to the rapid development and deployment of several highly effective vaccines against SARS-CoV-2. Recent studies suggest that these vaccines may also have off-target effects on the immune system. We sought to determine and compare the off-target effects of the adenovirus vector ChAdOx1-S (Oxford-AstraZeneca) and modified mRNA BNT162b2 (Pfizer-BioNTech) vaccines on immune responses to unrelated pathogens.

**Methods:**

Prospective sub-study within the BRACE trial. Blood samples were collected from 284 healthcare workers before and 28 days after ChAdOx1-S or BNT162b2 vaccination. SARS-CoV-2-specific antibodies were measured using ELISA, and whole blood cytokine responses to specific (SARS-CoV-2) and unrelated pathogen stimulation were measured by multiplex bead array.

**Findings:**

Both vaccines induced robust SARS-CoV-2 specific antibody and cytokine responses. ChAdOx1-S vaccination increased cytokine responses to heat-killed (HK) *Candida albicans* and HK *Staphylococcus aureus* and decreased cytokine responses to HK *Escherichia coli* and BCG. BNT162b2 vaccination decreased cytokine response to HK *E. coli* and had variable effects on cytokine responses to BCG and resiquimod (R848). After the second vaccine dose, BNT162b2 recipients had greater specific and off-target cytokine responses than ChAdOx1-S recipients.

**Interpretation:**

ChAdOx1-S and BNT162b2 vaccines alter cytokine responses to unrelated pathogens, indicative of potential off-target effects. The specific and off-target effects of these vaccines differ in their magnitude and breadth. The clinical relevance of these findings is uncertain and needs further study.

**Funding:**

10.13039/100000865Bill & Melinda Gates Foundation, 10.13039/501100000925National Health and Medical Research Council, 10.13039/100000001Swiss National Science Foundation and the Melbourne Children’s. BRACE trial funding is detailed in acknowledgements.

**Research in context**

**Evidence before this study**

Vaccines, particularly live-attenuated vaccines such as bacille Calmette–Guérin (BCG), have beneficial off-target effects including protection from unrelated infectious diseases. This is hypothesised to be mediated through immunoregulation including induction of trained immunity in innate immune cells such as monocytes. On 29th May 2023 we did a search in PubMed for articles investigating off-target effects of COVID-19 vaccines. PubMed search parameters: (1) “off target effects of covid-19 vaccines”. (2) (COVID-19 vaccine [Title/Abstract] AND trained immunity [Title/Abstract]) NOT (BCG [Title/Abstract]); (3) “Trained Immunity” [MeSH] AND “COVID-19/prevention and control” [MAJR]; (4) “Epigenetic Memory” [MeSH] AND “COVID-19/prevention and control” [MAJR].

From these searches there were 2 relevant primary research articles, and several other articles investigated or discussed (in reviews) the potential off-target effects of other routine vaccines (such as BCG) on protection against COVID-19. The two relevant studies were small pilot studies (n ≤ 10) investigating effects of COVID-19 vaccination on monocytes. The first, found transient epigenetic changes in monocytes one-two days after each dose of BNT162b2, but not four weeks after the second BNT162b2 vaccination dose. There were no statistically significant changes in cytokine responses to the viral Toll-like receptor (TLR)7/8 agonist resiquimod (R848) after either BNT162b2 vaccination dose. However, due to the small sample size (n = 4–5), the lack of sustained epigenetic changes or changes in cytokine responses may be the result of type II error. The second study found altered monocyte activation markers, metabolic gene expression and cytokine responses to irradiated *Mycobacterium tuberculosis*, TLR 1/2 or 3 agonists up to 12 weeks after the first ChAdOx1-S vaccination in ten healthy adults.

**Added value of this study**

Using samples from over 250 healthcare workers with no prior SARS-CoV-2 exposure, this study provides a robust investigation and comparison of the off-target effects of two of the most widely used COVID-19 vaccines worldwide. By investigating immune responses in whole blood samples (rather than a single immune cell sub-set), and by using inactivated/killed human pathogens (rather than focusing on TLR agonists), this *in vitro* study provides biologically relevant insights into potential immunomodulatory effects of COVID-19 vaccines to a sub-set of unrelated pathogens.

**Implications of all the available evidence**

For vaccines such as BCG, off-target immunomodulatory effects are proposed to underpin protection against unrelated infections and diseases (such as cancer). Since their development in 2020, COVID-19 vaccines have been administered to millions of people worldwide. Understanding potential off-target effects of these vaccines is important for assessing their overall impact on human health. The findings of *in vitro* immunomodulatory effects of COVID-19 vaccines suggest the potential for off-target effects on immune responses; however, without a defined minimal clinically important difference, it is not known whether these will translate to clinically relevant impacts on human health.

## Introduction

The COVID-19 pandemic fast-tracked the assessment and deployment of vaccines based on novel vaccine platforms, namely the mRNA and adenovirus vector-based vaccines. The mRNA-based BNT162b2 (Pfizer-BioNTech) and mRNA-1273 (Moderna) vaccines, and the replication-deficient adenovirus vector based ChAdOx1-S (Oxford-AstraZeneca) vaccines are among the first and most widely administered COVID-19 vaccines worldwide. These vaccines are highly effective in protecting against severe disease and hospitalisation due to COVID-19.^1^^,^^2^

The primary goal of vaccination is to induce antigen-specific or cross-protective immune responses against a target pathogen. However, several vaccines, particularly live-attenuated vaccines, have beneficial off-target effects which include protection against unrelated infectious diseases and tumour recurrence.^3^, ^4^, ^5^, ^6^, ^7^ For example, bacille Calmette-Guérin (BCG), the live-attenuated tuberculosis vaccine, and measles-containing vaccines reduce all-cause mortality in children under 5 years of age in high-mortality settings by 30–53% and 26–49% respectively.^4^ This protection is proposed to result from enhancement of immune responses to, and thus protection against, unrelated pathogens.^6^^,^^8^, ^9^, ^10^, ^11^, ^12^, ^13^, ^14^ Studies of the underlying mechanisms for this off-target immune boosting by vaccines suggest changes in both innate and adaptive immune responses.^3^^,^^8^^,^^10^, ^11^, ^12^^,^^15^ For BCG vaccine, changes in innate immune responses are associated with epigenetic reprogramming and a metabolic shift in innate immune cells, termed trained immunity.^8^^,^^16^^,^^17^ However, as anti-pathogen immune responses can contribute to infectious disease symptoms, this off-target boosting of immune responses may also increase symptoms following infections with unrelated pathogens.^18^^,^^19^

With widespread administration of COVID-19 vaccines, potential off-target immunological effects have important implications for global health. A preliminary study in ten healthy adults found changes in monocytes consistent with trained immunity following ChAdOx1-S vaccination.^20^ A second study in up to five healthcare workers found transient epigenetic and transcriptional changes in monocytes days after BNT162b2 vaccination.^21^ These studies suggest potential induction of trained immunity by COVID-19 vaccines. We hypothesise that the mRNA-based BNT162b2 and adenovirus vector-based ChAdOx1-S COVID-19 vaccines have off-target effects on immune responses to unrelated pathogens and that the nature and magnitude of these effects, will differ between the two vaccine platforms.

**Fig. 1:**
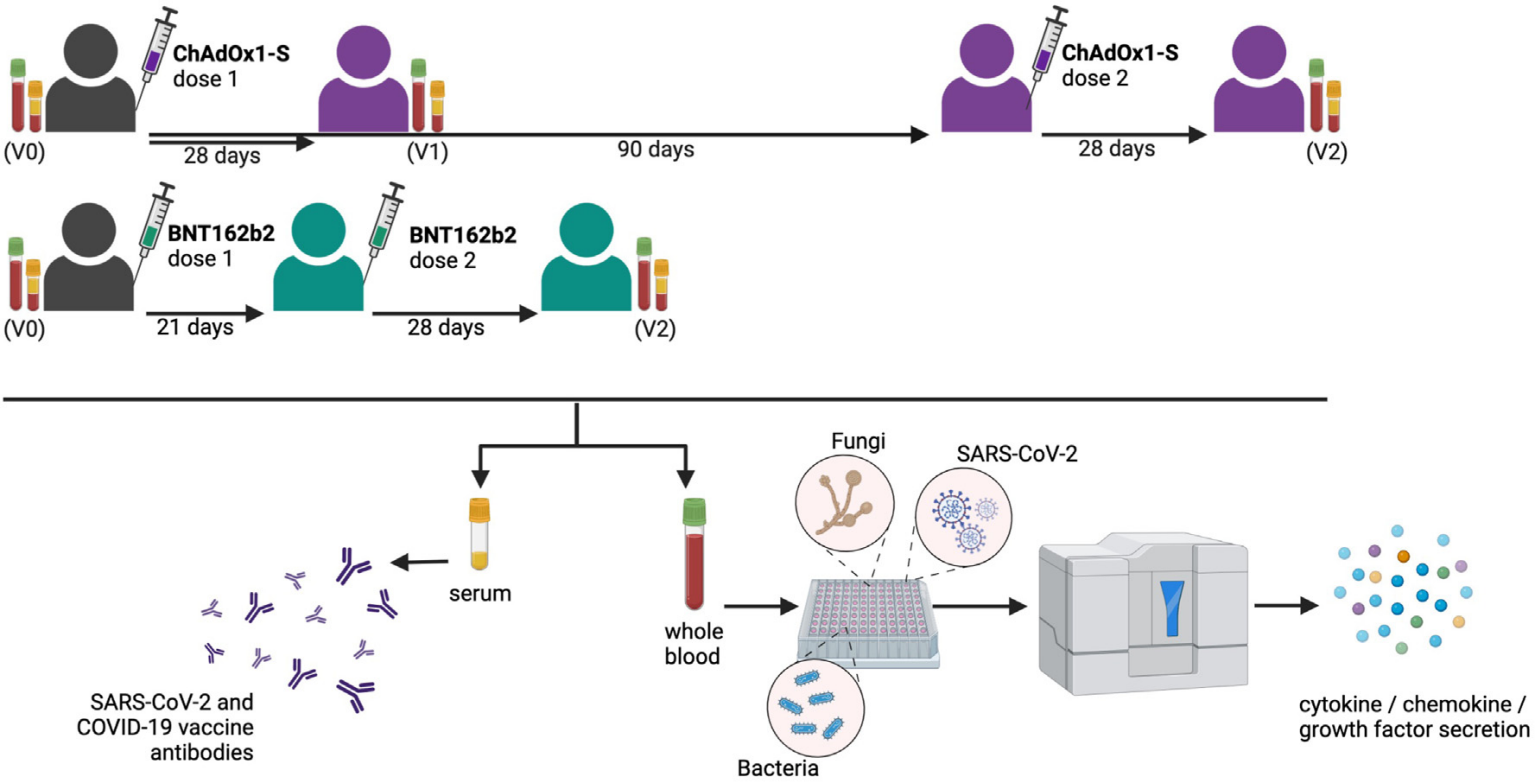
**BCOS study schema.** Blood samples were collected from participants prior to the first dose of a COVID-19 vaccination (V0), 28 (±3 days) after the first dose of a ChAdOx1-S (V1) and 28 (±3 days) after the second dose of ChAdOx1-S or BNT162b2 (V2). Created with BioRender.com.

In this study, we recruited a sub-set of participants from a multicentre, randomised controlled trial (RCT) of BCG vaccination to reduce the impact of COVID-19 in healthcare workers (BRACE trial)^19^^,^^22^ and compared the off-target immunological effects of the ChAdOx1-S and BNT162b2 vaccines (Fig. 1).

Understanding these effects will help to provide insights into the mechanisms of action of COVID-19 vaccines and may also inform the development of future vaccine strategies against emerging infectious diseases and other health conditions.

## Methods

### Study design

This study is an exploratory prospective sub-study of participants in the BRACE trial (NCT04327206), a RCT of BCG vaccination to reduce the impact of COVID-19 in healthcare workers.

### Participants

BRACE trial participants were recruited to the ‘BRACE COVID-19-specific vaccine’ (BCOS) sub-study from BRACE trial study sites in Australia (Victoria and South Australia) and Brazil. The full BRACE trial protocol is available at clincaltrials.gov^23^ and a brief description is provided in the Supplementary methods. The BRACE trial recruited participants in two stages. In stage 1, participants were randomised 1:1 to receive a single intradermal dose of 0.1 mL of BCG-Denmark vaccine (AJ Vaccines; Danish strain 1331) or no BCG. In stage 2, participants were randomised 1:1 to receive a single intradermal dose of 0.1 mL of BCG-Denmark vaccine (AJ Vaccines; Danish strain 1331) or saline placebo.

For the BCOS sub-study, BRACE trial participants were eligible for inclusion if they had consented to be contacted for future ethically approved projects and were recruited to the BRACE trial at a study site participating in the BCOS sub-study. Participants were excluded from the BCOS sub-study if they expected to be unable to provide a blood sample 28 days after the first or second dose of a COVID-19 vaccination, and in Australia, if they had a positive SARS-CoV-2 test at any time prior to recruitment to BCOS. There were no inclusion or exclusion criteria related to the sex/gender of participants or randomisation group (i.e., BCG vaccination or control group) in the BRACE trial. The study described henceforth includes only BCOS participants from Victoria (whole blood stimulation and serology) and South Australia (serology).

The sample size for this sub-study was chosen pragmatically, with the inclusion of all BRACE trial participants who met the inclusion criteria, did not meet exclusion criteria and were willing to provide vaccination data and peripheral blood samples for the study.

### Sample collection

Peripheral blood samples were collected between 2nd March 2021 and 24th September 2021. Blood samples were collected from participants prior to the first dose of the ChAdOx1-S or BNT162b2 vaccine (Visit 0, V0), 28 (±3 days) after the first dose of the ChAdOx1-S vaccine (V1) and 28 (±3 days) after the second dose (V2) of the ChAdOx1-S or BNT162b2 vaccine (Fig. 1). Participants received their COVID-19 vaccinations through the Australian COVID-19 vaccination program. The recommended 21-day interval between the first and second dose of BNT162b2 at the time of this study precluded collection of V1 samples from BNT162b2 recipients.

For BCOS, peripheral blood was collected in CAT Serum Separator Clot Activator tubes (Greiner bio-one) and sodium heparin tubes (BD Biosciences). In addition, peripheral blood was collected in lithium heparin tubes (Greiner bio-one) from BRACE trial participants at randomisation and at three-month intervals throughout the trial.^19^

### Serology testing

Serum and plasma samples were enriched by centrifugation then stored at −80 °C prior to testing. Where a V0 serum sample was not available, the participant’s most recent plasma sample available prior to COVID-19 vaccination (from BRACE trial 3-monthly samples) was used in this study (n = 7). Anti-SARS-CoV-2 spike and anti-SARS-CoV-2 receptor binding domain (RBD) IgG in serum and plasma samples were quantified by ELISA as previously described.^24^^,^^25^ Anti–SARS-CoV-2 nucleocapsid (NCP) total antibodies were assessed using the Cobas Elecsys Anti-SARS-CoV-2 assay as per manufacturer’s instructions (Roche).^26^

### Stimulants

Preparation of stimuli was described previously.^12^^,^^15^ The final concentrations of stimuli in the assay strips were: RPMI 1:2, 1:10 γ-irradiated (50 kGy) mock infected vero-cell supernatants (iVero), 1:10 γ-irradiated (50 kGy) SARS-CoV-2 infected vero-cell supernatants (iSARS) 10^6.2^ TCID_50_/mL, BCG-Denmark (Serum Statens Institut, Denmark) 75 μg/mL, heat-killed (HK) *Candida albicans* 1.0 × 10^6^ CFU/mL, HK *Escherichia coli* 1.0 × 10^6^ CFU/mL, HK *Staphylococcus aureus* 1.0 × 10^7^ CFU/mL and resiquimod (R848) 3.5 μg/mL.

### Whole blood stimulation (WBS)

Whole blood, diluted 1:1 with RPMI 1640 medium (GlutaMAX Supplement, HEPES, Gibco, Life Technologies), was added to pre-prepared stimulation assay strips. When insufficient blood was available for all stimulations, a predetermined priority order was used. Whole blood was stimulated at 37 °C (5% CO_2_) for 20 (±2) hours. Following stimulation, samples were centrifuged then supernatants were harvested and stored at −80 °C for future cytokine analysis.

### Cytokine analysis

Supernatants from stimulated whole blood were diluted 1:5 prior to quantification of secreted cytokines, chemokines and growth factors using the Bio-Plex Pro Human Cytokine 48-Plex Screening Panel (Bio-Rad) according to the manufacturer’s instructions. The following analytes were measured: CTACK, Eotaxin, Basic FGF, G-CSF, GM-CSF, GRO-α, HGF, IFN-α2, IFN-γ, IL-1α, IL-1β, IL-1Ra, IL-2, IL-2rα, IL-3, IL-4, IL-5, IL-6, IL-7, IL-8, IL-9, IL-10, IL-12p40, IL-12p70, IL-13, IL-15, IL-16, IL-17, IL-18, IP-10, LIF, MCP-1, MCP-3, M-CSF, MIF, MIG, MIP-1α, MIP-1β, β-NGF, PDGF-BB, RANTES, SCF, SCGF-β, SDF-1α, TNF-α, TNF-β, TRAIL, VEGF. Data was acquired on the Bio-Plex 200 system using BioPlex Manager™ 6.1 Software (Bio-Rad).

### Statistical analysis

For paired analysis, the outcomes were changes in: (i) cytokine responses to *in vitro* stimulation; and (ii) antibody levels after compared to before COVID-19 vaccination. For unpaired analysis, the outcomes were differences in: (i) cytokine responses to *in vitro* stimulation; and (ii) antibody levels 28 days after the second COVID-19 vaccination dose compared between ChAdOx1-S and BNT162b2 recipients.

Statistical analysis was done using Stata version 17.0 (StataCorp LLC, USA) and R^27^ using R Studio (2022.07.1 + 54), and depicted graphically with R using R Studio (2022.07.1 + 54) and GraphPad Prism (version 9.1.0). Values below the lower limit of detection for each analyte were assigned a value of half the lowest detected value for that analyte and values above the upper limit of detection were excluded for that analyte.^11^ Proportion of stimulations above the upper limit of detection were IL-1β 0.04%, IL-6 3.06%, IL-8 5.34%, IP-10 0.04%, MCP-1 15.19%, MIP-1α 6.39%, and MIP-1β 28.02%. A sensitivity analysis for was done for cytokine–stimulant pairs with >1% values above the upper limit of detection, wherein these missing values were assigned a value of double the highest detected value for that analyte. The remaining cytokines had all values below the upper limit of detection.

Participants were excluded from analysis if they had positive NCP-serology, or a pre-vaccination sample with no post-vaccination samples (within the blood collection window). Participants were excluded from whole blood stimulation analysis if they had a post-vaccination with no pre-vaccination sample. Samples were excluded if blood was taken outside of the blood collection window (Supplementary Fig. S1). Complete-case analysis was done for both paired and unpaired data. Prior to analysis, distribution of the data was checked with and without log-transformation. Where log-transformation resulted in normal distribution, parametric tests were done on the log-transformed data. Where log-transformation did not result in normal distribution, non-parametric tests were done on the non-transformed data. As this is an exploratory study, correction for multiple comparisons was not done so as to maximise identification of trends and due to the expected relatedness of outcomes (e.g., IFN responses would be related to responses for IFN-induced cytokines).

For paired analyses, only participants with matched V0 (pre-COVID-19 vaccination) and V1 or V2 were included. The change in anti-spike and anti-RBD IgG after ChAdOx1-S or BNT162b2 vaccination was determined by paired t-test of the log-transformed area under the curve (AUC) comparing V1 or V2 with V0, or V2 with V1 in paired samples. Changes in cytokine responses were assessed by Wilcoxon signed-rank test (WSR) or sign test (ST), depending on symmetry of distribution for differences (symmetrical analysed by WSR, asymmetrical analysed by ST).^28^^,^^29^ Symmetry of distribution for differences in paired data (V1–V0 or V2–V0) was assessed by determining the skewness using Stata sktest, skewness value and review of difference plots. For control samples (nil/unstimulated and iVero), concentrations were compared between samples from V1 or V2 and paired V0 samples. For stimulated samples (BCG, *C. albicans*, *E. coli*, iSARS, *S. aureus* and R848), stimulation effect (stimulant minus nil or iSARS minus iVero) was compared between samples from V1 or V2 and paired V0 samples. This was done separately for recipients of ChAdOx1-S and BNT162b2. For ChAdOx1-S recipients, there were insufficient paired V0 and V2 *in vitro* cytokine response samples for WBS analysis.

Differences in immune responses at V2 between ChAdOx1-S and BNT162b2 recipients were assessed using linear regression of log-transformed antibody and cytokine data adjusted for: sex, age at first COVID-19 vaccine dose, days between second COVID-19 vaccine dose and V2 blood collection, BCG vaccination prior to BRACE trial, BCG vaccination as part of BRACE trial, and BRACE trial stage (stage 1 or 2), and additional for cytokine data: pre-prepared stimulant batch and control (nil or iVero) cytokine responses. Due to non-normal distribution after log transformation, IL-3 and IL-7 were analysed by bootstrapped quantile regression of the raw cytokine data with adjustments as above for the linear regression. Sub-group analyses done separately for participants who were and were not BCG-vaccinated in the BRACE trial.

For correlation analysis of V1 and V2 IgG and cytokine data, data was log transformed and similarity was measured by Pearson correlation using the Hmisc package in R and depicted graphically using the ggpubr and corrplot packages.

For correlation analysis of paired IgG and cytokine data stimulated effect data, differences (V1 or V2 minus V0) in IgG and cytokine stimulation effect were calculated and participants with >10 missing data points were excluded. Similarity was measured by Spearman correlation using the Hmisc package, unsupervised hierarchical clustering using these coefficients as distance was done using hlcust, and data was depicted graphically using the ggpubr and ComplexHeatmap packages.

### Ethics

The BRACE trial, which includes the BCOS sub-study, was approved by the Royal Children’s Hospital Human Research Ethics Committee (HREC) (No. 62586) with HREC and/or governance approval at all participating sites. Signed informed consent was obtained from all participants.

### Role of funders

Funders had no role in study design, collection, analyses and interpretation of data or in the preparation, review or approval of the manuscript.

## Results

Of 284 participants in Australia with blood samples collected for BCOS, 264 had samples eligible for inclusion. Of these, 65 and 59 participants had samples eligible for paired pre-vaccination (V0) to 28 days post dose 1 (V1) serology and whole blood stimulation analysis respectively, and 122 and 19 had samples eligible for paired pre-vaccination to 28 days post dose 2 (V2) serology and whole blood stimulation analysis respectively (Supplementary Fig. S1). For unpaired analysis of V2 samples, 225 and 115 participants had samples eligible for serology and whole blood stimulation analysis respectively. Overall participants were more likely to be female (77.9%) and have previously received BCG (86.8%), and these were similar between the COVID-19 vaccination groups (Table 1, Supplementary Table S1). The median age of participants included in paired whole blood stimulation analysis was 48 (interquartile range (IQR) 39–58) years with an older age amongst ChAdOx1-S recipients (median 49 (IQR 39–58) years) compared to BNT162b2 recipients (median 43 (IQR 33–50) years) (Table 1). Demographics for participants included in the unpaired analysis are in Supplementary Table S1.

**Table 1:** BRACE trial participants in the BCOS sub-study in Australia with pre and post vaccination samples included in the paired analysis.

	Serology			Whole blood stimulation	
	ChAdOx1-S V0 & V1 n = 65	ChAdOx1-S V0 & V2 n = 67	BNT162b2 V0 & V2 n = 55	ChAdOx1-S V0 & V1 n = 59	BNT162b2 V0 & V2 n = 19
Age (years) at 1st COVID-19 vaccination dose, median (IQR)					
	51 (39–58)	51 (40–59)	47 (37–56)	49 (39–58)	43 (33–50)
Sex					
Male	14 (21.5%)	18 (26.9%)	12 (21.8%)	12 (20.3%)	3 (15.8%)
Female	51 (78.5%)	49 (73.1%)	43 (78.2%)	47 (79.7%)	16 (84.2%)
Any COVID-19 comorbidities^a^					
Yes	9 (13.8%)	13 (19.4%)	13 (23.6%)	10 (16.9%)	5 (26.3%)
Diabetes	1 (1.5%)	0	0	1 (1.7%)	0
Cardiovascular disease	7 (10.8%)	9 (13.4%)	8 (14.5%)	7 (11.9%)	3 (15.8%)
Chronic respiratory disease	1 (1.5%)	5 (7.5%)	5 (9.1%)	2 (3.4%)	2 (10.5%)
No	56 (86.2%)	54 (80.6%)	42 (76.4%)	49 (83.1%)	14 (73.7%)
Obesity					
Yes	10 (15.4%)	12 (17.9%)	8 (14.5%)	10 (16.9%)	4 (21.1%)
No	50 (76.9%)	51 (76.1%)	42 (76.4%)	45 (76.3%)	15 (78.9%)
Missing	5 (7.7%)	4 (6.0%)	5 (9.1%)	4 (6.8%)	0
Smoking					
Yes	4 (6.2%)	3 (4.5%)	3 (5.5%)	5 (8.5%)	0
No	61 (93.8%)	64 (95.5%)	52 (94.5%)	54 (91.5%)	19 (100.0%)
Occupation					
Allied health	3 (4.6%)	2 (3.0%)	2 (3.6%)	2 (3.4%)	1 (5.3%)
Clerical/Administrative	7 (10.8%)	7 (10.4%)	3 (5.5%)	6 (10.2%)	2 (10.5%)
Doctor	8 (12.3%)	11 (16.4%)	4 (7.3%)	6 (10.2%)	12 (63.2%)
Nurse/Midwife	25 (38.5%)	22 (32.8%)	32 (58.2%)	25 (42.4%)	4 (21.1%)
Other role	22 (33.8%)	25 (37.3%)	13 (23.6%)	20 (33.9%)	0
Patient service assistant	0	0	1 (1.8%)	0	0
BCG-vaccinated prior to BRACE trial					
No	30 (46.2%)	28 (41.8%)	24 (43.6%)	27 (45.8%)	10 (52.6%)
Yes	35 (53.8%)	39 (58.2%)	31 (56.4%)	32 (54.2%)	9 (47.4%)
BCG-vaccinated in BRACE trial					
No	18 (27.7%)	18 (26.9%)	20 (36.4%)	17 (28.8%)	7 (36.8%)
Yes	47 (72.3%)	49 (73.1%)	35 (63.6%)	42 (71.2%)	12 (63.2%)
Any other vaccinations between V0 and post COVID-19 vaccination blood					
Yes	3 (4.6%)	9 (13.4%)	9 (16.4%)	3 (5.1%)	1 (5.3%)
Age (years) at 1st COVID-19 vaccination dose, median (IQR)					
	51 (39–58)	51 (40–59)	47 (37–56)	49 (39–58)	43 (33–50)
Days between 1st and 2nd COVID-19 vaccination doses, median (IQR)					
	87 (84–91)	87 (84–91)	22 (21–25)	87 (84–91)	22 (21–27)
Days between COVID-19 vaccination dose and post blood, median (IQR)					
Dose 1 and V1	28 (27–28)	–	–	28 (27–28)	–
Dose 2 and V2	–	28 (28–28)	28 (28–28)	–	28 (27–28)
Days between pre blood (V0) and post blood, median (IQR)					
	29 (28–32)	119 (113–126)	53 (50–57)	29 (28–32)	51 (49–56)

a At BRACE trial randomisation: Diabetes (any type), cardiovascular disease (including hypertension) or chronic respiratory disease (including asthma and chronic obstructive pulmonary disease).

### Changes in immune responses following ChAdOx1-S vaccination

**Fig. 2:**
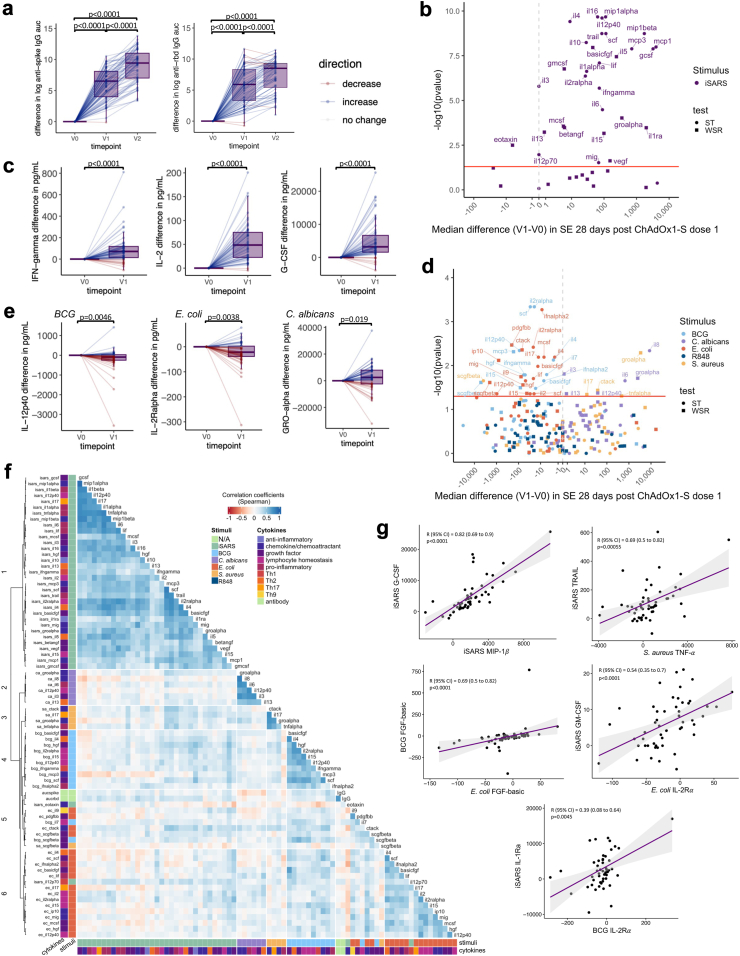
**SARS-CoV-2-specific and off-target effects of ChAdOx1-S.** Differences in immune responses 28 (±3 days) after the first (V1) or second (V2) dose of ChAdOx1-S compared to pre-vaccination (V0) samples. (a) Tukey boxplots and dot plot overlay depicting differences (V1/V2—V0) in log transformed anti-spike and anti-RBD IgG AUC (n = 80). Statistically significant differences before and after ChAdOx1-S vaccination were determined by paired-t test. (b–e) Volcano and Tukey boxplots (with dot plot overlay) depicting differences in (b and c) iSARS stimulation effect (iSARS—iVero response) (n = 55) and (d and e) BCG, *C. albicans*, *E. coli*, *S. aureus* and R848 stimulation effect (stimulant—nil) (n = 57–56) on whole blood cytokine responses. p-value for differences before (V0) and after the first dose of ChAdOx1-S vaccination (V1) were determined by Wilcoxon signed-rank test (WSR, squares) or sign-rank test (ST, circles) as indicated in Supplementary Table S4. (b & d) Volcano plots: Red line indicates p = 0.05 by WSR or ST. (a, c, e) Boxplots: centre lines indicate medians; box limits indicate 25th–75th percentiles; whiskers extend to 1.5 times the interquartile range from the 25th and 75th percentiles. Dots represent each individual participant with line colours representing direction of change relative to the previous datapoint (blue = increase, red = decrease, white = no change). (f) Spearman’s correlation coefficients of differences (V1–V0) in serum levels (IgG) and whole blood cytokine stimulation effect that showed change after ChAdOx1-S vaccination and had a p < 0.05 by WSR or ST. Unsupervised hierarchical clustering was done using Spearman’s correlation as the measure of similarity between cytokine–stimulant pairs. Red indicates a negative correlation, whereas blue indicates a positive correlation. The data shown are from participants with <10 missing paired results (n = 52). (g) Scatter plots of differences (V1—V0) in stimulation effect between cytokine–stimulant pairs. Correlation coefficient (R) and p-value determine by Spearman’s correlation. Purple line depicts regression line, grey indicates 95% confidence interval (CI). Abbreviations: auc, area under the curve; BCG or bcg, bacille Calmette Guérin; ca, *C. albicans*; ec, *E. coli*; Ig, immunoglobulin; iSARS (isars), γ-irradiated SARS-CoV-2; R848 or r848, resiquimod; RBD, receptor binding domain; sa, *S. aureus*; SE, stimulation effect, Th, T helper.

Following vaccination with ChAdOx1-S, serum anti-SARS-CoV-2 spike and anti-RBD IgG levels were, as expected, higher 28 days after the first and second vaccination doses (Fig. 2A). Analysis of cytokines in unstimulated (nil/RPMI only) and iVero stimulated whole blood revealed changes (generally small increases) in cytokine secretion before (V0) compared to 28 days after the first dose (V1) of ChAdOx1-S (Supplementary Table S2). Due to these changes in the unstimulated samples following the first dose of ChAdOx1-S, assessment of changes in stimulated samples was done using the stimulation effect (stimulant minus nil or stimulant minus iVero). As expected, compared to before vaccination, 28 days after the first dose of ChAdOx1-S, cytokine responses to stimulation with iSARS were increased (Fig. 2B and C, Supplementary Table S4). The exceptions being responses to eotaxin and IFN-***α***2, which were more compatible with a model of decreased response to iSARS. Notably, although anti-spike and anti-RBD IgG, and the majority of iSARS-induced cytokine and chemokine responses increased after the first dose of ChAdOx1-S, there was limited positive correlation between the IgG and iSARS whole blood stimulation responses after the first and second doses of ChAdOx1-S (Supplementary Fig. S2A–D).

To determine if ChAdOx1-S influenced immune responses to unrelated pathogens, we compared cytokine responses following *in vitro* stimulation of whole blood collected before and 28 days after the first ChAdOx1-S dose. Responses to BCG and *E. coli* were compatible with a model of lower cytokine secretion following the first dose of ChAdOx1-S vaccine compared to responses pre-vaccination (Fig. 2D and E, Supplementary Table S4). These included cytokines involved in T cell responses (IFN-γ, IL-4, IL-5, IL-9, IL-12p70, IL-17), lymphocyte homeostasis (IL-2, IL-2R***α***, IL-7, IL-12p40, IL-15), pro-inflammatory responses (IFN-***α***2, IL-6, LIF, TNF-***α***), growth factors (FGF-basic, HGF, MCS-F, SCF, SCGF-***β***, PDGF-BB, VEGF) and chemokines/chemoattractants (CTACK, IL-16, IP-10, MCP-3, MIG, SDF-1***α***). Responses to *C. albicans* and *S. aureus* stimulation were compatible with a model of higher cytokine secretion following the first dose of ChAdOx1-S compared to responses pre-vaccination (Fig. 2D and E, Supplementary Table S4). These included cytokines involved in T cell responses (IL-13, IL-17), lymphocyte homeostasis (IL-12p40), pro-inflammatory responses (IL-1***α***, IL-6, TNF-***α***), anti-inflammatory responses (IL-10), growth factors (IL-3) and chemokines/chemoattractants (CTACK, GRO-***α***, IL-8). Responses to R848 stimulation were not compatible with a model of altered cytokine secretion following the first dose of ChAdOx1-S compared to responses pre-vaccination (Fig. 2D and E, Supplementary Table S4).

To determine if stronger specific responses were associated with stronger off-target responses, we assessed the correlations between the cytokine–stimulant pairs (and IgG serology) that were compatible (to the level of p < 0.05) with a model of altered cytokine responses 28 days after the first dose of ChAdOx1-S. Consistent with our previous study, we found that responses clustered within stimulant groups rather than by cytokine (Fig. 2F).^15^ There were strong positive correlations between different iSARS-induced cytokines with the exception IL-12p70 and eotaxin (Fig. 2F and G). Associations between changes in iSARS stimulation-induced cytokine responses and anti-RBD IgG responses were weak although, stronger than those observed for anti-spike IgG (Fig. 2F). Despite lower cytokine responses to BCG and *E. coli* following the first dose of ChAdOx1-S compared to responses pre-vaccination, there were clusters of cytokines for which there was a positive correlation of BCG or *E. coli* responses with the iSARS cytokine responses (Fig. 2F and G). For iSARS-induced responses, these was most strongly observed for GM-CSF, GRO-***α***, IFN-γ, IL-1ra, IL-2, IL-5, IL-12p70, IL-15, MCP-1, MCP-3, M-CFS and SCF. For BCG stimulation, this was most strongly observed for IL-2r***α***, IL-4, IL-5, IL-12p40, HGF and SCF. For *E. coli-*induced responses, these was most strongly observed for CTACK, IL-2, and IL-2r***α*** (Fig. 2F). For eotaxin-induced responses to iSARS, which were lower after the first dose of ChAdOx1-S compared to responses pre-vaccination, there was a pattern of negative correlation to *E. coli* responses (Fig. 2F). Changes in responses to *C. albicans* showed weak positive correlations with iSARS and *E. coli* responses. Whereas changes in responses to *S. aureus* showed stronger positive correlation with iSARS stimulations for all *S. aureus*-induced cytokines assessed (Fig. 2F). The strongest iSARS correlations with *S. aureus*-induced responses were for basic-FGF, MCP-3, M-CSF, SCF and TRAIL.

### Changes in immune responses following BNT162b2 vaccination

**Fig. 3:**
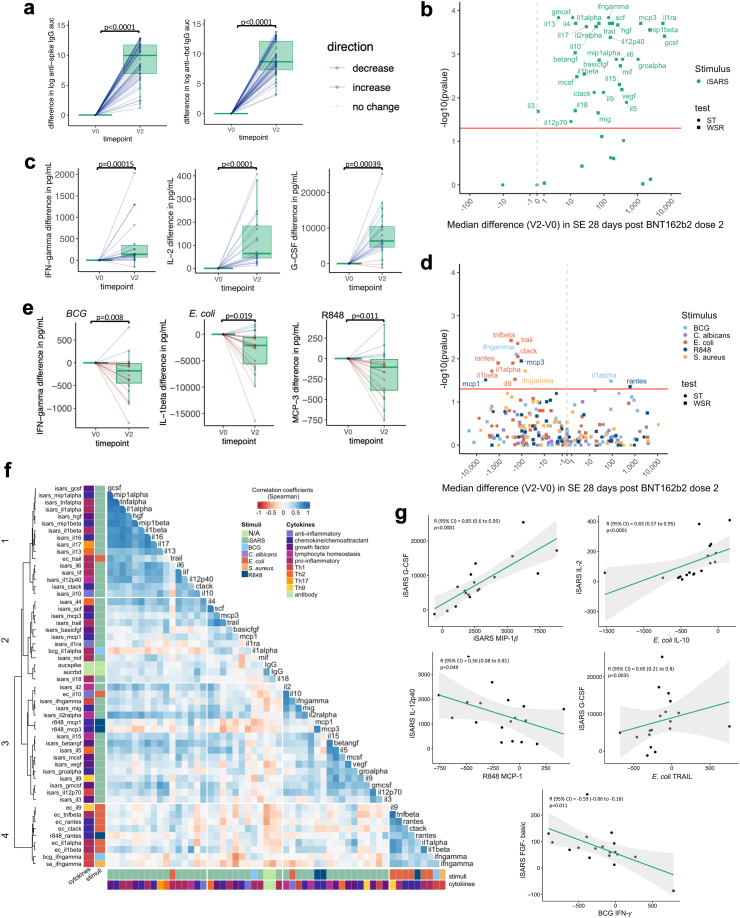
**SARS-CoV-2-specific and off-target effects of BNT162b2.** Differences in immune responses 28 (±3 days) after the second (V2) dose of BNT162b2 compared to pre-vaccination (V0) samples. (a) Tukey boxplots and dot plot overlay depicting differences (V2—V0) in log transformed anti-spike and anti-RBD IgG AUC (n = 55). Statistically significant differences before and after BNT162b2 vaccination were determined by paired-t test. (b–e) Volcano and Tukey boxplots (with dot plot overlay) depicting differences in (b and c) iSARS stimulation effect (iSARS—iVero response) (n = 18) and (d and e) BCG, *C. albicans*, *E. coli*, *S. aureus* and R848 stimulation effect (stimulant—nil) (n = 19) on whole blood cytokine responses. p-value for differences before (V0) and after the first dose of BNT162b2 vaccination (V2) were determined by Wilcoxon signed-rank test (WSR, squares) or sign-rank test (ST, circles) as indicated in Supplementary Table S5. (b & d) Volcano plots: Red line indicates p = 0.05 by WSR or ST. (a, c, e) Boxplots: centre lines indicate medians; box limits indicate 25th–75th percentiles; whiskers extend to 1.5 times the interquartile range from the 25th and 75th percentiles. Dots represent each individual participant with line colours representing direction of change relative to the previous datapoint (blue = increase, red = decrease, white = no change). (f) Spearman’s correlation coefficients of differences (V2–V0) in serum levels (IgG) and whole blood cytokine stimulation effect that showed a change after BNT162b2 vaccination and had a p < 0.05 by WSR or ST. Unsupervised hierarchical clustering was done using Spearman’s correlation as the measure of similarity between cytokine–stimulant pairs. Red indicates a negative correlation, whereas blue indicates a positive correlation. The data shown are from participants with <10 missing paired results (n = 18). (g) Scatter plots of differences (V2—V0) in stimulation effect between cytokine–stimulant pairs. Correlation coefficient (R) and p-value determine by Spearman’s correlation. Green line depicts regression line, grey indicates 95% confidence interval (CI). Abbreviations: auc, area under the curve; BCG or bcg, bacille Calmette Guérin; ca, *C. albicans*; ec, *E. coli*; Ig, immunoglobulin; iSARS (isars), γ-irradiated SARS-CoV-2; R848 or r848, resiquimod; RBD, receptor binding domain; sa, *S. aureus*; SE, stimulation effect, Th, T helper.

Following vaccination with BNT162b2, serum anti-spike and anti-RBD IgG levels were higher 28 days after second vaccination dose (Fig. 3A). Analysis of cytokines in unstimulated and iVero stimulated whole blood revealed small changes in cytokine secretion 28 days after the second dose of BNT162b2 (Supplementary Table S3). Therefore, as for ChAdOx1-S, changes in stimulated samples after BNT162b2 were assessed using the stimulation effect (stimulant minus nil or stimulant minus iVero). As expected, compared to before vaccination, 28 days after the second dose of BNT162b2, cytokine responses to stimulation with iSARS were increased (Fig. 3B and C, Supplementary Table S5). Similar to responses to two doses of ChAdOx1-S, there was limited correlation between the IgG and iSARS whole blood stimulation responses after the second doses of BNT162b2 (Supplementary Fig. S2E and F).

Consistent with the changes seen after the first ChAdOx1-S dose, responses to *E. coli* were compatible with a model of lower cytokine secretion 28 days after the second dose of BNT162b2 vaccine compared to responses pre-vaccination (Fig. 3D and E, Supplementary Table S5). These included cytokines involved in T cell responses (IL-9), pro-inflammatory responses (IL-1***α***, IL-1***β***, TNF-***β***, TRAIL) and chemokines/chemoattractants (CTACK, RANTES). Responses to BCG, R848 and *S. aureus* stimulation showed some compatibility with a model of altered cytokine responses 28 days after the second dose of BNT162b2 vaccination compared to responses pre-vaccination. However, there was no consistent direction of change within each stimulus. Responses to *C. albicans* stimulation were not compatible with a model of altered cytokine secretion following the second dose of BNT162b2 compared to responses pre-vaccination (Fig. 2D and E, Supplementary Table S4).

To determine if stronger specific responses were associated with stronger off-target responses to BNT162b2, we assessed the correlation between all cytokine–stimulant pairs (and IgG serology) were compatible (to the level of p < 0.05) with a model of altered cytokine responses 28 days after the second dose of BNT162b2. There were positive correlations between iSARS-induced responses although this tended to be weaker for the sub-set of iSARS-induced cytokine responses in clusters 2 and 3 (Fig. 3F and G). There were generally weak positive and negative correlations between changes in iSARS and off-target (*E. coli*, R848, BCG and *S. aureus*) responses. Positive correlations were strongest for *E. coli*-induced TRAIL and IL-10 which clustered with iSARS-induced responses.

### Differences in off-target effects of ChAdOx1-S and BNT162b2 vaccination

To determine if there was a difference in immune responses following ChAdOx1-S and BNT162b2 vaccination we compared both SARS-CoV-2-specific and off-target immune responses 28 days after the second dose of each vaccine. Amongst the sub-set of participants with a serology (n = 226) or whole blood stimulation cytokine response (n = 110) data available from samples taken 28 days after the second COVID-19 vaccination dose, ChAdOx1-S recipients tended to be older, have fewer comorbidities and more had received BCG vaccination in the BRACE trial (Table 1).

**Fig. 4:**
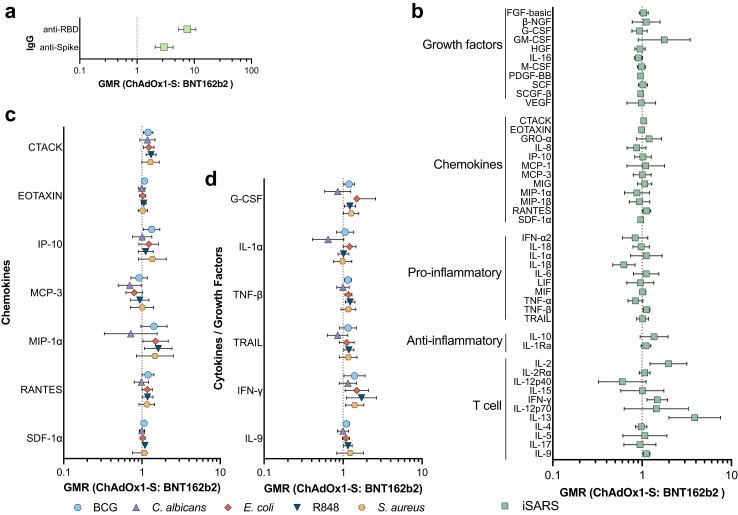
**Comparison of SARS-CoV-2-specific and off-target *in vitro* immune responses following BNT162b2 and ChAdOx1-S vaccination.** Forest plots depicting adjusted geometric mean ratios (GMR) and 95% confidence intervals for the effect of COVID-19 vaccine type on (a) serum IgG (n = 225) (b) iSARS-induced (n = 76–110) and (c and d) BCG (n = 37–115), *C. albicans* (n = 71–115), *E. coli* (n = 21–115), *S. aureus* (n = 70–115) and R848 (n = 24–115) -induced whole blood cytokine and chemokine responses 28 days after the second vaccination dose (V2). (c and d) A sub-set of (c) 7 chemokines and (d) 6 cytokines/growth factors are depicted, data for all cytokine–stimulant pairs are available in Supplementary Table S6. GMR > 1.0 indicates responses that were higher for BNT162b2-vaccinated compared to ChAdOx1-S-vaccinated participants. Abbreviations: BCG, bacille Calmette Guérin; GMR, geometric mean ratio; Ig, immunoglobulin; iSARS, γ-irradiated SARS-CoV-2; R848, resiquimod; RBD, receptor binding domain.

Recipients of BNT162b2 had higher serum anti-spike and anti-RBD IgG 28 days after the second vaccination dose than ChAdOx1-S recipients (Fig. 4A, Supplementary Fig. 3A and Table S6). For a sub-set of cytokines there was also greater iSARS-induced secretion of cytokines 28 days after the second vaccination dose of BNT162b2 than ChAdOx1-S (Fig. 4B, Supplementary Fig. 3B and Table S7). These higher responses were strongest for cytokines involved in T-cell pathways and homeostasis (IFN-γ, IL-2, IL-9, and IL-13). Few cytokines (including IL-1***β***) supported a model of lower responses following iSARS stimulation in BNT162b2 recipients compared to ChAdOx1-S recipients. For stimuli unrelated to SARS-CoV-2, there was a sub-set of cytokines/chemokines for which the data were consistent with a model of differential immune responses between BNT162b2 recipients and ChAdOx1-S recipients 28 days after the second dose of these vaccines (Fig. 4C and D, Supplementary Fig. 3C and Table S7). For BCG, *E. coli* and R848 stimulation, the data were consistent with a model of moderately higher responses amongst BNT162b2 recipients compared to ChAdOx1-S recipients after the second vaccination dose. Responses to *C. albicans* and *S. aureus* were generally similar between BNT162b2 recipients and ChAdOx1-S recipients and with geometric mean ratios (GMRs) ranging from little or no effect to a moderately higher (for *S. aureus*) or lower (*C. albicans*).

To determine if these observed differential specific and off-target effects of ChAdOx1-S and BNT162b2 vaccination were impacted by BCG vaccination within the past year, we did a sensitivity analysis in the sub-set of participants who did not receive BCG vaccination as part of the BRACE trial (Supplementary Table S8). A similar pattern of differential effects of ChAdOx1-S and BNT162b2 were observed in the sub-group of non-BCG vaccinated and the sub-group of BCG vaccinated participants, although the GMR was often smaller and with confidence intervals crossing one (Supplementary Tables S6–S9), likely due to the reduced participant numbers (n = 74 for serology and n = 36 for whole blood stimulations).

## Discussion

In this study, replication-deficient adenovirus ChAdOx1-S vaccine and mRNA BNT162b2 vaccine changed the hosts’ immune responses to unrelated pathogens. These off-target effects were pathogen dependent and divergent between the two vaccine platforms.

Live-attenuated vaccines, such as BCG and measles-containing vaccines, have off-target effects on clinical and immunological outcomes.^3^^,^^4^^,^^8^^,^^10^, ^11^, ^12^^,^^30^^,^^31^ As the ChAdOx1-S vaccine is based on a replication deficient adenovirus vector, the observation of off-target immunological effects is consistent with this paradigm.^3^ In mice, intranasal infection with human adenovirus elicited trained innate immunity and conferred protection against infections with unrelated bacteria.^32^ In a recent small (n = 10) study of healthy adults, monocytes stimulated with irradiated *M. tuberculosis*, TLR4 agonist lipopolysaccharides and TLR1/2 agonist Pam3cys exhibited increased secretion of IL-1β, IL-6, CXCL-1, and MIP-1α, as well as decreased TNF secretion 14 and 56 days following the first dose of ChAdOx1-S vaccination, compared to pre-vaccination responses.^20^ These changes were accompanied by increased expression of co-stimulatory molecules and glycolysis-associated enzymes, as well as the secretion of IFN-γ, IL-18, and MCP-1 in resting monocytes. This finding, of increased cytokine responses in monocytes, contrasts with our findings in whole blood wherein decreased cytokine responses to BCG and *E. coli* stimulation were observed. The difference may be due to the cellular source used in each study (monocytes compared to whole blood), particularly since multiple cytokines with altered responses to unrelated pathogens in the whole blood stimulations were associated with T cell functions and homeostasis.^33^^,^^34^

The BNT162b2 vaccine is among the first mRNA-based vaccines to be administered to humans. While it is known to induce potent adaptive immunity specific to SARS-CoV-2,^35^ the extent of its impact on immune responses and other immunoregulatory effects are yet to be fully elucidated. Three studies have shown early, transient changes in monocyte proportions one to two days following the second BNT162b2 dose,^21^^,^^36^^,^^37^ with one also demonstrating early transcriptional and transient epigenetic changes in monocytes consistent with anti-viral immune responses.^21^ Despite these early changes, one study reported no statistically significant difference in monocyte cytokine responses to *in vitro* R848 stimulation post BNT162b2 vaccination.^21^ However, with only 4 participants included, this analysis was underpowered and a pattern was observed of an early cytokine response peaking 20 days after the first dose which returned to baseline levels at 49 days (equivalent of 28 days after the second dose).^21^ In our study, we did not collect a sample after the first dose of BNT162b2 due to the recommendation for the second BNT162b2 vaccination to be 21 days after the first dose which precluded sample collection 28 days after the vaccination dose per our study design. Therefore, we were unable to assess the impact of a single BNT162b2 dose on unrelated pathogen responses. However, our finding, that BNT162b2 vaccination altered cytokine responses to the unrelated bacteria, *E. coli* and BCG, as well as to R848, 28 days after BNT162b2 vaccination, suggests that this mRNA-based vaccine might induce off-target immunological effects. Notably, after the second BNT162b2 dose, unlike ChAdOx1-S vaccination, we did not observe any off-target effects of BNT162b2 vaccination on *C. albicans* or *S. aureus* responses, although the smaller sample size for the paired BNT162b2 recipients may have limited our ability to detect changes.

Studies of the off-target immunological effects of BCG vaccination in adults have typically found increased monocyte and PBMC cytokine responses to LPS, *C. albicans*, and *S. aureus*.^8^^,^^13^^,^^30^^,^^38^^,^^39^ Similarly, ChAdOx1-S vaccination led to increased cytokine responses to *C. albicans* and *S. aureus*. These included the prototypically trained immunity induced cytokines IL-6, TNF-***α*** and IL-17.^8^^,^^13^^,^^30^ In contrast, responses to *E. coli*, from which LPS is commonly derived, and BCG were lower following ChAdOx1-S vaccination compared to before vaccination. In the case of BNT162b2, vaccination led to decreased cytokine responses to *E. coli* stimulation and had variable effects on BCG and R848 stimulation responses. These findings indicate that off-target effects of ChAdOx1-S and BNT162b2 vaccination are pathogen-dependent, consistent with previous studies showing differential effects of BCG vaccination on *in vitro* cytokine responses and bacterial growth among various pathogen species.^11^^,^^40^

Cytokines involved in broad immune functions were impacted by the ChAdOx1-S and BNT162b2 vaccinations (both enhanced and dampened depending on the vaccine and stimulus). These included cytokines involved in T cell responses, lymphocyte homeostasis, pro- and anti-inflammatory responses, growth factors and chemokines/chemoattractants. It is proposed that trained immunity, such as that induced by the BCG vaccine, provides protection against unrelated infections. However, there is also the risk that enhanced innate immune cell response contribute to diseases mediated by chronic and systemic inflammation as well as autoimmune disease.^41^ The impacts of these effects are likely situational. For example, neonatal BCG vaccination, which reduces all-cause infant mortality in high-mortality settings,^4^ has been associated with reduced cytokine responses to unrelated pathogens.^11^^,^^12^ In adults, BCG vaccination mostly increases cytokine responses to unrelated pathogens (with a notable exception of SARS-CoV-2^15^), however despite the contrasting immunological effect to that observed for neonatal BCG vaccination, BCG vaccination of adults can reduce infectious disease caused by unrelated pathogens.^42^, ^43^, ^44^ As the specific immunological effects underpinning the clinical off-target effects of vaccines are not yet fully elucidated, the clinical implications of our study findings are unclear.

Previous studies have found negative correlations between BCG-induced changes in cytokine responses typical of innate immune training (i.e., IL-10, TNF-***α***, IL-6 and IL-1β) and those typical of heterologous T cell responses (IFN-γ, IL-17, IL-22), suggesting opposing effects for trained innate and heterologous immunity.^45^ However, for both ChAdOx1-S and BNT162b2 vaccines, we generally found positive correlations within stimulus groups, although changes in prototypical T cell response cytokines were limited for BNT162b2 vaccine. These results suggest different underlying mechanisms for BCG and COVID-19 vaccine-induced off-target effects.

While BNT162b2 and ChAdOx1-S vaccines are designed to induce host cell production of the SARS-CoV-2 spike protein, previous studies have reported differing levels of SARS-CoV-2-specific antibody and T cell responses between the two vaccines.^37^^,^^46^, ^47^, ^48^, ^49^ Consistent with this, our study of healthcare workers showed that those vaccinated with BNT162b2 vaccine had greater SARS-CoV-2-specific IgG and *in vitro* cytokine responses compared to ChAdOx1-S recipients. Additionally, we observed differences in off-target effects of the two vaccines resulting in differential cytokine responses, particularly to BCG, *E. coli*, and R848 stimulation, 28 days after the second vaccine dose. An analogous study found no differences in early (two days) whole blood gene expression signatures and immune cell populations 28 days after the second dose of BNT162b2 and ChAdOx1-S vaccines,^37^ suggesting that the observed differences in immune responses are not solely driven by variations in circulating immune cell populations. The differences in specific and off-target immune responses following BNT162b2 and ChAdOx1-S vaccination despite a common antigen target (SARS-CoV-2 spike protein) suggests that vaccine formulation or the underlying platform (i.e., live replication-deficient adenovirus compared to mRNA) are influencing these responses. This is consistent with a recent re-analysis of data from COVID-19 vaccination RCTs which found reduced all-cause mortality among recipients of replication-deficient adenovirus-based COVID-19 vaccines but not mRNA-based COVID-19 vaccines.^50^ Here, the reduction was driven by a combination of reduced COVID-19, cardiovascular and non-COVID-19/non-accidental mortality suggesting a potential beneficial off-target clinical effect of adenovirus-based COVID-19 vaccines.^50^

The relationship between contemporaneous SARS-CoV-2-specific antibody and cellular responses following COVID-19 vaccines is variable and complex. Previous studies have reported different correlation patterns between antibody and T cell responses for mRNA and adenoviral COVID-19 vaccines, with some finding positive correlations and others reporting no correlations.^37^^,^^49^^,^^51^ Our study found that although both ChAdOx1-S and BNT162b2 vaccination increased SARS-CoV-2-specific IgG and *in vitro* cytokine responses, neither vaccine had strong positive correlations between these immune responses, with BNT162b2 vaccination even showing weak negative correlations. This suggests that the relationship between antibody and cellular responses to COVID-19 vaccines is non-linear and influenced by various factors. These factors might include the interplay between innate, T and B cells involved in generating and maintaining antigen-specific vaccine-induced immune memory, different functions and epitopes recognised by B and T cell populations, and dynamic changes in the immune response over time. For example, early COVID-19 vaccine-induced T cell responses correlate with later antibody responses suggesting a role for early T cell responses in generating strong humoral responses.^37^^,^^49^ Notably, the waning of circulating antibody responses to COVID-19 vaccines (including ChAdOx1-S and BNT162b2) over time and their reduced activity against SARS-CoV-2 variants of concern, despite largely consistent T cell responses and persistence of memory B cells and plasmablasts, suggest that assessment of circulating antibodies alone may not be sufficient to determine vaccine-induced immunity.^52^, ^53^, ^54^

The variation in off-target effects of vaccines and the factors that influence this variation are important questions in the field. While multiple studies report off-target effects of BCG vaccination, there are generally a sub-set of individuals who do not show any effect or even show an opposing effect.^11^^,^^13^^,^^18^^,^^39^^,^^55^ It is not yet clear what factors influence this spectrum of immunoregulatory capacity of BCG vaccine, but identifying these factors will be critical for leveraging the off-target effects for preventative or therapeutic interventions, and in the development of new vaccines. One hypothesis is that stronger vaccine-specific responses may be associated with stronger off-target effects, given the critical role of the innate immune system in the induction of antigen-specific memory. However, few studies have investigated this directly. One study that compared the effects of BCG vaccination on specific (*M. tuberculosis*-induced IFN-γ), trained immunity (*S. aureus*-induced IL-1β, IL-6, TNF-α) and heterologous lymphocyte-derived (*S. aureus*-induced IFN-γ) PBMC responses found that the specific response did not correlate with trained immunity or heterologous responses.^56^ In our study, positive correlations were observed between ChAdOx1-S-induced changes in responses to SARS-CoV-2 and *S. aureus*, both of which increased following ChAdOx1-S vaccination. However, positive correlations were also generally observed between changes in SARS-CoV-2 responses and both ChAdOx1-S- and BNT162b2-induced changes in *E. coli* response, which decreased following vaccination with either COVID-19 vaccine. This suggests that although the COVID-19 vaccines altered immune responses to unrelated pathogens, individuals who initially had a higher response of a particular cytokine to a pathogen were also the higher responders to COVID-19 vaccines. This is consistent with the strong contribution of non-modifiable factors such as genetics and age in determining interindividual variability in cytokine responses to pathogens.^57^

The strengths of our study include the use of samples from a large number of healthcare workers who received one of the two widely used COVID-19 vaccines during the same time period. As community transmission of SARS-CoV-2 was low in Australia prior to December 2021, we were able to include only SARS-CoV-2 naïve participants. Moreover, the use of weekly symptom tracking and 3-monthly anti-NCP antibody testing within the BRACE trial enabled us to identify and exclude any participants with prior SARS-CoV-2 exposure. This ensured that observed immune responses were due to the vaccine-induced immunity rather than prior infection.

The limitations of this study include that participants were not randomised to the different COVID-19 vaccines, and there were some differences in baseline demographics between the groups. During the study period, the use of BNT162b2 vaccine was preferenced by the Australian Government for adults under 50 years of age due to concerns about the rare thrombosis with thrombocytopenia syndrome reported amongst ChAdOx1-S recipients.^58^ Therefore, a difference in age between ChAdOx1-S and BNT162b2 recipients was expected. To reduce the impact of this on our findings we adjusted for participant age in the linear regression analysis. An additional limitation was the absence of a non-COVID-19-vaccinated control group. This was not feasible as it would have been unethical to delay receipt of COVID-19 vaccinations during the pandemic. Small group sizes, particularly for the paired BNT162b2 analysis may have limited the findings of our study. For analysis of effects of COVID-19 vaccines on response to BCG stimulation, recent BCG vaccination (as part of the BRACE trial) may have influenced the observed off-target effects. In the two-group analysis we were able to adjust for this by inclusion of BCG vaccination in the BRACE trial as a covariate. Another limitation is the potential effect of missing data as this study was based on a convenience sample. Participants who did not have matched samples both before and after COVID-19 vaccinations could not be included in the paired analysis. Whilst this might have led to reduced power in the analysis, it is unlikely to have resulted in any systematic bias. There was also missingness due to participants missing blood samples (i.e., provided a pre-vaccination sample but not post-vaccination sample) and those who had bloods taken outside the accepted time window post vaccination. The lack of a defined minimal clinically important difference for cytokine responses limits the interpretation of our findings.^59^ Nonetheless, whether the immunological changes observed in this proof-of-concept study translate to any clinical impact warrants further study. Finally, the assessment of immune responses at the peak of the specific adaptive immune response to COVID-19 vaccines may not fully capture the long-term effects of the vaccines. As this is an exploratory study, further research is needed to investigate the persistence and duration of the immune response and potential bystander activation effects.

Our study provides valuable insight into potential immunomodulatory effects of replication-deficient adenovirus and modified mRNA COVID-19 vaccines against a sub-set of unrelated pathogens. Further studies are needed to determine whether these effects are clinically meaningful.

## Contributors

NLM established the BCOS sub-study within the BRACE trial. NLM led and NC, KPP, EMD, LFP and SG were involved in the design of the BCOS sub-study. NC and DJL are region PIs for the BRACE trial in Victoria and South Australia respectively. DJL oversaw recruitment, sample collection and processing for the BRACE trial in South Australia (including the BCOS sub-study). NC conceived of and is the CPI of the BRACE trial. NLM oversaw sample collection, processing, and management for the BRACE trial (including the BCOS sub-study). SG and RM co-ordinated sample processing in Victoria. NES was involved in sample processing in South Australia. NLM, SG, RME, KS, RR were involved in design and/or stimulant preparation for cytokine experiments. SG and RME performed the cytokine experiments. NLM, BGB, SN were involved in design and/or performed the serology experiments. NLM, RB and EMD cleaned the data and NLM analysed the data. NLM wrote the first draft of the manuscript with input from NC, and all authors critically reviewed it.

Members of the BRACE trial consortium contributed to the design and/or operation of the BRACE trial (from which this sub-study is derived).

## Data sharing statement

Cytokine and antibody data are available at https://data.mendeley.com/drafts/y6mgxbwn6z. Additional data that support the findings of this study are available upon reasonable request. Any request for data included in this publication should be made in writing to braceresearch@mcri.edu.au.

## Declaration of interests

All authors declare no conflicts of interest. The BRACE trial consortium funding is detailed in the acknowledgements.

## Appendix A Supplementary data

Supplementary Tables S1–S7

Supplementary Fig. S1 Supplementary Fig. S1. **Participant study flow diagrams.** Diagram of participants and samples included for (a) serology and (b) whole blood stimulation analysis. ^a^excludes v1 samples collected under alternative protocol in South Australia.

Supplementary Fig. S2 Supplementary Fig. S2. **Correlations between IgG and iSARS responses following ChAdOx1-S and BNT162b2 vaccination.** (a, c, e) Heatmaps of Pearson correlation’s co-efficients and (b, d, f) representative scatter plots showing correlation between serum IgG response and iSARS stimulation induced cytokines 28 (±3 days) after (a and b) one (V1, n = 69) and (c and d) two doses (V2, n = 78) of ChAdOx1-S and (e and f) two doses (V2, n = 78) of BNT162b2 vaccination. Data were log transformed prior to Pearson’s correlation, o-values for Pearson’s correlation are presented. Heatmaps: Red indicates a negative correlation, whereas blue indicates a positive correlation. Scatter plots: blue line depicts regression line, grey indicates 95% confidence interval (CI).

Supplementary Fig. S3 Supplementary Fig. S3. **Specific and off-target in vitro immune responses 28 days after the second dose of BNT162b2 compared to ChAdOx1-S.** (a) Tukey boxplots and dot plot overlay depicting unadjusted log transformed anti-Spike and anti-RBD IgG AUC (n = 225). Grey dotted line represents the assay cut-off defined based on pre-2019 serum samples from healthy adults. (b and c) Tukey boxplots (with dot plot overlay) depicting unadjusted log transformed cytokine pg/mL secreted in response to stimulation of whole blood with (b) γ-irradiated SARS-CoV-2 (iSARS) and (c) BCG, *C. albicans*, *E. coli*, *S. aureus* and R848. Differences between vaccines determined by linear regression of log-transformed antibody and cytokine responses (adjusted only for control i.e., nil or iVero).

BRACE trial Consortium Group
