# Supplementary material for: Specific and off-target immune responses following COVID-19 vaccination with ChAdOx1-S and BNT162b2 vaccines—an exploratory sub-study of the BRACE trial
Source: eBioMedicine. 2024 Apr 24;103:105100. doi: 10.1016/j.ebiom.2024.105100 (PMC11058726; doi:10.1016/j.ebiom.2024.105100)
Supplement: Supplementary Tables S1–S7 [file mmc2.docx]

**Supplementary Table 1:** BRACE trial participants in the BCOS sub-study in Australia with V2 samples included in the unpaired analysis

|  | | Serology | |  | Whole Blood Stimulation | | |
| --- | --- | --- | --- | --- | --- | --- | --- |
|  | | ChAdOx1-S n= 124 | BNT162b2  n=101 |  | | ChAdOx1-S  n=78 | BNT162b2  n=37 |
| Age (years) at 1^st^ COVID-19 vaccination dose, median (IQR) | | | | | | | |
|  | | 51.0  (39.5 to 59.0) | 43.0  (34.0 to 53.0) |  | | 51.0  (40.0 to 59.0) | 42.0  (33.0 to 50.0) |
| Sex | | | | | | | |
| Male | | 29 (23.4%) | 26 (25.7%) |  | | 18 (23.1%) | 8 (21.6%) |
| Female | | 94 (75.8%) | 75 (74.3%) |  | | 59 (75.6%) | 29 (78.4%) |
| Declined | | 1 (0.8%) | 0 (0.0%) |  | | 1 (1.3%) | 0 (0.0%) |
| Any COVID-19 comorbidities^a^ | | | | | | | |
| Yes | | 21 (16.9%) | 24 (23.8%) |  | | 14 (17.9%) | 10 (27.0%) |
| Diabetes | 1 (4.76) | 1 (4.17%) |  | | 0 (0.0%) | 1 (10%) |  |
| Cardiovascular disease | 13 (61.9%) | 11 (45.8%) |  | | 9 (64.3%) | 4 (40.0%) |  |
| Chronic respiratory disease | 9 (42.9%) | 12 (50.0%) |  | | 6 (42.7%) | 5 (50.0%) |  |
| No | 103 (83.1%) | 76 (75.2%) |  | | 64 (82.1%) | 26 (70.3%) |  |
| missing | | 0 (0.0%) | 1 (1.0%) |  | | 0 (0.0%) | 1 (2.7%) |
| Obesity |  |  |  | |  |  |  |
| Yes | 19 (15.3%) | 11 (10.9%) |  | | 12 (15.4%) | 6 (16.2%) |  |
| No | 96 (77.4%) | 81 (80.2%) |  | | 60 (76.9%) | 29 (78.4%) |  |
| missing | 9 (7.3%) | 9 (8.9%) |  | | 6 (7.7%) | 2 (5.4%) |  |
| Smoking |  |  |  | |  |  |  |
| Yes | 5 (4.0%) | 5 (5.0%) |  | | 4 (5.1%) | 0 (0.0%) |  |
| No | 119 (96.0%) | 96 (95.0%) |  | | 74 (94.9%) | 37 (100.0%) |  |
| Occupation |  |  |  | |  |  |  |
| Allied Health | 2 (1.6%) | 2 (2.0%) |  | | 2 (2.6%) | 0 (0.0%) |  |
| Clerical/Administrative duties | 15 (12.1%) | 5 (5.0%) |  | | 8 (10.3%) | 2 (5.4%) |  |
| Doctor | 24 (19.4%) | 17 (16.8%) |  | | 14 (17.9%) | 7 (18.9%) |  |
| Nurse/Midwife | 41 (33.1%) | 55 (54.5%) |  | | 27 (34.6%) | 21 (56.8%) |  |
| Other role | 40 (32.3%) | 20 (19.8%) |  | | 27 (34.6%) | 6 (16.2%) |  |
| Patient Service Assistant | 2 (1.6%) | 1 (1.0%) |  | | 0 (0.0%) | 1 (2.7%) |  |
| Pharmacist | 0 (0.0%) | 1 (1.0%) |  | | 0 (0.0%) | 0 (0.0%) |  |
| BCG-vaccinated prior to BRACE trial | | | | | | | |
| Yes | | 74 (59.7%) | 55 (54.5%) |  | | 46 (59.0%) | 19 (51.4%) |
| No | | 50 (40.3%) | 46 (45.5%) |  | | 32 (41.0%) | 18 (48.6%) |
| BCG-vaccinated in BRACE trial | | | | | | | |
| Yes | | 90 (72.6%) | 61 (60.4%) |  | | 56 (71.8%) | 23 (62.2%) |
| No | | 34 (27.4%) | 40 (39.6%) |  | | 22 (28.2%) | 14 (37.8%) |
| Any other vaccinations between 1^st^ COVID-19 vaccine and V2 blood | | | | | | | |
| Yes | | 14 (11.3%) | 11 (10.9%) |  | | 13 (16.7%) | 4 (10.8%) |
| Days between 1^st^ and 2^nd^ COVID-19 vaccination doses, mean (SD) | | | | | | | |
|  | | 87.8 (7.6) | 24.6 (5.0) |  | | 87.4 (7.0) | 24.3 (5.5) |
| Days between second COVID-19 vaccination dose and V2 blood, mean (SD) | | | | | | | |
|  | | 28.3 (1.5) | 27.9 (1.1) |  | | 28.3 (1.4) | 28.0 (1.1) |
| Days between randomisation and first COVID-19 vaccination dose, mean (SD) | | | | | | | |
|  | | 339.2 (46.0) | 342.7 (43.7) |  | | 333.5 (54.7) | 335.1 (56.3) |
| ^a^ At BRACE trial randomisation: Diabetes (any type), cardiovascular disease (including hypertension) or chronic respiratory disease (including asthma and chronic obstructive pulmonary disease) | | | | | | | |

| **Supplementary Table 2:** Changes in cytokine responses to control (Nil/iVero) after 1 dose of ChAdOx1-S (V1-V0) in paired samples | | | | | |  | **Supplementary Table 3:** Changes in cytokine responses to control (Nil/iVero) after 2 doses of BNT162b2 (V2-V0) in paired samples | | | | | |  |
| --- | --- | --- | --- | --- | --- | --- | --- | --- | --- | --- | --- | --- | --- |
| ChAdOx1-S (V1-V0) | | | | | |  | BNT162b2 (V2-V0) | | | | | |  |
| Cytokine | Stimulant | n | Difference in median pg/mL (95% CI) 28 days after 1 dose of ChAdOx1-S (v1-v0) | Test | p-value* |  | Cytokine | Stimulant | n | Difference in median pg/mL (95% CI) 28 days after 2 doses of BNT162b2 (v2-v0) | Test | p-value* | |
| CTACK | Nil | 58 | 24.03 (11.44 to 46.95) | WSR | 0.00048 |  | CTACK | Nil | 19 | 12.69 (-16.04 to 41.87) | WSR | 0.46 | |
| EOTAXIN | Nil | 58 | 4.98 (-1.21 to 10.63) | WSR | 0.02 |  | EOTAXIN | Nil | 19 | 0.21 (-10.00 to 10.93) | WSR | 0.75 | |
| FGF-basic | Nil | 58 | 7.96 (0.00 to 18.28) | ST | 0.033 |  | FGF-basic | Nil | 19 | 0.00 (0.00 to 21.10) | WSR | 0.34 | |
| G-CSF | Nil | 58 | 105.34 (0.49 to 215.53) | ST | 0.063 |  | G-CSF | Nil | 19 | -157.47 (-325.00 to 102.53) | ST | 0.24 | |
| GM-CSF | Nil | 58 | 0.00 (0.00 to 0.84) | ST | 0.020 |  | GM-CSF | Nil | 19 | 0.00 (0.00 to 0.81) | ST | 0.51 | |
| GRO-𝛼 | Nil | 58 | 0.00 (-40.50 to 233.05) | ST | 1.0 |  | GRO-𝛼 | Nil | 19 | 106.90 (-206.84 to 553.28) | WSR | 0.23 | |
| HGF | Nil | 58 | 41.25 (1.20 to 62.10) | ST | 0.0078 |  | HGF | Nil | 19 | 12.25 (-28.92 to 57.11) | ST | 0.81 | |
| IFN-𝛼2 | Nil | 58 | 0.00 (0.00 to 0.00) | ST | 0.061 |  | IFN-𝛼2 | Nil | 19 | 0.00 (-0.33 to 1.87) | ST | 1.0 | |
| IFN-𝛾 | Nil | 58 | 5.37 (1.05 to 17.01) | ST | 0.0012 |  | IFN-𝛾 | Nil | 19 | 3.34 (-11.30 to 47.40) | ST | 0.48 | |
| IL-1𝛼 | Nil | 58 | 1.34 (0.00 to 8.33) | ST | 0.012 |  | IL-1𝛼 | Nil | 19 | 0.00 (-12.07 to 16.22) | WSR | 0.56 | |
| IL-1𝛽 | Nil | 58 | 0.84 (0.00 to 1.49) | ST | 0.16 |  | IL-1𝛽 | Nil | 19 | -0.90 (-4.09 to 0.24) | ST | 0.21 | |
| IL-1Ra | Nil | 58 | 225.09 (-57.60 to 549.27) | WSR | 0.15 |  | IL-1Ra | Nil | 19 | -353.50 (-863.37 to 301.92) | WSR | 0.30 | |
| IL-2 | Nil | 58 | 0.00 (0.00 to 0.00) | ST | 0.63 |  | IL-2 | Nil | 19 | 0.00 (-5.77 to 3.24) | WSR | 0.59 | |
| IL-2R𝛼 | Nil | 58 | 3.16 (0.00 to 10.56) | ST | 0.076 |  | IL-2R𝛼 | Nil | 19 | 9.95 (-3.98 to 15.39) | WSR | 0.15 | |
| IL-3 | Nil | 58 | 0.00 (0.00 to 0.00) | ST | 1.0 |  | IL-3 | Nil | 19 | 0.00 (0.00 to 0.00) | ST | 1.0 | |
| IL-4 | Nil | 58 | 0.00 (0.00 to 1.32) | ST | 0.038 |  | IL-4 | Nil | 19 | 1.13 (-0.11 to 2.44) | WSR | 0.068 | |
| IL-5 | Nil | 58 | 0.00 (0.00 to 0.00) | WSR | 0.64 |  | IL-5 | Nil | 19 | 0.00 (-45.89 to 21.64) | ST | 0.58 | |
| IL-6 | Nil | 58 | 0.00 (-1.04 to 1.53) | ST | 0.89 |  | IL-6 | Nil | 19 | -3.27 (-9.59 to -1.78) | ST | 0.019 | |
| IL-7 | Nil | 58 | 0.00 (0.00 to 0.00) | ST | 0.29 |  | IL-7 | Nil | 19 | 0.00 (0.00 to 3.89) | ST | 0.55 | |
| IL-8 | Nil | 58 | 91.32 (24.49 to 177.80) | ST | 0.0022 |  | IL-8 | Nil | 19 | 74.67 (-21.03 to 240.02) | ST | 0.65 | |
| IL-9 | Nil | 58 | 26.43 (-5.34 to 49.51) | ST | 0.11 |  | IL-9 | Nil | 19 | 44.28 (-20.03 to 87.50) | WSR | 0.14 | |
| IL-10 | Nil | 58 | 0.00 (0.00 to 0.00) | ST | 0.87 |  | IL-10 | Nil | 19 | 0.00 (-5.99 to 3.79) | WSR | 0.84 | |
| IL-12p70 | Nil | 58 | 0.00 (0.00 to 0.00) | ST | 0.84 |  | IL-12p70 | Nil | 19 | 0.00 (0.00 to 0.00) | ST | 1.0 | |
| IL-12p40 | Nil | 58 | 0.00 (0.00 to 0.00) | ST | 0.076 |  | IL-12p40 | Nil | 19 | 0.00 (0.00 to 0.00) | ST | 1.0 | |
| IL-13 | Nil | 58 | 0.00 (0.00 to 0.00) | ST | 0.85 |  | IL-13 | Nil | 19 | 0.00 (-0.11 to 0.25) | ST | 1.0 | |
| IL-15 | Nil | 58 | 0.00 (0.00 to 0.00) | ST | 0.87 |  | IL-15 | Nil | 19 | 29.19 (-54.61 to 105.62) | WSR | 0.59 | |
| IL-16 | Nil | 58 | 9.77 (-5.75 to 17.80) | ST | 0.27 |  | IL-16 | Nil | 19 | 6.32 (-13.18 to 24.28) | ST | 1.0 | |
| IL-17 | Nil | 58 | 0.00 (0.00 to 2.68) | ST | 0.12 |  | IL-17 | Nil | 19 | 0.00 (-0.35 to 5.13) | WSR | 0.17 | |
| IL-18 | Nil | 58 | 3.16 (0.00 to 8.95) | ST | 0.065 |  | IL-18 | Nil | 19 | 10.16 (-2.63 to 22.59) | WSR | 0.03 | |
| IP-10 | Nil | 58 | 3.35 (-107.37 to 222.83) | WSR | 0.39 |  | IP-10 | Nil | 19 | -21.76 (-316.27 to 201.31) | ST | 1.0 | |
| LIF | Nil | 58 | 0.00 (0.00 to 8.21) | ST | 0.26 |  | LIF | Nil | 19 | 0.00 (-17.70 to 19.01) | WSR | 0.79 | |
| MCP-1 | Nil | 57 | 85.48 (11.80 to 312.67) | ST | 0.0046 |  | MCP-1 | Nil | 19 | 144.95 (-12.04 to 679.92) | ST | 0.17 | |
| MCP-3 | Nil | 58 | 9.35 (1.68 to 19.65) | ST | 0.00094 |  | MCP-3 | Nil | 19 | 2.52 (-1.63 to 47.39) | ST | 0.21 | |
| M-CSF | Nil | 58 | 2.74 (0.00 to 5.57) | ST | 0.13 |  | M-CSF | Nil | 19 | 1.46 (-2.58 to 4.98) | WSR | 0.48 | |
| MIF | Nil | 58 | 148.42 (-89.80 to 298.05) | ST | 0.24 |  | MIF | Nil | 19 | 73.72 (-77.60 to 289.90) | ST | 1.0 | |
| MIG | Nil | 58 | 20.67 (-15.54 to 66.48) | ST | 0.42 |  | MIG | Nil | 19 | 12.50 (-31.51 to 45.81) | ST | 0.65 | |
| MIP-1𝛼 | Nil | 58 | 1.03 (-1.26 to 3.89) | ST | 0.42 |  | MIP-1𝛼 | Nil | 19 | -2.97 (-9.07 to 0.89) | ST | 0.17 | |
| MIP-1𝛽 | Nil | 58 | 6.06 (-27.11 to 109.36) | WSR | 0.34 |  | MIP-1𝛽 | Nil | 19 | -89.48 (-307.42 to 86.73) | ST | 0.36 | |
| 𝛽-NGF | Nil | 58 | 0.65 (0.00 to 3.40) | WSR | 0.44 |  | 𝛽-NGF | Nil | 19 | 0.00 (-3.99 to 0.26) | WSR | 0.36 | |
| PDGF-BB | Nil | 58 | 121.55 (11.78 to 197.61) | ST | 0.010 |  | PDGF-BB | Nil | 19 | 50.41 (-30.45 to 179.60) | WSR | 0.056 | |
| RANTES | Nil | 58 | 36.74 (-90.52 to 258.96) | ST | 0.51 |  | RANTES | Nil | 19 | 213.39 (-178.92 to 534.41) | WSR | 0.14 | |
| SCF | Nil | 58 | 11.25 (4.40 to 16.58) | ST | <0.0001 |  | SCF | Nil | 19 | 21.15 (1.37 to 24.19) | WSR | 0.028 | |
| SCGF-𝛽 | Nil | 58 | 2964.48 (81.94 to 4884.25) | ST | 0.048 |  | SCGF-𝛽 | Nil | 19 | -1630.49 (-4868.42 to 4002.52) | WSR | 0.72 | |
| SDF-1𝛼 | Nil | 58 | 126.82 (1.00 to 224.54) | WSR | 0.0023 |  | SDF-1𝛼 | Nil | 19 | 10.90 (-75.93 to 173.73) | WSR | 0.3 | |
| TNF-𝛼 | Nil | 58 | 7.27 (0.00 to 17.72) | ST | 0.029 |  | TNF-𝛼 | Nil | 19 | 1.64 (-5.56 to 27.36) | ST | 0.45 | |
| TNF-𝛽 | Nil | 58 | 33.57 (-33.63 to 82.27) | ST | 0.24 |  | TNF-𝛽 | Nil | 19 | 49.77 (-33.25 to 127.47) | WSR | 0.12 | |
| TRAIL | Nil | 58 | 7.04 (-0.50 to 15.65) | ST | 0.27 |  | TRAIL | Nil | 19 | 1.32 (-4.39 to 11.44) | ST | 1.0 | |
| VEGF | Nil | 58 | 0.00 (0.00 to 61.82) | WSR | 0.35 |  | VEGF | Nil | 19 | 0.00 (-104.84 to 106.81) | WSR | 0.95 | |
| CTACK | iVero | 55 | 10.98 (-8.42 to 29.35) | WSR | 0.062 |  | CTACK | iVero | 18 | 2.61 (-31.50 to 45.33) | WSR | 0.63 | |
| EOTAXIN | iVero | 55 | 3.33 (-1.90 to 9.44) | WSR | 0.15 |  | EOTAXIN | iVero | 18 | 0.40 (-7.25 to 11.69) | WSR | 0.57 | |
| FGF-basic | iVero | 55 | 6.55 (0.00 to 14.84) | ST | 0.049 |  | FGF-basic | iVero | 18 | 9.28 (0.00 to 42.84) | ST | 0.27 | |
| G-CSF | iVero | 55 | 96.33 (24.17 to 151.55) | ST | 0.013 |  | G-CSF | iVero | 18 | -79.91 (-268.19 to 133.76) | ST | 0.48 | |
| GM-CSF | iVero | 55 | 0.00 (0.00 to 0.00) | ST | 0.73 |  | GM-CSF | iVero | 18 | 0.00 (0.00 to 2.00) | WSR | 0.74 | |
| GRO-𝛼 | iVero | 55 | 151.07 (0.00 to 365.80) | ST | 0.049 |  | GRO-𝛼 | iVero | 18 | 360.05 (193.37 to 1109.22) | ST | 0.0075 | |
| HGF | iVero | 55 | 0.00 (-25.73 to 19.28) | ST | 0.88 |  | HGF | iVero | 18 | 0.00 (-38.70 to 40.92) | WSR | 0.98 | |
| IFN-𝛼2 | iVero | 55 | 0.00 (0.00 to 6.46) | ST | 0.011 |  | IFN-𝛼2 | iVero | 18 | 0.00 (0.00 to 6.34) | ST | 0.55 | |
| IFN-𝛾 | iVero | 55 | 15.35 (10.04 to 40.04) | ST | <0.0001 |  | IFN-𝛾 | iVero | 18 | 9.65 (-7.33 to 80.97) | ST | 0.48 | |
| IL-1𝛼 | iVero | 55 | 0.00 (0.00 to 0.00) | ST | 0.72 |  | IL-1𝛼 | iVero | 18 | 0.00 (-2.37 to 6.60) | WSR | 0.66 | |
| IL-1𝛽 | iVero | 55 | 0.51 (-0.14 to 1.41) | ST | 0.25 |  | IL-1𝛽 | iVero | 18 | -0.19 (-2.09 to 2.15) | ST | 0.61 | |
| IL-1Ra | iVero | 55 | 166.49 (-29.35 to 387.59) | ST | 0.076 |  | IL-1Ra | iVero | 18 | -161.35 (-845.59 to 464.92) | WSR | 0.33 | |
| IL-2 | iVero | 55 | 0.00 (0.00 to 2.22) | ST | 0.26 |  | IL-2 | iVero | 18 | 0.00 (-1.23 to 4.04) | WSR | 0.48 | |
| IL-2R𝛼 | iVero | 55 | 2.97 (0.00 to 8.00) | WSR | 0.079 |  | IL-2R𝛼 | iVero | 18 | 1.86 (-3.82 to 21.63) | WSR | 0.32 | |
| IL-3 | iVero | 55 | 0.00 (0.00 to 0.00) | ST | 1.0 |  | IL-3 | iVero | 18 | 0.00 (0.00 to 0.00) | ST | 0.25 | |
| IL-4 | iVero | 55 | 0.00 (0.00 to 1.70) | ST | 0.014 |  | IL-4 | iVero | 18 | 0.00 (-1.55 to 3.24) | WSR | 0.60 | |
| IL-5 | iVero | 55 | 0.00 (0.00 to 7.18) | ST | 1.0 |  | IL-5 | iVero | 18 | 0.00 (-29.43 to 123.45) | ST | 1.0 | |
| IL-6 | iVero | 55 | 14.12 (2.17 to 33.19) | ST | 0.030 |  | IL-6 | iVero | 18 | -3.99 (-27.86 to 44.61) | WSR | 0.91 | |
| IL-7 | iVero | 55 | 0.00 (0.00 to 0.00) | ST | 0.33 |  | IL-7 | iVero | 18 | 0.00 (0.00 to 0.00) | ST | 0.69 | |
| IL-8 | iVero | 55 | 194.18 (93.72 to 312.25) | ST | 0.00036 |  | IL-8 | iVero | 18 | -11.84 (-250.34 to 665.78) | WSR | 0.71 | |
| IL-9 | iVero | 55 | 22.63 (-6.98 to 83.72) | WSR | 0.10 |  | IL-9 | iVero | 18 | 45.26 (28.75 to 211.97) | WSR | 0.043 | |
| IL-10 | iVero | 55 | 0.00 (0.00 to 3.23) | ST | 0.081 |  | IL-10 | iVero | 18 | 3.02 (-0.70 to 6.90) | WSR | 0.12 | |
| IL-12p70 | iVero | 55 | 0.00 (0.00 to 0.00) | ST | 0.66 |  | IL-12p70 | iVero | 18 | 0.00 (0.00 to 2.64) | WSR | 0.19 | |
| IL-12p40 | iVero | 55 | 0.00 (0.00 to 0.00) | ST | 0.023 |  | IL-12p40 | iVero | 18 | 0.00 (0.00 to 0.00) | ST | 1.0 | |
| IL-13 | iVero | 55 | 0.00 (0.00 to 0.00) | ST | 0.85 |  | IL-13 | iVero | 18 | 0.00 (0.00 to 0.73) | ST | 0.039 | |
| IL-15 | iVero | 55 | 0.00 (0.00 to 0.00) | ST | 0.61 |  | IL-15 | iVero | 18 | 53.06 (-71.82 to 192.54) | WSR | 0.32 | |
| IL-16 | iVero | 55 | 0.00 (-8.17 to 11.27) | ST | 1.0 |  | IL-16 | iVero | 18 | 1.05 (-6.13 to 11.98) | ST | 0.80 | |
| IL-17 | iVero | 55 | 0.00 (0.00 to 0.00) | ST | 0.73 |  | IL-17 | iVero | 18 | 0.00 (-0.54 to 2.83) | WSR | 0.56 | |
| IL-18 | iVero | 55 | 2.15 (-1.72 to 10.63) | ST | 0.26 |  | IL-18 | iVero | 18 | 7.52 (-2.28 to 23.47) | WSR | 0.028 | |
| IP-10 | iVero | 55 | 31.82 (-47.15 to 108.47) | ST | 0.18 |  | IP-10 | iVero | 18 | 13.32 (-22.97 to 202.04) | ST | 0.24 | |
| LIF | iVero | 55 | 0.00 (-2.18 to 13.21) | ST | 0.55 |  | LIF | iVero | 18 | 0.00 (-15.35 to 34.80) | WSR | 0.35 | |
| MCP-1 | iVero | 55 | 488.72 (157.59 to 1210.94) | ST | <0.0001 |  | MCP-1 | iVero | 18 | 154.97 (-177.40 to 1728.59) | WSR | 0.17 | |
| MCP-3 | iVero | 55 | 28.08 (14.66 to 75.41) | ST | <0.0001 |  | MCP-3 | iVero | 18 | 18.44 (-3.09 to 102.37) | ST | 0.14 | |
| M-CSF | iVero | 55 | 1.26 (-1.44 to 4.96) | WSR | 0.40 |  | M-CSF | iVero | 18 | 3.50 (-2.81 to 12.40) | ST | 0.81 | |
| MIF | iVero | 55 | 93.81 (-155.14 to 205.64) | ST | 0.50 |  | MIF | iVero | 18 | -74.74 (-293.91 to 83.70) | WSR | 0.40 | |
| MIG | iVero | 55 | -3.40 (-30.42 to 39.42) | ST | 0.89 |  | MIG | iVero | 18 | 19.35 (-29.42 to 90.78) | ST | 0.81 | |
| MIP-1𝛼 | iVero | 55 | 0.46 (-0.06 to 1.03) | ST | 0.25 |  | MIP-1𝛼 | iVero | 18 | -1.40 (-6.15 to 0.78) | ST | 0.33 | |
| MIP-1𝛽 | iVero | 55 | 24.78 (-26.87 to 58.58) | WSR | 0.18 |  | MIP-1𝛽 | iVero | 18 | -46.53 (-148.97 to 33.71) | WSR | 0.25 | |
| 𝛽-NGF | iVero | 55 | 0.00 (-1.57 to 1.96) | ST | 0.47 |  | 𝛽-NGF | iVero | 18 | 4.06 (0.32 to 12.97) | WSR | 0.021 | |
| PDGF-BB | iVero | 55 | -29.81 (-88.02 to 104.42) | ST | 0.78 |  | PDGF-BB | iVero | 18 | 100.04 (60.34 to 258.44) | WSR | 0.021 | |
| RANTES | iVero | 55 | 161.06 (-260.18 to 417.36) | WSR | 0.63 |  | RANTES | iVero | 18 | 294.79 (28.08 to 1145.00) | WSR | 0.085 | |
| SCF | iVero | 55 | 10.39 (0.00 to 19.41) | ST | 0.024 |  | SCF | iVero | 18 | 13.81 (4.91 to 31.30) | WSR | 0.0074 | |
| SCGF-𝛽 | iVero | 55 | -2901.14 (-4756.82 to 2958.73) | WSR | 0.80 |  | SCGF-𝛽 | iVero | 18 | -836.73 (-2938.79 to 11370.92) | WSR | 0.41 | |
| SDF-1𝛼 | iVero | 55 | 26.48 (-51.35 to 126.35) | WSR | 0.30 |  | SDF-1𝛼 | iVero | 18 | 97.24 (-13.69 to 205.21) | WSR | 0.020 | |
| TNF-𝛼 | iVero | 55 | 10.23 (0.00 to 17.67) | ST | 0.033 |  | TNF-𝛼 | iVero | 18 | 2.66 (-7.52 to 47.16) | WSR | 0.28 | |
| TNF-𝛽 | iVero | 55 | 58.89 (-28.78 to 117.33) | WSR | 0.18 |  | TNF-𝛽 | iVero | 18 | 85.61 (26.80 to 340.02) | WSR | 0.012 | |
| TRAIL | iVero | 55 | 9.29 (3.68 to 20.18) | ST | 0.030 |  | TRAIL | iVero | 18 | 5.27 (-1.03 to 45.53) | ST | 0.24 | |
| VEGF | iVero | 55 | 73.41 (-63.19 to 197.46) | WSR | 0.32 |  | VEGF | iVero | 18 | 114.29 (-56.43 to 274.98) | WSR | 0.048 | |

* Determined by test indicated in ‘Test’ column

ST, Sign test; WSR, Wilcoxon signed-rank test

| **Supplementary Table 4a**: Changes in cytokine stimulation effect (stimulant-control) after 1 dose of ChAdOx1-S (V1-V0) in paired samples | | | | | | |  | | **Supplementary Table 5a**: Changes in cytokine stimulation effect (stimulant-control) after 2 doses of BNT162b2 (V2-V0) in paired samples | | | | | |  |
| --- | --- | --- | --- | --- | --- | --- | --- | --- | --- | --- | --- | --- | --- | --- | --- |
| ChAdOx1-S (V1-V0) | | | | | | |  | | BNT162b2 (V2-V0) | | | | | |  |
| Cytokine | Stimulant | n | Difference in median stimulation effect (95% CI) 28 days after 1 dose of ChAdOx1-S (v1-v0) | Test | p-value* |  | | Cytokine | | Stimulant | n | Difference in median stimulation effect (95% CI) 28 days after 2 doses of BNT162b2 (v2-v0) | Test | p-value* | |
| CTACK | iSARS | 55 | 8.73 (-6.12 to 18.22) | WSR | 0.22 |  | | CTACK | | iSARS | 18 | 50.96 (20.72 to 70.01) | ST | 0.0075 | |
| EOTAXIN | iSARS | 55 | -6.29 (-10.11 to -2.77) | WSR | 0.0032 |  | | EOTAXIN | | iSARS | 18 | 1.05 (-3.93 to 4.24) | WSR | 0.91 | |
| FGF-basic | iSARS | 55 | 45.13 (36.53 to 59.73) | WSR | <0.0001 |  | | FGF-basic | | iSARS | 18 | 71.37 (48.35 to 95.84) | WSR | 0.0014 | |
| G-CSF | iSARS | 55 | 3210.23 (2545.45 to 4542.49) | ST | <0.0001 |  | | G-CSF | | iSARS | 18 | 6357.75 (4582.83 to 10481.96) | WSR | 0.00039 | |
| GM-CSF | iSARS | 55 | 5.87 (3.04 to 8.62) | WSR | <0.0001 |  | | GM-CSF | | iSARS | 18 | 11.58 (7.06 to 16.79) | ST | 0.00014 | |
| GRO-𝛼 | iSARS | 55 | 354.29 (211.45 to 511.07) | WSR | <0.0001 |  | | GRO-𝛼 | | iSARS | 18 | 1036.01 (306.09 to 1350.45) | ST | 0.0013 | |
| HGF | iSARS | 55 | 112.59 (93.38 to 156.22) | ST | <0.0001 |  | | HGF | | iSARS | 18 | 256.98 (193.03 to 394.78) | WSR | 0.00021 | |
| IFN-𝛼2 | iSARS | 55 | -25.10 (-121.67 to 29.21) | WSR | 0.059 |  | | IFN-𝛼2 | | iSARS | 18 | -10.44 (-190.60 to 28.23) | ST | 1.0 | |
| IFN-𝛾 | iSARS | 55 | 71.66 (33.11 to 98.94) | ST | <0.0001 |  | | IFN-𝛾 | | iSARS | 18 | 137.17 (65.71 to 345.17) | ST | 0.00014 | |
| IL-1𝛼 | iSARS | 55 | 29.29 (19.21 to 41.22) | ST | <0.0001 |  | | IL-1𝛼 | | iSARS | 18 | 70.68 (43.58 to 82.70) | WSR | 0.00020 | |
| IL-1𝛽 | iSARS | 55 | 16.32 (10.13 to 20.01) | ST | <0.0001 |  | | IL-1𝛽 | | iSARS | 18 | 25.72 (16.49 to 56.66) | WSR | 0.0029 | |
| IL-1Ra | iSARS | 55 | 1987.18 (952.43 to 2698.59) | WSR | 0.00034 |  | | IL-1Ra | | iSARS | 18 | 5685.68 (3161.13 to 7411.08) | WSR | 0.00020 | |
| IL-2 | iSARS | 55 | 48.54 (32.43 to 64.94) | ST | <0.0001 |  | | IL-2 | | iSARS | 18 | 64.01 (43.77 to 183.37) | ST | <0.0001 | |
| IL-2R𝛼 | iSARS | 55 | 26.75 (18.20 to 34.34) | ST | <0.0001 |  | | IL-2R𝛼 | | iSARS | 18 | 58.83 (45.83 to 91.61) | WSR | 0.00023 | |
| IL-3 | iSARS | 55 | 0.00 (0.00 to 1.48) | ST | <0.0001 |  | | IL-3 | | iSARS | 18 | 0.15 (0.00 to 3.51) | WSR | 0.021 | |
| IL-4 | iSARS | 55 | 8.90 (7.00 to 12.35) | ST | <0.0001 |  | | IL-4 | | iSARS | 18 | 14.27 (11.96 to 21.57) | WSR | 0.00020 | |
| IL-5 | iSARS | 55 | 240.47 (184.38 to 295.92) | WSR | <0.0001 |  | | IL-5 | | iSARS | 18 | 465.73 (171.03 to 670.75) | ST | 0.013 | |
| IL-6 | iSARS | 55 | 89.66 (34.69 to 181.30) | ST | <0.0001 |  | | IL-6 | | iSARS | 18 | 359.89 (111.77 to 674.63) | ST | 0.0013 | |
| IL-7 | iSARS | 55 | 0.00 (0.00 to 0.00) | ST | 0.84 |  | | IL-7 | | iSARS | 18 | 0.00 (0.00 to 9.51) | ST | 1.0 | |
| IL-8 | iSARS | 55 | -14.82 (-47.99 to 77.64) | WSR | 0.61 |  | | IL-8 | | iSARS | 18 | 382.75 (2.35 to 914.25) | ST | 0.096 | |
| IL-9 | iSARS | 55 | 21.48 (-1.74 to 53.81) | WSR | 0.15 |  | | IL-9 | | iSARS | 18 | 96.50 (27.21 to 164.75) | ST | 0.0075 | |
| IL-10 | iSARS | 55 | 28.45 (20.09 to 35.25) | ST | <0.0001 |  | | IL-10 | | iSARS | 18 | 57.72 (32.58 to 99.38) | WSR | 0.00023 | |
| IL-12p70 | iSARS | 55 | 0.00 (0.00 to 2.32) | ST | 0.011 |  | | IL-12p70 | | iSARS | 18 | 10.24 (0.00 to 18.46) | ST | 0.035 | |
| IL-12p40 | iSARS | 55 | 86.67 (65.84 to 115.34) | ST | <0.0001 |  | | IL-12p40 | | iSARS | 18 | 154.77 (45.21 to 271.26) | WSR | 0.00041 | |
| IL-13 | iSARS | 55 | 0.79 (0.00 to 1.75) | WSR | 0.00059 |  | | IL-13 | | iSARS | 18 | 4.18 (1.46 to 7.26) | ST | 0.00014 | |
| IL-15 | iSARS | 55 | 99.55 (15.57 to 226.79) | WSR | 0.00069 |  | | IL-15 | | iSARS | 18 | 287.55 (163.24 to 467.37) | WSR | 0.0050 | |
| IL-16 | iSARS | 55 | 63.71 (40.43 to 79.95) | ST | <0.0001 |  | | IL-16 | | iSARS | 18 | 79.53 (55.66 to 119.31) | ST | <0.0001 | |
| IL-17 | iSARS | 55 | 14.50 (9.07 to 20.90) | ST | <0.0001 |  | | IL-17 | | iSARS | 18 | 29.81 (18.00 to 48.57) | WSR | 0.00023 | |
| IL-18 | iSARS | 55 | 1.41 (-2.37 to 3.65) | WSR | 0.49 |  | | IL-18 | | iSARS | 18 | 13.81 (-3.52 to 31.10) | WSR | 0.020 | |
| IP-10 | iSARS | 55 | 4325.74 (-6149.15 to 7025.71) | ST | 0.42 |  | | IP-10 | | iSARS | 18 | 1431.49 (-9758.45 to 9471.79) | WSR | 0.95 | |
| LIF | iSARS | 55 | 72.02 (51.80 to 98.96) | ST | <0.0001 |  | | LIF | | iSARS | 18 | 125.04 (84.77 to 146.95) | ST | <0.0001 | |
| MCP-1 | iSARS | 52 | 3955.92 (2851.80 to 5762.58) | ST | <0.0001 |  | | MCP-1 | | iSARS | 15 | 7947.35 (4312.28 to 46894.98) | ST | <0.0001 | |
| MCP-3 | iSARS | 55 | 713.23 (479.34 to 1035.47) | ST | <0.0001 |  | | MCP-3 | | iSARS | 18 | 1243.42 (916.67 to 1863.64) | WSR | 0.00020 | |
| M-CSF | iSARS | 55 | 5.51 (1.98 to 9.93) | WSR | 0.00028 |  | | M-CSF | | iSARS | 18 | 15.46 (0.68 to 22.59) | WSR | 0.0033 | |
| MIF | iSARS | 55 | 129.94 (15.55 to 357.69) | WSR | 0.087 |  | | MIF | | iSARS | 18 | 306.99 (90.43 to 507.93) | WSR | 0.0018 | |
| MIG | iSARS | 55 | 68.41 (16.86 to 135.70) | ST | 0.030 |  | | MIG | | iSARS | 18 | 67.07 (2.06 to 165.97) | WSR | 0.022 | |
| MIP-1𝛼 | iSARS | 55 | 111.87 (78.14 to 164.69) | ST | <0.0001 |  | | MIP-1𝛼 | | iSARS | 18 | 232.06 (120.14 to 530.34) | ST | 0.0013 | |
| MIP-1𝛽 | iSARS | 55 | 1736.25 (1394.35 to 2079.97) | ST | <0.0001 |  | | MIP-1𝛽 | | iSARS | 18 | 2328.50 (1756.24 to 3668.14) | WSR | 0.00028 | |
| 𝛽-NGF | iSARS | 55 | 5.96 (2.15 to 9.51) | WSR | 0.00033 |  | | 𝛽-NGF | | iSARS | 18 | 13.82 (4.34 to 23.29) | WSR | 0.00093 | |
| PDGF-BB | iSARS | 55 | 43.68 (-29.34 to 101.53) | WSR | 0.11 |  | | PDGF-BB | | iSARS | 18 | 86.13 (-54.02 to 188.93) | WSR | 0.078 | |
| RANTES | iSARS | 55 | 47.51 (-121.17 to 301.15) | WSR | 0.62 |  | | RANTES | | iSARS | 18 | 193.55 (-237.28 to 792.48) | WSR | 0.25 | |
| SCF | iSARS | 55 | 114.32 (77.12 to 137.49) | ST | <0.0001 |  | | SCF | | iSARS | 18 | 178.87 (124.54 to 276.25) | WSR | 0.00020 | |
| SCGF-𝛽 | iSARS | 55 | 1961.22 (-2741.47 to 3786.45) | WSR | 0.74 |  | | SCGF-𝛽 | | iSARS | 18 | 2436.10 (-5100.80 to 8474.60) | WSR | 0.74 | |
| SDF-1𝛼 | iSARS | 55 | 14.11 (-40.02 to 109.06) | WSR | 0.19 |  | | SDF-1𝛼 | | iSARS | 18 | 21.95 (-48.80 to 120.17) | WSR | 0.37 | |
| TNF-𝛼 | iSARS | 55 | 151.16 (114.96 to 198.66) | ST | <0.0001 |  | | TNF-𝛼 | | iSARS | 18 | 254.21 (206.55 to 374.77) | ST | <0.0001 | |
| TNF-𝛽 | iSARS | 55 | 30.67 (-23.00 to 82.40) | WSR | 0.25 |  | | TNF-𝛽 | | iSARS | 18 | 156.56 (-44.15 to 214.06) | ST | 0.24 | |
| TRAIL | iSARS | 55 | 85.77 (66.34 to 106.88) | ST | <0.0001 |  | | TRAIL | | iSARS | 18 | 148.31 (103.08 to 265.84) | WSR | 0.00020 | |
| VEGF | iSARS | 55 | 153.05 (25.99 to 343.01) | WSR | 0.024 |  | | VEGF | | iSARS | 18 | 340.92 (114.49 to 611.06) | WSR | 0.0065 | |
| CTACK | BCG | 57 | -30.22 (-69.43 to 32.41) | ST | 0.43 |  | | CTACK | | BCG | 19 | 9.06 (-148.95 to 121.01) | WSR | 0.97 | |
| EOTAXIN | BCG | 57 | 0.40 (-1.73 to 3.72) | ST | 1.0 |  | | EOTAXIN | | BCG | 19 | 1.72 (-0.97 to 10.88) | WSR | 0.12 | |
| FGF-basic | BCG | 57 | -16.95 (-25.90 to -1.45) | ST | 0.033 |  | | FGF-basic | | BCG | 19 | -1.97 (-35.64 to 20.74) | WSR | 0.72 | |
| G-CSF | BCG | 57 | -616.07 (-3989.44 to 817.49) | WSR | 0.12 |  | | G-CSF | | BCG | 19 | 986.73 (-3703.98 to 4386.85) | WSR | 0.72 | |
| GM-CSF | BCG | 57 | -5.86 (-11.80 to 4.20) | WSR | 0.14 |  | | GM-CSF | | BCG | 19 | 1.83 (-8.61 to 14.68) | ST | 1.0 | |
| GRO-𝛼 | BCG | 57 | -1557.36 (-3907.37 to 592.63) | WSR | 0.098 |  | | GRO-𝛼 | | BCG | 19 | -2091.72 (-4414.20 to 532.44) | WSR | 0.13 | |
| HGF | BCG | 57 | -58.93 (-158.18 to -22.30) | ST | 0.016 |  | | HGF | | BCG | 19 | 59.85 (-111.94 to 117.83) | WSR | 0.52 | |
| IFN-𝛼2 | BCG | 57 | -7.07 (-10.67 to -1.41) | ST | 0.022 |  | | IFN-𝛼2 | | BCG | 19 | 0.00 (-11.96 to 5.31) | ST | 1.0 | |
| IFN-𝛾 | BCG | 57 | -50.47 (-70.75 to -8.83) | ST | 0.016 |  | | IFN-𝛾 | | BCG | 19 | -175.79 (-424.50 to -17.75) | WSR | 0.0079 | |
| IL-1𝛼 | BCG | 57 | 79.10 (-15.64 to 162.21) | ST | 0.11 |  | | IL-1𝛼 | | BCG | 19 | 87.49 (-28.58 to 240.30) | WSR | 0.033 | |
| IL-1𝛽 | BCG | 57 | 418.24 (-420.54 to 913.76) | WSR | 0.38 |  | | IL-1𝛽 | | BCG | 19 | 1298.04 (-1016.38 to 3029.80) | WSR | 0.16 | |
| IL-1Ra | BCG | 57 | -468.86 (-1360.36 to 1436.27) | WSR | 0.45 |  | | IL-1Ra | | BCG | 19 | 716.46 (-1237.97 to 4354.80) | WSR | 0.077 | |
| IL-2 | BCG | 57 | -2.53 (-28.57 to 18.00) | ST | 0.79 |  | | IL-2 | | BCG | 19 | -5.26 (-24.93 to 12.61) | ST | 0.36 | |
| IL-2R𝛼 | BCG | 57 | -19.36 (-34.85 to -5.78) | ST | 0.00046 |  | | IL-2R𝛼 | | BCG | 19 | -22.95 (-33.56 to -0.72) | WSR | 0.15 | |
| IL-3 | BCG | 57 | 0.00 (-1.58 to 0.49) | ST | 0.78 |  | | IL-3 | | BCG | 19 | 0.71 (-1.81 to 2.48) | ST | 0.81 | |
| IL-4 | BCG | 57 | -2.24 (-3.32 to -1.18) | ST | 0.0075 |  | | IL-4 | | BCG | 19 | -2.82 (-4.31 to 1.87) | ST | 0.24 | |
| IL-5 | BCG | 57 | -171.67 (-299.90 to 98.27) | ST | 0.18 |  | | IL-5 | | BCG | 19 | 134.34 (-168.74 to 303.78) | ST | 0.65 | |
| IL-6 | BCG | 52 | -3464.53 (-8986.70 to 1632.67) | ST | 0.33 |  | | IL-6 | | BCG | 18 | -3418.85 (-10478.86 to 2222.30) | WSR | 0.29 | |
| IL-7 | BCG | 57 | -5.14 (-18.59 to 0.00) | ST | 0.016 |  | | IL-7 | | BCG | 19 | 0.00 (-11.18 to 22.28) | WSR | 0.49 | |
| IL-8 | BCG | 45 | -32611.63 (-59915.50 to 6321.33) | ST | 0.23 |  | | IL-8 | | BCG | 13 | -12982.77 (-182201.25 to 145524.16) | WSR | 0.86 | |
| IL-9 | BCG | 57 | -46.77 (-108.84 to 3.47) | ST | 0.063 |  | | IL-9 | | BCG | 19 | 37.82 (-140.34 to 145.48) | WSR | 0.84 | |
| IL-10 | BCG | 57 | -61.76 (-151.99 to 8.36) | WSR | 0.064 |  | | IL-10 | | BCG | 19 | 28.56 (-168.97 to 113.54) | WSR | 0.97 | |
| IL-12p70 | BCG | 57 | -0.73 (-5.41 to 0.00) | ST | 0.072 |  | | IL-12p70 | | BCG | 19 | -0.76 (-11.36 to 13.69) | WSR | 0.90 | |
| IL-12p40 | BCG | 57 | -91.41 (-180.69 to -24.96) | ST | 0.0046 |  | | IL-12p40 | | BCG | 19 | 0.00 (-122.28 to 193.37) | WSR | 0.95 | |
| IL-13 | BCG | 57 | 0.00 (-2.83 to 1.55) | WSR | 0.75 |  | | IL-13 | | BCG | 19 | -1.70 (-6.25 to 2.99) | WSR | 0.3 | |
| IL-15 | BCG | 57 | -82.97 (-217.99 to -11.21) | WSR | 0.032 |  | | IL-15 | | BCG | 19 | -55.29 (-126.77 to 139.63) | ST | 1.0 | |
| IL-16 | BCG | 57 | -25.29 (-60.59 to 2.53) | ST | 0.11 |  | | IL-16 | | BCG | 19 | 11.54 (-35.09 to 66.59) | ST | 0.65 | |
| IL-17 | BCG | 57 | -4.45 (-17.23 to 5.69) | ST | 0.69 |  | | IL-17 | | BCG | 19 | -4.45 (-21.81 to 19.63) | WSR | 0.87 | |
| IL-18 | BCG | 57 | 2.89 (-3.86 to 12.21) | ST | 0.43 |  | | IL-18 | | BCG | 19 | 17.17 (-8.19 to 38.62) | WSR | 0.053 | |
| IP-10 | BCG | 57 | -90.28 (-231.12 to 136.21) | WSR | 0.54 |  | | IP-10 | | BCG | 19 | -171.35 (-302.47 to 182.51) | ST | 0.65 | |
| LIF | BCG | 57 | -14.89 (-38.36 to 8.10) | ST | 0.14 |  | | LIF | | BCG | 19 | -37.78 (-87.50 to 52.94) | ST | 0.36 | |
| MCP-1 | BCG | 37 | -1405.92 (-4316.57 to 4530.40) | ST | 0.74 |  | | MCP-1 | | BCG | 9 | -2073.83 (-15183.77 to 28616.70) | WSR | 0.59 | |
| MCP-3 | BCG | 57 | -169.38 (-288.34 to -37.69) | WSR | 0.0049 |  | | MCP-3 | | BCG | 19 | -6.83 (-275.07 to 176.53) | ST | 1.0 | |
| M-CSF | BCG | 57 | -5.98 (-11.71 to 1.42) | ST | 0.11 |  | | M-CSF | | BCG | 19 | -2.46 (-9.98 to 11.68) | WSR | 0.94 | |
| MIF | BCG | 57 | -255.26 (-603.11 to 257.88) | ST | 0.79 |  | | MIF | | BCG | 19 | -80.17 (-334.96 to 168.55) | WSR | 0.57 | |
| MIG | BCG | 57 | -39.54 (-169.97 to 56.06) | WSR | 0.25 |  | | MIG | | BCG | 19 | -69.47 (-254.09 to 11.81) | WSR | 0.12 | |
| MIP-1𝛼 | BCG | 46 | -157.35 (-514.99 to 85.77) | WSR | 0.17 |  | | MIP-1𝛼 | | BCG | 19 | 240.72 (-128.74 to 1068.37) | ST | 0.65 | |
| MIP-1𝛽 | BCG | 15 | 2198.02 (-48858.63 to 66257.98) | WSR | 0.61 |  | | MIP-1𝛽 | | BCG | 4 | -21508.66 (-56531.45 to 3689.79) | WSR | 0.47 | |
| 𝛽-NGF | BCG | 57 | -4.22 (-9.06 to -1.07) | WSR | 0.074 |  | | 𝛽-NGF | | BCG | 19 | 0.66 (-7.26 to 14.08) | ST | 1.0 | |
| PDGF-BB | BCG | 57 | -80.36 (-212.39 to 80.28) | WSR | 0.44 |  | | PDGF-BB | | BCG | 19 | 198.34 (-5.13 to 366.01) | ST | 0.064 | |
| RANTES | BCG | 57 | -203.31 (-1258.42 to 393.70) | ST | 0.29 |  | | RANTES | | BCG | 19 | 921.64 (-516.46 to 1279.57) | WSR | 0.091 | |
| SCF | BCG | 57 | -29.56 (-59.28 to -11.97) | ST | 0.00046 |  | | SCF | | BCG | 19 | -4.43 (-46.12 to 33.90) | ST | 1.0 | |
| SCGF-𝛽 | BCG | 57 | -5064.43 (-7207.62 to -476.43) | WSR | 0.024 |  | | SCGF-𝛽 | | BCG | 19 | -623.80 (-5344.91 to 6181.10) | WSR | 0.84 | |
| SDF-1𝛼 | BCG | 57 | -33.97 (-161.61 to 47.61) | ST | 1.0 |  | | SDF-1𝛼 | | BCG | 19 | 85.42 (-47.62 to 200.35) | WSR | 0.15 | |
| TNF-𝛼 | BCG | 57 | -413.81 (-1595.21 to 901.95) | WSR | 0.28 |  | | TNF-𝛼 | | BCG | 19 | -923.44 (-2263.62 to 3571.04) | WSR | 0.72 | |
| TNF-𝛽 | BCG | 57 | -49.86 (-151.05 to 70.77) | ST | 0.43 |  | | TNF-𝛽 | | BCG | 19 | 110.11 (-107.29 to 218.47) | WSR | 0.63 | |
| TRAIL | BCG | 57 | -36.90 (-134.44 to 114.85) | ST | 0.29 |  | | TRAIL | | BCG | 19 | 40.04 (-95.28 to 212.82) | ST | 0.65 | |
| VEGF | BCG | 57 | -84.59 (-340.29 to 26.09) | ST | 0.23 |  | | VEGF | | BCG | 19 | -108.76 (-246.69 to 209.90) | ST | 0.36 | |
| CTACK | *C. albicans* | 56 | 15.37 (-8.60 to 36.66) | WSR | 0.29 |  | | CTACK | | *C. albicans* | 19 | -10.44 (-90.88 to 206.79) | WSR | 0.66 | |
| EOTAXIN | *C. albicans* | 56 | -3.00 (-7.97 to 5.40) | WSR | 0.60 |  | | EOTAXIN | | *C. albicans* | 19 | 4.70 (-0.95 to 13.52) | WSR | 0.099 | |
| FGF-basic | *C. albicans* | 56 | 13.37 (-19.54 to 43.22) | WSR | 0.24 |  | | FGF-basic | | *C. albicans* | 19 | 3.39 (-49.18 to 25.81) | WSR | 0.75 | |
| G-CSF | *C. albicans* | 56 | 1374.20 (-293.23 to 5134.24) | ST | 0.14 |  | | G-CSF | | *C. albicans* | 19 | -1680.25 (-5427.81 to 6422.31) | WSR | 0.69 | |
| GM-CSF | *C. albicans* | 56 | 0.50 (-1.00 to 4.11) | WSR | 0.33 |  | | GM-CSF | | *C. albicans* | 19 | 0.84 (-9.59 to 3.06) | WSR | 0.84 | |
| GRO-𝛼 | *C. albicans* | 56 | 2506.78 (650.99 to 6349.28) | WSR | 0.019 |  | | GRO-𝛼 | | *C. albicans* | 19 | -641.45 (-5844.45 to 1953.21) | WSR | 0.44 | |
| HGF | *C. albicans* | 56 | 34.70 (-45.50 to 146.37) | WSR | 0.19 |  | | HGF | | *C. albicans* | 19 | -0.78 (-249.03 to 226.63) | WSR | 0.75 | |
| IFN-𝛼2 | *C. albicans* | 56 | 1.72 (-3.21 to 11.72) | WSR | 0.24 |  | | IFN-𝛼2 | | *C. albicans* | 19 | 0.00 (-14.51 to 10.69) | WSR | 0.98 | |
| IFN-𝛾 | *C. albicans* | 56 | -1.20 (-14.88 to 13.15) | WSR | 0.58 |  | | IFN-𝛾 | | *C. albicans* | 19 | 3.91 (-116.99 to 49.45) | ST | 1.0 | |
| IL-1𝛼 | *C. albicans* | 56 | 10.80 (-8.32 to 44.06) | WSR | 0.24 |  | | IL-1𝛼 | | *C. albicans* | 19 | 11.29 (-59.63 to 64.29) | ST | 0.65 | |
| IL-1𝛽 | *C. albicans* | 56 | 8.38 (-1.77 to 22.87) | WSR | 0.18 |  | | IL-1𝛽 | | *C. albicans* | 19 | -14.69 (-30.92 to 51.90) | ST | 1.0 | |
| IL-1Ra | *C. albicans* | 56 | 114.02 (-299.53 to 1008.48) | WSR | 0.32 |  | | IL-1Ra | | *C. albicans* | 19 | 86.43 (-1595.70 to 1943.30) | WSR | 0.66 | |
| IL-2 | *C. albicans* | 56 | 7.87 (0.00 to 23.71) | WSR | 0.14 |  | | IL-2 | | *C. albicans* | 19 | 19.62 (-63.78 to 65.23) | WSR | 0.81 | |
| IL-2R𝛼 | *C. albicans* | 56 | 6.67 (3.12 to 29.28) | WSR | 0.13 |  | | IL-2R𝛼 | | *C. albicans* | 19 | -12.02 (-43.21 to 33.16) | WSR | 0.72 | |
| IL-3 | *C. albicans* | 56 | 0.26 (0.00 to 1.71) | WSR | 0.016 |  | | IL-3 | | *C. albicans* | 19 | 0.00 (-1.04 to 1.63) | ST | 0.42 | |
| IL-4 | *C. albicans* | 56 | 1.99 (-0.73 to 5.23) | WSR | 0.20 |  | | IL-4 | | *C. albicans* | 19 | -1.45 (-12.54 to 10.25) | WSR | 0.75 | |
| IL-5 | *C. albicans* | 56 | 59.25 (-49.95 to 273.07) | WSR | 0.15 |  | | IL-5 | | *C. albicans* | 19 | 71.00 (-383.45 to 539.87) | WSR | 0.46 | |
| IL-6 | *C. albicans* | 56 | 664.99 (61.08 to 1264.54) | ST | 0.022 |  | | IL-6 | | *C. albicans* | 19 | -95.08 (-2263.77 to 4426.18) | WSR | 0.87 | |
| IL-7 | *C. albicans* | 56 | 0.00 (0.00 to 0.00) | ST | 0.20 |  | | IL-7 | | *C. albicans* | 19 | 0.00 (-15.34 to 6.56) | ST | 0.79 | |
| IL-8 | *C. albicans* | 51 | 8583.86 (1128.49 to 17681.19) | ST | 0.0046 |  | | IL-8 | | *C. albicans* | 17 | 3194.90 (-3931.48 to 37340.55) | WSR | 0.38 | |
| IL-9 | *C. albicans* | 56 | 38.80 (-20.08 to 104.37) | WSR | 0.068 |  | | IL-9 | | *C. albicans* | 19 | -45.82 (-207.54 to 55.88) | WSR | 0.18 | |
| IL-10 | *C. albicans* | 56 | 1.14 (-4.24 to 11.36) | ST | 0.42 |  | | IL-10 | | *C. albicans* | 19 | 5.19 (-14.91 to 41.94) | ST | 0.65 | |
| IL-12p70 | *C. albicans* | 56 | 0.00 (0.00 to 2.21) | WSR | 0.26 |  | | IL-12p70 | | *C. albicans* | 19 | 0.00 (-8.39 to 3.35) | WSR | 0.69 | |
| IL-12p40 | *C. albicans* | 56 | 38.55 (0.00 to 121.26) | WSR | 0.042 |  | | IL-12p40 | | *C. albicans* | 19 | 39.80 (-70.15 to 112.78) | ST | 0.33 | |
| IL-13 | *C. albicans* | 56 | 0.80 (0.00 to 2.40) | WSR | 0.044 |  | | IL-13 | | *C. albicans* | 19 | 0.20 (-4.83 to 1.80) | ST | 0.81 | |
| IL-15 | *C. albicans* | 56 | 42.77 (-21.65 to 123.53) | WSR | 0.24 |  | | IL-15 | | *C. albicans* | 19 | 3.20 (-137.78 to 383.11) | WSR | 0.49 | |
| IL-16 | *C. albicans* | 56 | 11.25 (-3.84 to 38.79) | WSR | 0.14 |  | | IL-16 | | *C. albicans* | 19 | -7.74 (-81.35 to 67.00) | WSR | 0.69 | |
| IL-17 | *C. albicans* | 56 | 5.92 (-8.34 to 24.28) | WSR | 0.12 |  | | IL-17 | | *C. albicans* | 19 | -4.76 (-55.48 to 39.79) | WSR | 0.81 | |
| IL-18 | *C. albicans* | 56 | -0.90 (-6.26 to 3.53) | WSR | 0.52 |  | | IL-18 | | *C. albicans* | 19 | 6.71 (-20.14 to 21.26) | WSR | 0.81 | |
| IP-10 | *C. albicans* | 56 | -20.05 (-174.09 to 89.34) | WSR | 0.56 |  | | IP-10 | | *C. albicans* | 19 | -124.78 (-359.27 to 176.15) | WSR | 0.30 | |
| LIF | *C. albicans* | 56 | 19.08 (0.00 to 67.40) | WSR | 0.088 |  | | LIF | | *C. albicans* | 19 | 3.08 (-123.48 to 78.90) | WSR | 0.84 | |
| MCP-1 | *C. albicans* | 31 | 4429.37 (-6088.11 to 12748.18) | ST | 0.72 |  | | MCP-1 | | *C. albicans* | 9 | 175.68 (-20565.39 to 23418.59) | WSR | 0.77 | |
| MCP-3 | *C. albicans* | 56 | 171.05 (-165.17 to 421.92) | WSR | 0.34 |  | | MCP-3 | | *C. albicans* | 19 | -214.06 (-548.28 to 245.94) | ST | 0.17 | |
| M-CSF | *C. albicans* | 56 | 2.30 (-2.68 to 6.26) | WSR | 0.30 |  | | M-CSF | | *C. albicans* | 19 | 0.00 (-16.23 to 7.79) | WSR | 0.56 | |
| MIF | *C. albicans* | 56 | 37.96 (-144.45 to 310.75) | WSR | 0.55 |  | | MIF | | *C. albicans* | 19 | -77.65 (-403.09 to 230.66) | ST | 1.0 | |
| MIG | *C. albicans* | 56 | 1.50 (-37.71 to 34.59) | ST | 1.0 |  | | MIG | | *C. albicans* | 19 | -14.37 (-140.04 to 48.38) | ST | 1.0 | |
| MIP-1𝛼 | *C. albicans* | 55 | 90.35 (-25.96 to 315.73) | ST | 0.10 |  | | MIP-1𝛼 | | *C. albicans* | 19 | -20.59 (-399.35 to 744.62) | ST | 1.0 | |
| MIP-1𝛽 | *C. albicans* | 56 | 404.39 (-287.30 to 1383.06) | ST | 0.23 |  | | MIP-1𝛽 | | *C. albicans* | 19 | 101.65 (-2829.30 to 1447.86) | ST | 0.65 | |
| 𝛽-NGF | *C. albicans* | 56 | 4.31 (0.30 to 10.19) | WSR | 0.082 |  | | 𝛽-NGF | | *C. albicans* | 19 | 0.00 (-14.41 to 11.88) | WSR | 0.86 | |
| PDGF-BB | *C. albicans* | 56 | 95.17 (-37.11 to 201.67) | WSR | 0.50 |  | | PDGF-BB | | *C. albicans* | 19 | -127.28 (-368.83 to 55.54) | ST | 0.36 | |
| RANTES | *C. albicans* | 56 | 443.87 (-208.50 to 1171.12) | WSR | 0.081 |  | | RANTES | | *C. albicans* | 19 | -513.55 (-1544.50 to 483.24) | WSR | 0.18 | |
| SCF | *C. albicans* | 56 | 5.03 (-25.35 to 58.46) | WSR | 0.49 |  | | SCF | | *C. albicans* | 19 | 18.85 (-84.87 to 61.08) | ST | 1.0 | |
| SCGF-𝛽 | *C. albicans* | 56 | -1072.97 (-3351.26 to 2799.52) | WSR | 0.65 |  | | SCGF-𝛽 | | *C. albicans* | 19 | 3886.24 (-8071.67 to 7477.50) | WSR | 0.90 | |
| SDF-1𝛼 | *C. albicans* | 56 | 27.38 (-114.80 to 88.96) | WSR | 0.79 |  | | SDF-1𝛼 | | *C. albicans* | 19 | -6.37 (-125.23 to 165.50) | WSR | 0.87 | |
| TNF-𝛼 | *C. albicans* | 56 | 60.42 (-40.79 to 141.34) | ST | 0.14 |  | | TNF-𝛼 | | *C. albicans* | 19 | -86.32 (-344.80 to 280.88) | ST | 0.65 | |
| TNF-𝛽 | *C. albicans* | 56 | 77.30 (-38.02 to 252.12) | WSR | 0.073 |  | | TNF-𝛽 | | *C. albicans* | 19 | -155.75 (-364.88 to 162.06) | WSR | 0.20 | |
| TRAIL | *C. albicans* | 56 | 16.28 (-4.98 to 66.01) | WSR | 0.19 |  | | TRAIL | | *C. albicans* | 19 | -18.16 (-182.98 to 66.69) | WSR | 0.52 | |
| VEGF | *C. albicans* | 56 | 0.00 (-196.56 to 93.33) | WSR | 0.81 |  | | VEGF | | *C. albicans* | 19 | -143.99 (-365.29 to 442.14) | WSR | 0.90 | |
| CTACK | *E. coli* | 56 | -69.37 (-116.52 to -22.61) | WSR | 0.0044 |  | | CTACK | | *E. coli* | 19 | -149.31 (-219.61 to -56.60) | WSR | 0.0089 | |
| EOTAXIN | *E. coli* | 56 | -1.26 (-5.89 to 3.37) | WSR | 0.23 |  | | EOTAXIN | | *E. coli* | 19 | 4.03 (-3.59 to 13.56) | ST | 0.36 | |
| FGF-basic | *E. coli* | 56 | -13.34 (-18.38 to -8.41) | ST | 0.010 |  | | FGF-basic | | *E. coli* | 19 | -11.75 (-41.15 to 2.94) | WSR | 0.16 | |
| G-CSF | *E. coli* | 56 | -180.89 (-1571.69 to 989.32) | WSR | 0.71 |  | | G-CSF | | *E. coli* | 19 | -99.82 (-5676.22 to 4343.78) | WSR | 0.63 | |
| GM-CSF | *E. coli* | 56 | -1.47 (-3.97 to 0.48) | ST | 0.50 |  | | GM-CSF | | *E. coli* | 19 | -2.09 (-4.60 to 6.78) | WSR | 0.94 | |
| GRO-𝛼 | *E. coli* | 56 | -33.35 (-412.74 to 469.29) | WSR | 0.95 |  | | GRO-𝛼 | | *E. coli* | 19 | -1254.77 (-1958.57 to 310.30) | ST | 0.36 | |
| HGF | *E. coli* | 56 | -48.63 (-115.09 to -11.88) | ST | 0.022 |  | | HGF | | *E. coli* | 19 | -63.67 (-204.03 to 165.00) | WSR | 0.90 | |
| IFN-𝛼2 | *E. coli* | 56 | -8.62 (-11.65 to -4.18) | ST | 0.00054 |  | | IFN-𝛼2 | | *E. coli* | 19 | 0.07 (-14.40 to 13.14) | ST | 1.0 | |
| IFN-𝛾 | *E. coli* | 56 | -4.89 (-53.43 to 61.46) | ST | 0.89 |  | | IFN-𝛾 | | *E. coli* | 19 | -358.27 (-889.97 to 179.87) | WSR | 0.16 | |
| IL-1𝛼 | *E. coli* | 56 | -3.91 (-133.98 to 45.65) | ST | 0.89 |  | | IL-1𝛼 | | *E. coli* | 19 | -248.65 (-357.74 to 24.91) | WSR | 0.013 | |
| IL-1𝛽 | *E. coli* | 55 | 241.91 (-845.64 to 1091.61) | ST | 0.79 |  | | IL-1𝛽 | | *E. coli* | 19 | -2111.62 (-5385.21 to -574.66) | ST | 0.019 | |
| IL-1Ra | *E. coli* | 56 | -564.33 (-2234.68 to 935.55) | WSR | 0.43 |  | | IL-1Ra | | *E. coli* | 19 | 530.40 (-536.05 to 6261.36) | WSR | 0.11 | |
| IL-2 | *E. coli* | 56 | -29.98 (-55.16 to -5.31) | ST | 0.044 |  | | IL-2 | | *E. coli* | 19 | -26.84 (-74.07 to 48.40) | WSR | 0.35 | |
| IL-2R𝛼 | *E. coli* | 56 | -21.61 (-32.74 to -3.29) | ST | 0.0038 |  | | IL-2R𝛼 | | *E. coli* | 19 | -31.61 (-65.25 to -1.11) | WSR | 0.084 | |
| IL-3 | *E. coli* | 56 | -0.47 (-1.18 to 0.39) | ST | 0.27 |  | | IL-3 | | *E. coli* | 19 | -1.23 (-2.61 to 2.66) | ST | 0.36 | |
| IL-4 | *E. coli* | 56 | -1.87 (-3.84 to -0.57) | ST | 0.0065 |  | | IL-4 | | *E. coli* | 19 | -1.63 (-4.20 to 1.20) | WSR | 0.47 | |
| IL-5 | *E. coli* | 56 | -74.35 (-225.20 to 16.51) | ST | 0.18 |  | | IL-5 | | *E. coli* | 19 | 6.52 (-92.73 to 273.35) | WSR | 0.52 | |
| IL-6 | *E. coli* | 46 | -7902.43 (-13923.85 to 37.32) | ST | 0.054 |  | | IL-6 | | *E. coli* | 18 | -2457.54 (-8469.36 to 3558.31) | WSR | 0.65 | |
| IL-7 | *E. coli* | 56 | 0.00 (-15.67 to 0.00) | WSR | 0.42 |  | | IL-7 | | *E. coli* | 19 | 4.42 (-9.44 to 30.47) | WSR | 0.22 | |
| IL-8 | *E. coli* | 53 | 2211.27 (-535.75 to 7936.56) | WSR | 0.20 |  | | IL-8 | | *E. coli* | 18 | -7744.42 (-10581.29 to 3356.54) | ST | 0.24 | |
| IL-9 | *E. coli* | 56 | -77.51 (-141.54 to -22.15) | WSR | 0.018 |  | | IL-9 | | *E. coli* | 19 | -200.77 (-331.71 to 80.39) | WSR | 0.030 | |
| IL-10 | *E. coli* | 56 | -157.18 (-216.87 to 33.67) | WSR | 0.087 |  | | IL-10 | | *E. coli* | 19 | -307.52 (-442.26 to -110.59) | ST | <0.0001 | |
| IL-12p70 | *E. coli* | 56 | -1.18 (-9.01 to 0.00) | WSR | 0.11 |  | | IL-12p70 | | *E. coli* | 19 | -0.96 (-7.11 to 19.47) | ST | 1.0 | |
| IL-12p40 | *E. coli* | 56 | -966.19 (-1351.30 to -65.81) | ST | 0.044 |  | | IL-12p40 | | *E. coli* | 19 | -209.15 (-2142.90 to 1270.84) | WSR | 0.66 | |
| IL-13 | *E. coli* | 56 | 0.00 (-1.63 to 0.72) | WSR | 0.51 |  | | IL-13 | | *E. coli* | 19 | 0.00 (-3.25 to 2.14) | WSR | 0.98 | |
| IL-15 | *E. coli* | 56 | -42.38 (-132.73 to -1.52) | WSR | 0.043 |  | | IL-15 | | *E. coli* | 19 | -58.77 (-168.65 to 195.79) | ST | 0.65 | |
| IL-16 | *E. coli* | 56 | -13.78 (-28.21 to -1.33) | WSR | 0.064 |  | | IL-16 | | *E. coli* | 19 | -6.47 (-61.10 to 31.48) | WSR | 0.66 | |
| IL-17 | *E. coli* | 56 | -12.55 (-19.98 to -1.98) | ST | 0.0065 |  | | IL-17 | | *E. coli* | 19 | -9.97 (-26.78 to 14.92) | WSR | 0.64 | |
| IL-18 | *E. coli* | 56 | -7.47 (-15.60 to 3.39) | WSR | 0.16 |  | | IL-18 | | *E. coli* | 19 | -16.17 (-27.72 to 2.66) | WSR | 0.16 | |
| IP-10 | *E. coli* | 56 | -800.00 (-1325.75 to 79.46) | WSR | 0.0076 |  | | IP-10 | | *E. coli* | 19 | -200.81 (-1358.84 to 1297.57) | WSR | 0.97 | |
| LIF | *E. coli* | 56 | -21.36 (-44.49 to -2.10) | ST | 0.020 |  | | LIF | | *E. coli* | 19 | -41.98 (-121.27 to 50.03) | WSR | 0.44 | |
| MCP-1 | *E. coli* | 53 | -3.74 (-1626.44 to 1329.33) | ST | 0.78 |  | | MCP-1 | | *E. coli* | 18 | 1648.06 (-3512.56 to 3972.82) | ST | 0.48 | |
| MCP-3 | *E. coli* | 56 | -14.03 (-84.20 to 7.85) | ST | 0.14 |  | | MCP-3 | | *E. coli* | 19 | 21.15 (-87.84 to 109.72) | WSR | 0.84 | |
| M-CSF | *E. coli* | 56 | -6.93 (-10.29 to -2.00) | ST | 0.0065 |  | | M-CSF | | *E. coli* | 19 | -4.92 (-11.31 to -0.39) | WSR | 0.16 | |
| MIF | *E. coli* | 56 | -218.85 (-437.58 to 21.09) | ST | 0.14 |  | | MIF | | *E. coli* | 19 | -437.36 (-1138.72 to 138.73) | ST | 0.65 | |
| MIG | *E. coli* | 56 | -63.23 (-125.99 to 20.91) | WSR | 0.028 |  | | MIG | | *E. coli* | 19 | -245.44 (-355.70 to 220.09) | WSR | 0.26 | |
| MIP-1𝛼 | *E. coli* | 43 | -35.30 (-227.15 to 142.33) | WSR | 0.58 |  | | MIP-1𝛼 | | *E. coli* | 19 | -82.70 (-313.84 to 583.88) | WSR | 0.75 | |
| MIP-1𝛽 | *E. coli* | 3 | 16725.14 (-335.12 to 19491.75) | WSR | 0.29 |  | | MIP-1𝛽 | | *E. coli* | 2 | 3611.86 (-2354.27 to 9577.99) | WSR | 0.65 | |
| 𝛽-NGF | *E. coli* | 56 | -5.03 (-10.58 to 1.73) | ST | 0.22 |  | | 𝛽-NGF | | *E. coli* | 19 | -0.86 (-6.94 to 7.76) | ST | 1.0 | |
| PDGF-BB | *E. coli* | 56 | -203.57 (-299.40 to -59.32) | WSR | 0.0034 |  | | PDGF-BB | | *E. coli* | 19 | -112.80 (-367.84 to 69.91) | WSR | 0.091 | |
| RANTES | *E. coli* | 56 | -583.87 (-1394.89 to 59.80) | WSR | 0.17 |  | | RANTES | | *E. coli* | 19 | -1108.68 (-1970.75 to 109.00) | WSR | 0.013 | |
| SCF | *E. coli* | 56 | -19.97 (-35.16 to -1.06) | ST | 0.044 |  | | SCF | | *E. coli* | 19 | -8.15 (-48.82 to 40.02) | WSR | 0.57 | |
| SCGF-𝛽 | *E. coli* | 56 | -2018.29 (-4682.57 to -833.50) | WSR | 0.024 |  | | SCGF-𝛽 | | *E. coli* | 19 | -2669.85 (-4248.40 to 3862.84) | WSR | 0.60 | |
| SDF-1𝛼 | *E. coli* | 56 | -98.47 (-154.50 to 22.73) | ST | 0.14 |  | | SDF-1𝛼 | | *E. coli* | 19 | -118.77 (-177.44 to 66.30) | WSR | 0.35 | |
| TNF-𝛼 | *E. coli* | 56 | -2724.55 (-5006.96 to 227.46) | ST | 0.23 |  | | TNF-𝛼 | | *E. coli* | 19 | -2129.54 (-7143.66 to 3307.33) | ST | 0.36 | |
| TNF-𝛽 | *E. coli* | 56 | -136.11 (-263.38 to -42.65) | WSR | 0.073 |  | | TNF-𝛽 | | *E. coli* | 19 | -297.77 (-547.53 to -83.73) | WSR | 0.0038 | |
| TRAIL | *E. coli* | 56 | -38.87 (-131.25 to 39.90) | ST | 0.35 |  | | TRAIL | | *E. coli* | 19 | -152.08 (-278.78 to -21.58) | ST | 0.0044 | |
| VEGF | *E. coli* | 56 | -40.82 (-239.16 to 40.01) | WSR | 0.11 |  | | VEGF | | *E. coli* | 19 | 27.87 (-96.79 to 259.16) | WSR | 0.33 | |
| CTACK | R848 | 56 | -43.94 (-65.40 to 42.49) | WSR | 0.27 |  | | CTACK | | R848 | 19 | -69.88 (-229.93 to 193.19) | WSR | 0.49 | |
| EOTAXIN | R848 | 56 | -3.71 (-6.65 to 6.34) | ST | 0.35 |  | | EOTAXIN | | R848 | 19 | 5.40 (-2.41 to 14.52) | WSR | 0.20 | |
| FGF-basic | R848 | 56 | -12.51 (-23.96 to 4.90) | ST | 0.23 |  | | FGF-basic | | R848 | 19 | -10.95 (-37.94 to 22.17) | WSR | 0.66 | |
| G-CSF | R848 | 56 | 489.86 (-1566.92 to 2892.37) | WSR | 0.68 |  | | G-CSF | | R848 | 19 | 193.67 (-3512.44 to 2127.39) | WSR | 0.97 | |
| GM-CSF | R848 | 56 | -3.01 (-6.45 to 1.81) | WSR | 0.077 |  | | GM-CSF | | R848 | 19 | -7.86 (-16.71 to 0.59) | WSR | 0.099 | |
| GRO-𝛼 | R848 | 56 | -14.54 (-299.34 to 166.13) | WSR | 0.19 |  | | GRO-𝛼 | | R848 | 19 | -101.78 (-1095.61 to 373.64) | WSR | 0.20 | |
| HGF | R848 | 56 | -20.23 (-224.38 to 192.54) | WSR | 0.86 |  | | HGF | | R848 | 19 | 21.29 (-670.06 to 564.02) | ST | 1.0 | |
| IFN-𝛼2 | R848 | 56 | -14.84 (-49.43 to 1.26) | ST | 0.081 |  | | IFN-𝛼2 | | R848 | 19 | 2.72 (-75.39 to 33.27) | WSR | 0.75 | |
| IFN-𝛾 | R848 | 56 | 11.54 (-909.72 to 469.04) | ST | 1.0 |  | | IFN-𝛾 | | R848 | 19 | -1842.04 (-5174.29 to 3102.18) | ST | 0.17 | |
| IL-1𝛼 | R848 | 56 | 0.79 (-39.83 to 26.62) | WSR | 0.88 |  | | IL-1𝛼 | | R848 | 19 | -5.30 (-104.67 to 23.38) | WSR | 0.091 | |
| IL-1𝛽 | R848 | 56 | 629.69 (-952.41 to 1920.72) | WSR | 0.68 |  | | IL-1𝛽 | | R848 | 19 | 249.90 (-3015.02 to 2009.02) | ST | 1.0 | |
| IL-1Ra | R848 | 56 | 1650.00 (-178.96 to 2726.24) | WSR | 0.14 |  | | IL-1Ra | | R848 | 19 | 2522.00 (-1684.02 to 8669.11) | ST | 0.65 | |
| IL-2 | R848 | 56 | -9.09 (-40.24 to 20.00) | WSR | 0.46 |  | | IL-2 | | R848 | 19 | -2.25 (-55.49 to 55.62) | WSR | 0.84 | |
| IL-2R𝛼 | R848 | 56 | -12.37 (-45.51 to 8.85) | WSR | 0.074 |  | | IL-2R𝛼 | | R848 | 19 | -21.64 (-64.35 to 24.32) | WSR | 0.21 | |
| IL-3 | R848 | 56 | -0.68 (-2.31 to 1.24) | WSR | 0.27 |  | | IL-3 | | R848 | 19 | 0.47 (-1.95 to 6.23) | ST | 0.65 | |
| IL-4 | R848 | 56 | -1.54 (-3.53 to 0.90) | ST | 0.35 |  | | IL-4 | | R848 | 19 | -1.13 (-6.15 to 3.32) | WSR | 0.42 | |
| IL-5 | R848 | 56 | -21.17 (-231.79 to 101.21) | WSR | 0.42 |  | | IL-5 | | R848 | 19 | -31.62 (-384.21 to 387.17) | WSR | 0.75 | |
| IL-6 | R848 | 52 | -1144.58 (-6553.09 to 5099.64) | ST | 0.89 |  | | IL-6 | | R848 | 19 | -1178.15 (-11546.78 to 5606.92) | WSR | 0.60 | |
| IL-7 | R848 | 56 | 0.00 (-11.33 to 0.00) | WSR | 0.35 |  | | IL-7 | | R848 | 19 | -16.40 (-31.69 to 16.55) | WSR | 0.10 | |
| IL-8 | R848 | 56 | 387.36 (-597.93 to 1772.68) | ST | 0.69 |  | | IL-8 | | R848 | 19 | -560.23 (-2942.38 to 3445.11) | ST | 0.17 | |
| IL-9 | R848 | 56 | -38.91 (-156.10 to 61.16) | WSR | 0.47 |  | | IL-9 | | R848 | 19 | -112.60 (-220.26 to 162.22) | WSR | 0.38 | |
| IL-10 | R848 | 56 | 60.71 (-134.88 to 361.82) | WSR | 0.32 |  | | IL-10 | | R848 | 19 | -33.35 (-355.66 to 55.48) | ST | 0.36 | |
| IL-12p70 | R848 | 56 | -20.49 (-56.33 to 4.67) | ST | 0.14 |  | | IL-12p70 | | R848 | 19 | -17.47 (-86.20 to 98.48) | WSR | 0.60 | |
| IL-12p40 | R848 | 56 | -256.95 (-3549.99 to 5813.36) | WSR | 0.86 |  | | IL-12p40 | | R848 | 19 | 949.39 (-16499.00 to 8163.40) | ST | 1.0 | |
| IL-13 | R848 | 56 | 0.00 (-1.53 to 1.16) | WSR | 0.87 |  | | IL-13 | | R848 | 19 | -2.62 (-4.36 to -0.10) | ST | 0.064 | |
| IL-15 | R848 | 56 | -92.15 (-195.88 to 21.54) | WSR | 0.077 |  | | IL-15 | | R848 | 19 | 71.28 (-102.63 to 115.75) | WSR | 0.81 | |
| IL-16 | R848 | 56 | -9.45 (-33.04 to 12.30) | WSR | 0.23 |  | | IL-16 | | R848 | 19 | -29.69 (-65.40 to 80.60) | WSR | 0.94 | |
| IL-17 | R848 | 56 | -7.14 (-15.94 to 3.32) | WSR | 0.13 |  | | IL-17 | | R848 | 19 | -8.07 (-34.12 to 13.13) | WSR | 0.28 | |
| IL-18 | R848 | 56 | 3.05 (-10.21 to 11.73) | WSR | 0.70 |  | | IL-18 | | R848 | 19 | -1.07 (-42.74 to 31.39) | WSR | 0.81 | |
| IP-10 | R848 | 56 | -162.87 (-1790.63 to 1620.45) | WSR | 0.73 |  | | IP-10 | | R848 | 19 | 1207.04 (-3775.06 to 4613.73) | WSR | 0.75 | |
| LIF | R848 | 56 | -24.89 (-64.89 to 21.74) | ST | 0.69 |  | | LIF | | R848 | 19 | 19.53 (-83.91 to 144.24) | WSR | 0.48 | |
| MCP-1 | R848 | 54 | -263.12 (-1115.26 to 1014.40) | WSR | 0.56 |  | | MCP-1 | | R848 | 18 | -3953.98 (-7489.94 to -1470.92) | ST | 0.031 | |
| MCP-3 | R848 | 56 | -21.66 (-87.29 to 28.97) | WSR | 0.18 |  | | MCP-3 | | R848 | 19 | -105.75 (-379.89 to -22.66) | WSR | 0.011 | |
| M-CSF | R848 | 56 | -1.02 (-6.32 to 3.60) | ST | 0.69 |  | | M-CSF | | R848 | 19 | -1.62 (-12.28 to 7.71) | WSR | 0.66 | |
| MIF | R848 | 56 | 21.95 (-142.49 to 281.78) | WSR | 0.50 |  | | MIF | | R848 | 19 | -89.49 (-405.03 to 481.38) | ST | 0.36 | |
| MIG | R848 | 56 | -155.16 (-329.89 to 190.75) | WSR | 0.57 |  | | MIG | | R848 | 19 | 145.15 (-422.97 to 575.61) | WSR | 0.60 | |
| MIP-1𝛼 | R848 | 45 | 165.16 (-236.43 to 500.83) | ST | 0.37 |  | | MIP-1𝛼 | | R848 | 19 | -88.35 (-898.34 to 825.18) | ST | 1.0 | |
| MIP-1𝛽 | R848 | 5 | 5196.30 (-58279.69 to 36680.78) | WSR | 0.69 |  | | MIP-1𝛽 | | R848 | 2 | 3532.48 (2672.22 to 4392.75) | WSR | 0.18 | |
| 𝛽-NGF | R848 | 56 | -0.29 (-9.45 to 1.97) | ST | 0.68 |  | | 𝛽-NGF | | R848 | 19 | 2.84 (-12.74 to 12.52) | WSR | 0.84 | |
| PDGF-BB | R848 | 56 | -4.31 (-177.23 to 117.87) | WSR | 0.42 |  | | PDGF-BB | | R848 | 19 | 90.59 (-265.60 to 378.79) | WSR | 0.78 | |
| RANTES | R848 | 56 | -141.91 (-526.57 to 118.65) | ST | 0.35 |  | | RANTES | | R848 | 19 | 612.49 (-93.87 to 899.19) | WSR | 0.044 | |
| SCF | R848 | 56 | -13.34 (-31.93 to 6.84) | WSR | 0.21 |  | | SCF | | R848 | 19 | -25.48 (-76.63 to 48.57) | WSR | 0.42 | |
| SCGF-𝛽 | R848 | 56 | -1100.26 (-5320.16 to 1586.54) | ST | 0.23 |  | | SCGF-𝛽 | | R848 | 19 | -2089.57 (-7965.81 to 11271.33) | WSR | 1.0 | |
| SDF-1𝛼 | R848 | 56 | -50.82 (-168.52 to 5.36) | WSR | 0.18 |  | | SDF-1𝛼 | | R848 | 19 | -98.75 (-200.81 to 51.95) | WSR | 0.47 | |
| TNF-𝛼 | R848 | 56 | -2517.23 (-5451.58 to -462.42) | WSR | 0.27 |  | | TNF-𝛼 | | R848 | 19 | 180.39 (-3734.69 to 7751.54) | ST | 1.0 | |
| TNF-𝛽 | R848 | 56 | -111.42 (-211.03 to 145.29) | WSR | 0.39 |  | | TNF-𝛽 | | R848 | 19 | 92.64 (-287.88 to 370.59) | WSR | 0.81 | |
| TRAIL | R848 | 56 | -5.49 (-89.46 to 51.10) | WSR | 0.55 |  | | TRAIL | | R848 | 19 | -135.75 (-243.13 to 297.24) | ST | 0.65 | |
| VEGF | R848 | 56 | -92.06 (-242.41 to 42.61) | WSR | 0.070 |  | | VEGF | | R848 | 19 | 34.22 (-174.09 to 116.30) | ST | 0.65 | |
| CTACK | *S. aureus* | 56 | 38.43 (-7.23 to 84.06) | WSR | 0.037 |  | | CTACK | | *S. aureus* | 19 | -113.99 (-277.21 to 166.86) | WSR | 0.38 | |
| EOTAXIN | *S. aureus* | 56 | -2.03 (-7.70 to 2.93) | WSR | 0.28 |  | | EOTAXIN | | *S. aureus* | 19 | 3.98 (-11.31 to 11.63) | WSR | 0.60 | |
| FGF-basic | *S. aureus* | 56 | 17.82 (-26.95 to 40.64) | WSR | 0.22 |  | | FGF-basic | | *S. aureus* | 19 | -6.76 (-78.06 to 28.32) | WSR | 0.35 | |
| G-CSF | *S. aureus* | 56 | 659.25 (-1248.33 to 4273.64) | ST | 0.35 |  | | G-CSF | | *S. aureus* | 19 | 3756.43 (-8052.80 to 9176.58) | WSR | 0.81 | |
| GM-CSF | *S. aureus* | 56 | 2.95 (-4.53 to 7.18) | WSR | 0.22 |  | | GM-CSF | | *S. aureus* | 19 | -2.91 (-13.63 to 3.18) | WSR | 0.26 | |
| GRO-𝛼 | *S. aureus* | 56 | 3400.51 (924.98 to 7143.63) | WSR | 0.0051 |  | | GRO-𝛼 | | *S. aureus* | 19 | -1062.89 (-7235.21 to 5333.08) | ST | 1.0 | |
| HGF | *S. aureus* | 56 | 8.63 (-81.88 to 155.04) | WSR | 0.10 |  | | HGF | | *S. aureus* | 19 | -0.31 (-316.00 to 224.07) | WSR | 0.70 | |
| IFN-𝛼2 | *S. aureus* | 56 | 0.00 (-7.72 to 7.89) | WSR | 0.88 |  | | IFN-𝛼2 | | *S. aureus* | 19 | -0.58 (-21.81 to 9.79) | WSR | 0.72 | |
| IFN-𝛾 | *S. aureus* | 56 | -9.93 (-28.81 to 3.67) | ST | 0.14 |  | | IFN-𝛾 | | *S. aureus* | 19 | -69.80 (-157.46 to -5.58) | ST | 0.019 | |
| IL-1𝛼 | *S. aureus* | 56 | 34.12 (-20.54 to 107.75) | ST | 0.14 |  | | IL-1𝛼 | | *S. aureus* | 19 | -45.04 (-110.88 to 45.61) | WSR | 0.35 | |
| IL-1𝛽 | *S. aureus* | 56 | 90.82 (-68.97 to 188.56) | ST | 0.35 |  | | IL-1𝛽 | | *S. aureus* | 19 | -74.74 (-204.52 to 41.09) | WSR | 0.42 | |
| IL-1Ra | *S. aureus* | 56 | 185.01 (-818.05 to 1034.47) | WSR | 0.62 |  | | IL-1Ra | | *S. aureus* | 19 | 118.25 (-481.07 to 2463.53) | WSR | 0.42 | |
| IL-2 | *S. aureus* | 56 | 4.49 (-13.56 to 30.21) | ST | 0.89 |  | | IL-2 | | *S. aureus* | 19 | -19.05 (-65.44 to 6.62) | WSR | 0.099 | |
| IL-2R𝛼 | *S. aureus* | 56 | 4.04 (-23.02 to 35.59) | WSR | 0.36 |  | | IL-2R𝛼 | | *S. aureus* | 19 | -10.80 (-79.43 to 45.76) | WSR | 0.35 | |
| IL-3 | *S. aureus* | 56 | -0.09 (-1.48 to 3.06) | WSR | 0.23 |  | | IL-3 | | *S. aureus* | 19 | 1.69 (-4.58 to 5.28) | WSR | 0.78 | |
| IL-4 | *S. aureus* | 56 | 1.61 (-5.24 to 5.34) | WSR | 0.46 |  | | IL-4 | | *S. aureus* | 19 | -2.74 (-13.92 to 3.80) | WSR | 0.28 | |
| IL-5 | *S. aureus* | 56 | 77.41 (-72.80 to 266.38) | WSR | 0.26 |  | | IL-5 | | *S. aureus* | 19 | 2.99 (-641.75 to 467.84) | WSR | 0.69 | |
| IL-6 | *S. aureus* | 53 | 2648.79 (-1129.19 to 6342.98) | ST | 0.27 |  | | IL-6 | | *S. aureus* | 19 | 312.57 (-8669.49 to 5589.91) | ST | 1.0 | |
| IL-7 | *S. aureus* | 56 | 0.00 (-0.44 to 0.00) | WSR | 0.19 |  | | IL-7 | | *S. aureus* | 19 | 0.00 (-3.89 to 22.43) | WSR | 0.29 | |
| IL-8 | *S. aureus* | 48 | 5406.86 (-5248.55 to 29125.34) | WSR | 0.091 |  | | IL-8 | | *S. aureus* | 17 | 10795.35 (-25356.68 to 31430.39) | ST | 0.33 | |
| IL-9 | *S. aureus* | 56 | -7.19 (-61.72 to 57.10) | WSR | 0.97 |  | | IL-9 | | *S. aureus* | 19 | -10.77 (-153.08 to 64.57) | ST | 0.65 | |
| IL-10 | *S. aureus* | 56 | 30.08 (-6.96 to 72.80) | ST | 0.14 |  | | IL-10 | | *S. aureus* | 19 | 21.10 (-60.70 to 51.72) | WSR | 0.90 | |
| IL-12p70 | *S. aureus* | 56 | 0.00 (-0.15 to 0.00) | ST | 0.87 |  | | IL-12p70 | | *S. aureus* | 19 | 1.21 (-1.39 to 11.48) | WSR | 0.17 | |
| IL-12p40 | *S. aureus* | 56 | 49.40 (-63.99 to 190.37) | WSR | 0.077 |  | | IL-12p40 | | *S. aureus* | 19 | 0.00 (-212.93 to 161.06) | ST | 1.0 | |
| IL-13 | *S. aureus* | 56 | 0.56 (-0.17 to 2.04) | ST | 0.32 |  | | IL-13 | | *S. aureus* | 19 | -1.75 (-5.31 to 2.03) | WSR | 0.46 | |
| IL-15 | *S. aureus* | 56 | -2.18 (-50.02 to 143.82) | WSR | 0.59 |  | | IL-15 | | *S. aureus* | 19 | -86.57 (-315.83 to 106.98) | WSR | 0.35 | |
| IL-16 | *S. aureus* | 56 | 27.18 (-6.00 to 88.17) | WSR | 0.082 |  | | IL-16 | | *S. aureus* | 19 | -19.85 (-150.86 to 29.27) | WSR | 0.30 | |
| IL-17 | *S. aureus* | 56 | 12.28 (-11.81 to 51.31) | WSR | 0.046 |  | | IL-17 | | *S. aureus* | 19 | -31.46 (-94.28 to 28.17) | WSR | 0.099 | |
| IL-18 | *S. aureus* | 56 | -0.57 (-6.30 to 4.53) | WSR | 0.94 |  | | IL-18 | | *S. aureus* | 19 | 0.10 (-17.76 to 20.99) | WSR | 0.90 | |
| IP-10 | *S. aureus* | 56 | 46.36 (-237.96 to 406.78) | ST | 0.89 |  | | IP-10 | | *S. aureus* | 19 | -430.13 (-926.78 to -29.32) | ST | 0.064 | |
| LIF | *S. aureus* | 56 | 48.05 (-47.05 to 132.85) | WSR | 0.13 |  | | LIF | | *S. aureus* | 19 | 11.41 (-140.55 to 95.79) | WSR | 0.63 | |
| MCP-1 | *S. aureus* | 28 | -2711.64 (-10030.66 to 2246.02) | ST | 0.34 |  | | MCP-1 | | *S. aureus* | 7 | -6512.37 (-120514.51 to 4568.38) | ST | 0.45 | |
| MCP-3 | *S. aureus* | 56 | -109.84 (-318.01 to 94.96) | WSR | 0.64 |  | | MCP-3 | | *S. aureus* | 19 | -278.79 (-659.87 to 21.60) | WSR | 0.077 | |
| M-CSF | *S. aureus* | 56 | -4.80 (-8.69 to 3.49) | WSR | 0.45 |  | | M-CSF | | *S. aureus* | 19 | -3.26 (-18.90 to 4.68) | WSR | 0.30 | |
| MIF | *S. aureus* | 56 | 111.23 (-104.15 to 294.14) | ST | 0.35 |  | | MIF | | *S. aureus* | 19 | -94.10 (-360.96 to 261.24) | ST | 1.0 | |
| MIG | *S. aureus* | 56 | 9.46 (-65.45 to 64.15) | ST | 0.50 |  | | MIG | | *S. aureus* | 19 | -54.71 (-188.32 to 43.87) | ST | 0.65 | |
| MIP-1𝛼 | *S. aureus* | 49 | 310.38 (-255.18 to 1600.25) | ST | 0.57 |  | | MIP-1𝛼 | | *S. aureus* | 19 | 242.58 (-2309.94 to 1753.90) | WSR | 0.87 | |
| MIP-1𝛽 | *S. aureus* | 52 | 139.32 (-1405.50 to 2483.79) | ST | 1.0 |  | | MIP-1𝛽 | | *S. aureus* | 19 | -2307.13 (-14624.02 to 2011.54) | WSR | 0.15 | |
| 𝛽-NGF | *S. aureus* | 56 | -0.62 (-7.22 to 4.43) | WSR | 0.84 |  | | 𝛽-NGF | | *S. aureus* | 19 | 6.69 (-17.89 to 23.38) | WSR | 0.66 | |
| PDGF-BB | *S. aureus* | 56 | 19.57 (-150.71 to 235.81) | WSR | 0.75 |  | | PDGF-BB | | *S. aureus* | 19 | 53.18 (-505.25 to 247.51) | WSR | 0.57 | |
| RANTES | *S. aureus* | 56 | -353.81 (-792.95 to 708.86) | WSR | 0.66 |  | | RANTES | | *S. aureus* | 19 | -249.55 (-889.03 to 499.82) | ST | 0.65 | |
| SCF | *S. aureus* | 56 | -10.01 (-45.80 to 45.50) | WSR | 0.85 |  | | SCF | | *S. aureus* | 19 | -41.98 (-143.02 to 54.93) | WSR | 0.20 | |
| SCGF-𝛽 | *S. aureus* | 56 | -3915.00 (-7544.18 to -333.75) | ST | 0.022 |  | | SCGF-𝛽 | | *S. aureus* | 19 | 659.53 (-5447.94 to 10854.79) | ST | 1.0 | |
| SDF-1𝛼 | *S. aureus* | 56 | -54.81 (-141.23 to 8.24) | ST | 0.081 |  | | SDF-1𝛼 | | *S. aureus* | 19 | 60.11 (-129.25 to 127.89) | WSR | 0.90 | |
| TNF-𝛼 | *S. aureus* | 56 | 464.18 (-21.91 to 730.35) | WSR | 0.049 |  | | TNF-𝛼 | | *S. aureus* | 19 | -494.98 (-1649.54 to 414.03) | WSR | 0.14 | |
| TNF-𝛽 | *S. aureus* | 56 | 3.65 (-120.09 to 97.60) | WSR | 0.96 |  | | TNF-𝛽 | | *S. aureus* | 19 | -13.75 (-333.55 to 75.63) | ST | 1.0 | |
| TRAIL | *S. aureus* | 56 | 38.76 (-96.03 to 199.77) | WSR | 0.18 |  | | TRAIL | | *S. aureus* | 19 | -67.77 (-223.93 to 35.53) | WSR | 0.23 | |
| VEGF | *S. aureus* | 56 | -78.10 (-221.90 to -4.57) | ST | 0.058 |  | | VEGF | | *S. aureus* | 19 | 152.16 (-313.30 to 322.66) | WSR | 0.87 | |

* Determined by test indicated in ‘Test’ column

ST, Sign test; WSR, Wilcoxon signed-rank test

| **Supplementary Table 4b**: Sensitivity analysis of changes in cytokine stimulation effect (stimulant-control) after 1 dose of ChAdOx1-S (V1-V0) in paired samples with values above ULD replaced as double highest value | | | | | |  | | **Supplementary Table 5b**: Sensitivity analysis of changes in cytokine stimulation effect (stimulant-control) after 2 doses of BNT162b2 (V2-V0) in paired samples with values above ULD replaced as double highest value | | | | | | |
| --- | --- | --- | --- | --- | --- | --- | --- | --- | --- | --- | --- | --- | --- | --- |
| ChAdOx1-S (V1-V0) | | | | | |  | | BNT162b2 (V2-V0) | | | | | | |
| Cytokine | Stimulant | n | Difference in median stimulation effect (95% CI) 28 days after 1 dose of ChAdOx1-S (v1-v0) | Test | p-value* | |  | Cytokine | Stimulant | n | Difference in median stimulation effect (95% CI) 28 days after 2 doses of BNT162b2 (v2-v0) | Test | p-value* |  |
| IL-6 | iSARS | 55 | 89.66 (34.69 to 181.3) | ST | <0.0001 | |  | IL-6 | iSARS | 18 | 359.88 (111.76 to 674.62) | ST | 0.0013 |  |
| IL-8 | iSARS | 55 | -14.82 (-47.99 to 77.63) | WSR | 0.61 | |  | IL-8 | iSARS | 18 | 382.75 (2.35 to 914.24) | ST | 0.096 |  |
| MCP-1 | iSARS | 55 | 4009.52 (2891.45 to 6086.48) | ST | <0.0001 | |  | MCP-1 | iSARS | 18 | 16443.58 (6190.7 to 60766.08) | ST | <0.0001 |  |
| MIP-1𝛼 | iSARS | 55 | 111.87 (78.13 to 164.68) | ST | <0.0001 | |  | MIP-1𝛼 | iSARS | 18 | 232.06 (120.13 to 530.33) | ST | 0.0013 |  |
| MIP-1𝛽 | iSARS | 55 | 1736.25 (1394.35 to 2079.96) | ST | <0.0001 | |  | MIP-1𝛽 | iSARS | 18 | 2328.5 (1756.24 to 3668.13) | WSR | 0.00028 |  |
| IL-6 | BCG | 57 | -1766.91 (-6652.64 to 719.38) | ST | 0.28 | |  | IL-6 | BCG | 19 | -2714.62 (-8103.65 to 1947.62) | WSR | 0.31 |  |
| IL-8 | BCG | 57 | -6528 (-51791.41 to 6.88) | ST | 0.081 | |  | IL-8 | BCG | 19 | 0 (-63213.94 to 24679.91) | ST | 1.0 |  |
| MCP-1 | BCG | 57 | -3867.73 (-9958.69 to -68.71) | WSR | 0.031 | |  | MCP-1 | BCG | 19 | -1184.25 (-35176.13 to 31654.71) | WSR | 0.57 |  |
| MIP-1𝛼 | BCG | 46 | -157.34 (-514.98 to 85.77) | WSR | 0.17 | |  | MIP-1𝛼 | BCG | 19 | 240.71 (-128.74 to 1068.37) | ST | 0.65 |  |
| MIP-1𝛽 | BCG | 56 | -10 (-335.71 to 200.53) | WSR | 0.32 | |  | MIP-1𝛽 | BCG | 19 | 220 (-53.78 to 408.32) | WSR | 0.20 |  |
| IL-6 | *C. albicans* | 56 | 664.98 (61.08 to 1264.53) | ST | 0.022 | |  | IL-6 | *C. albicans* | 19 | -95.08 (-2263.77 to 4426.18) | WSR | 0.87 |  |
| IL-8 | *C. albicans* | 56 | 8025.91 (1014.24 to 17417.91) | ST | 0.010 | |  | IL-8 | *C. albicans* | 19 | 384 (-5199.24 to 20356.46) | ST | 1.0 |  |
| MCP-1 | *C. albicans* | 56 | -148.72 (-5415.7 to 5172.18) | WSR | 0.77 | |  | MCP-1 | *C. albicans* | 19 | -296 (-12096.27 to 9994.79) | WSR | 0.84 |  |
| MIP-1𝛼 | *C. albicans* | 55 | 90.34 (-25.95 to 315.73) | ST | 0.10 | |  | MIP-1𝛼 | *C. albicans* | 19 | -20.59 (-399.34 to 744.61) | ST | 1.0 |  |
| MIP-1𝛽 | *C. albicans* | 56 | 404.38 (-287.3 to 1383.06) | ST | 0.23 | |  | MIP-1𝛽 | *C. albicans* | 19 | 101.65 (-2829.3 to 1447.85) | ST | 0.65 |  |
| IL-6 | *E. coli* | 56 | -4743.77 (-10235.92 to -4) | ST | 0.013 | |  | IL-6 | *E. coli* | 19 | -2845.48 (-9660.95 to 3471.8) | ST | 0.36 |  |
| IL-8 | *E. coli* | 56 | 1832.04 (-1472.57 to 6736.51) | WSR | 0.25 | |  | IL-8 | *E. coli* | 19 | -7636.08 (-9559.97 to 3227.68) | ST | 0.36 |  |
| MCP-1 | *E. coli* | 56 | -14.75 (-1974.38 to 1266.2) | ST | 0.69 | |  | MCP-1 | *E. coli* | 19 | 1772.81 (-3290.79 to 4235.49) | ST | 0.36 |  |
| MIP-1𝛼 | *E. coli* | 43 | -35.29 (-227.14 to 142.32) | WSR | 0.58 | |  | MIP-1𝛼 | *E. coli* | 19 | -82.69 (-313.83 to 583.88) | WSR | 0.75 |  |
| MIP-1𝛽 | *E. coli* | 56 | -8 (-155.08 to 61.62) | WSR | 0.48 | |  | MIP-1𝛽 | *E. coli* | 19 | 220 (-53.78 to 408.32) | ST | 0.17 |  |
| IL-6 | R848 | 56 | -195.81 (-6384.91 to 3503.84) | ST | 0.68 | |  | IL-6 | R848 | 19 | -1178.15 (-11546.78 to 5606.92) | WSR | 0.60 |  |
| IL-8 | R848 | 56 | 387.35 (-597.93 to 1772.67) | ST | 0.69 | |  | IL-8 | R848 | 19 | -560.23 (-2942.38 to 3445.11) | ST | 0.17 |  |
| MCP-1 | R848 | 56 | -263.12 (-1137.63 to 903.35) | WSR | 0.44 | |  | MCP-1 | R848 | 19 | -3062.45 (-7142.59 to -522.9) | ST | 0.064 |  |
| MIP-1𝛼 | R848 | 45 | 165.16 (-236.43 to 500.83) | ST | 0.37 | |  | MIP-1𝛼 | R848 | 19 | -88.34 (-898.34 to 825.18) | ST | 1.0 |  |
| MIP-1𝛽 | R848 | 56 | 10 (-100.35 to 146.72) | WSR | 0.86 | |  | MIP-1𝛽 | R848 | 19 | 260 (-96.8 to 643.34) | WSR | 0.33 |  |
| IL-6 | *S. aureus* | 56 | 2815.82 (-604.93 to 7599.33) | ST | 0.14 | |  | IL-6 | *S. aureus* | 19 | 312.56 (-8669.49 to 5589.91) | ST | 1.0 |  |
| IL-8 | *S. aureus* | 56 | 1789.44 (-3480.38 to 20461.67) | ST | 0.69 | |  | IL-8 | *S. aureus* | 19 | 2075.07 (-27676.71 to 24175.6) | ST | 0.36 |  |
| MCP-1 | *S. aureus* | 56 | -1429.18 (-8885.73 to 131.19) | WSR | 0.19 | |  | MCP-1 | *S. aureus* | 19 | 18.75 (-2553.99 to 748734.43) | WSR | 0.35 |  |
| MIP-1𝛼 | *S. aureus* | 49 | 310.37 (-255.18 to 1600.24) | ST | 0.57 | |  | MIP-1𝛼 | *S. aureus* | 19 | 242.57 (-2309.94 to 1753.9) | WSR | 0.87 |  |
| MIP-1𝛽 | *S. aureus* | 56 | 493.16 (-1339.68 to 2504.74) | ST | 0.89 | |  | MIP-1𝛽 | *S. aureus* | 19 | -2307.13 (-14624.02 to 2011.54) | WSR | 0.15 |  |

* Determined by test indicated in ‘Test’ column

ST, Sign test; WSR, Wilcoxon signed-rank test

| **Supplementary Table 6:** Serum IgG responses 28 days after 2 doses of BNT162b2 compared to ChAdOx1-S vaccination | | | |
| --- | --- | --- | --- |
| IgG | n | GMR (95% CI) | p-value |
| In all included participants with a V2 serology result | | | |
| anti-spike IgG | 225 | 2.97 (2.07 to 4.27) | <0.0001 |
| anti-RBD IgG | 225 | 7.57 (5.35 to 10.70) | <0.0001 |
| In participants who did not receive BCG vaccination in the BRACE trial | | | |
| anti-spike IgG | 74 | 1.85 (1.03 to 3.32) | 0.040 |
| anti-RBD IgG | 74 | 8.61 (4.83 to 15.35) | <0.0001 |
| In participants who did receive BCG vaccination in the BRACE trial | | | |
| anti-spike IgG | 151 | 3.85 (2.42 to 6.11) | <0.001 |
| anti-RBD IgG | 151 | 7.09 (4.59 to 10.94) | <0.001 |

| **Supplementary Table 7a:** Cytokine responses 28 days after 2 doses of BNT162b2 compared to ChAdOx1-S vaccination | | | | |  | | | **Supplementary Table 8a:** Cytokine response 28 days after 2 doses of BNT162b2 compared to ChAdOx1-S vaccination in the subgroup of participants who did not receive BCG vaccination in the BRACE trial | | | | |  | | **Supplementary Table 9a:** Cytokine response 28 days after 2 doses of BNT162b2 compared to ChAdOx1-S vaccination in the subgroup of participants who did receive BCG vaccination in the BRACE trial | | | | | |  |
| --- | --- | --- | --- | --- | --- | --- | --- | --- | --- | --- | --- | --- | --- | --- | --- | --- | --- | --- | --- | --- | --- |
| Cytokine | Stimulant | n | GMR (95% CI) | p-value | |  | Cytokine | | Stimulant | n | GMR (95% CI) | p-value |  | Cytokine | | Stimulant | n | GMR (95% CI) | p-value |  | |
| CTACK | iSARS | 110 | 1.05 (0.97 to 1.12) | 0.23 | |  | CTACK | | iSARS | 33 | 1.03 (0.86 to 1.23) | 0.71 |  | CTACK | | iSARS | 77 | 1.05 (0.97 to 1.15) | 0.22 |  | |
| EOTAXIN | iSARS | 110 | 0.99 (0.92 to 1.06) | 0.73 | |  | EOTAXIN | | iSARS | 33 | 0.95 (0.83 to 1.08) | 0.41 |  | EOTAXIN | | iSARS | 77 | 1 (0.93 to 1.07) | 0.97 |  | |
| FGF-basic | iSARS | 110 | 1.04 (0.93 to 1.17) | 0.47 | |  | FGF-basic | | iSARS | 33 | 1.04 (0.78 to 1.37) | 0.79 |  | FGF-basic | | iSARS | 77 | 1.04 (0.92 to 1.17) | 0.52 |  | |
| G-CSF | iSARS | 110 | 0.94 (0.77 to 1.14) | 0.51 | |  | G-CSF | | iSARS | 33 | 0.90 (0.62 to 1.30) | 0.56 |  | G-CSF | | iSARS | 77 | 0.94 (0.75 to 1.19) | 0.60 |  | |
| GM-CSF | iSARS | 110 | 1.77 (0.91 to 3.46) | 0.094 | |  | GM-CSF | | iSARS | 33 | 1.08 (0.26 to 4.46) | 0.91 |  | GM-CSF | | iSARS | 77 | 1.96 (0.88 to 4.39) | 0.10 |  | |
| GRO-𝛼 | iSARS | 110 | 1.20 (0.87 to 1.65) | 0.27 | |  | GRO-𝛼 | | iSARS | 33 | 1.06 (0.50 to 2.23) | 0.87 |  | GRO-𝛼 | | iSARS | 77 | 1.15 (0.84 to 1.56) | 0.38 |  | |
| HGF | iSARS | 110 | 0.95 (0.83 to 1.08) | 0.42 | |  | HGF | | iSARS | 33 | 0.86 (0.68 to 1.09) | 0.20 |  | HGF | | iSARS | 77 | 0.99 (0.86 to 1.13) | 0.88 |  | |
| IFN-𝛼2 | iSARS | 110 | 0.84 (0.60 to 1.16) | 0.28 | |  | IFN-𝛼2 | | iSARS | 33 | 0.75 (0.34 to 1.62) | 0.44 |  | IFN-𝛼2 | | iSARS | 77 | 0.82 (0.57 to 1.2) | 0.31 |  | |
| IFN-𝛾 | iSARS | 110 | 1.48 (1.14 to 1.93) | 0.004 | |  | IFN-𝛾 | | iSARS | 33 | 1.75 (0.91 to 3.36) | 0.091 |  | IFN-𝛾 | | iSARS | 77 | 1.33 (1 to 1.76) | 0.053 |  | |
| IL-1𝛼 | iSARS | 110 | 1.11 (0.74 to 1.67) | 0.60 | |  | IL-1𝛼 | | iSARS | 33 | 1.70 (0.51 to 5.61) | 0.37 |  | IL-1𝛼 | | iSARS | 77 | 0.97 (0.78 to 1.21) | 0.80 |  | |
| IL-1𝛽 | iSARS | 110 | 0.63 (0.47 to 0.83) | 0.0020 | |  | IL-1𝛽 | | iSARS | 33 | 0.52 (0.29 to 0.92) | 0.027 |  | IL-1𝛽 | | iSARS | 77 | 0.64 (0.46 to 0.89) | 0.0090 |  | |
| IL1RA | iSARS | 110 | 1.11 (0.98 to 1.25) | 0.11 | |  | IL-1Ra | | iSARS | 33 | 1.33 (1.00 to 1.77) | 0.050 |  | IL1RA | | iSARS | 77 | 1.07 (0.93 to 1.23) | 0.35 |  | |
| IL-2 | iSARS | 110 | 1.98 (1.23 to 3.17) | 0.0050 | |  | IL-2 | | iSARS | 33 | 2.55 (0.56 to 11.66) | 0.22 |  | IL-2 | | iSARS | 77 | 1.85 (1.4 to 2.46) | <0.0001 |  | |
| IL-2R𝛼 | iSARS | 110 | 1.07 (0.93 to 1.23) | 0.34 | |  | IL-2R𝛼 | | iSARS | 33 | 1.01 (0.81 to 1.27) | 0.89 |  | IL-2R𝛼 | | iSARS | 77 | 1.05 (0.92 to 1.21) | 0.44 |  | |
| IL-4 | iSARS | 110 | 0.98 (0.84 to 1.13) | 0.74 | |  | IL-4 | | iSARS | 33 | 1.17 (0.79 to 1.75) | 0.42 |  | IL-4 | | iSARS | 77 | 0.92 (0.79 to 1.08) | 0.30 |  | |
| IL-5 | iSARS | 110 | 1.08 (0.61 to 1.90) | 0.80 | |  | IL-5 | | iSARS | 33 | 1.65 (0.41 to 6.65) | 0.47 |  | IL-5 | | iSARS | 77 | 0.83 (0.46 to 1.5) | 0.53 |  | |
| IL-6 | iSARS | 110 | 1.11 (0.80 to 1.54) | 0.53 | |  | IL-6 | | iSARS | 33 | 0.66 (0.31 to 1.42) | 0.28 |  | IL-6 | | iSARS | 77 | 1.28 (0.88 to 1.84) | 0.19 |  | |
| IL-8 | iSARS | 110 | 0.87 (0.68 to 1.11) | 0.25 | |  | IL-8 | | iSARS | 33 | 0.78 (0.46 to 1.32) | 0.34 |  | IL-8 | | iSARS | 77 | 0.87 (0.66 to 1.15) | 0.32 |  | |
| IL-9 | iSARS | 110 | 1.12 (1.02 to 1.22) | 0.014 | |  | IL-9 | | iSARS | 33 | 1.10 (0.92 to 1.32) | 0.29 |  | IL-9 | | iSARS | 77 | 1.1 (0.99 to 1.22) | 0.080 |  | |
| IL-10 | iSARS | 110 | 1.36 (0.95 to 1.95) | 0.091 | |  | IL-10 | | iSARS | 33 | 1.39 (0.42 to 4.58) | 0.58 |  | IL-10 | | iSARS | 77 | 1.22 (0.91 to 1.63) | 0.19 |  | |
| IL-12p70 | iSARS | 110 | 1.44 (0.63 to 3.29) | 0.38 | |  | IL-12p70 | | iSARS | 33 | 2.49 (0.37 to 16.83) | 0.34 |  | IL-12p70 | | iSARS | 77 | 1.08 (0.42 to 2.75) | 0.87 |  | |
| IL-12p40 | iSARS | 110 | 0.60 (0.33 to 1.11) | 0.10 | |  | IL-12p40 | | iSARS | 33 | 1.26 (0.30 to 5.27) | 0.74 |  | IL-12p40 | | iSARS | 77 | 0.45 (0.22 to 0.92) | 0.029 |  | |
| IL-13 | iSARS | 110 | 3.88 (1.99 to 7.55) | <0.0001 | |  | IL-13 | | iSARS | 33 | 7.01 (1.49 to 32.93) | 0.016 |  | IL-13 | | iSARS | 77 | 2.85 (1.36 to 5.98) | 0.0060 |  | |
| IL-15 | iSARS | 110 | 1.01 (0.58 to 1.76) | 0.98 | |  | IL-15 | | iSARS | 33 | 1.04 (0.23 to 4.73) | 0.96 |  | IL-15 | | iSARS | 77 | 0.99 (0.55 to 1.77) | 0.96 |  | |
| IL-16 | iSARS | 110 | 0.91 (0.82 to 1.01) | 0.078 | |  | IL-16 | | iSARS | 33 | 0.88 (0.69 to 1.12) | 0.29 |  | IL-16 | | iSARS | 77 | 0.92 (0.81 to 1.04) | 0.18 |  | |
| IL-17 | iSARS | 110 | 0.95 (0.63 to 1.43) | 0.79 | |  | IL-17 | | iSARS | 33 | 1.17 (0.28 to 4.88) | 0.82 |  | IL-17 | | iSARS | 77 | 0.94 (0.77 to 1.15) | 0.55 |  | |
| IL-18 | iSARS | 110 | 0.98 (0.79 to 1.20) | 0.81 | |  | IL-18 | | iSARS | 33 | 0.79 (0.49 to 1.28) | 0.32 |  | IL-18 | | iSARS | 77 | 0.96 (0.82 to 1.13) | 0.63 |  | |
| IP-10 | iSARS | 110 | 1.02 (0.83 to 1.27) | 0.83 | |  | IP-10 | | iSARS | 33 | 0.91 (0.62 to 1.35) | 0.63 |  | IP-10 | | iSARS | 77 | 1.06 (0.82 to 1.36) | 0.68 |  | |
| LIF | iSARS | 110 | 0.95 (0.67 to 1.35) | 0.79 | |  | LIF | | iSARS | 33 | 1.23 (0.38 to 4.02) | 0.72 |  | LIF | | iSARS | 77 | 0.94 (0.79 to 1.12) | 0.48 |  | |
| MCP-1 | iSARS | 76 | 1.10 (0.68 to 1.78) | 0.70 | |  | MCP-1 | | iSARS | 22 | 0.93 (0.31 to 2.83) | 0.89 |  | MCP-1 | | iSARS | 54 | 1.12 (0.64 to 1.96) | 0.68 |  | |
| MCP-3 | iSARS | 110 | 1.00 (0.80 to 1.26) | 0.99 | |  | MCP-3 | | iSARS | 33 | 0.91 (0.54 to 1.52) | 0.70 |  | MCP-3 | | iSARS | 77 | 1.06 (0.8 to 1.39) | 0.69 |  | |
| M-CSF | iSARS | 110 | 0.98 (0.89 to 1.09) | 0.72 | |  | M-CSF | | iSARS | 33 | 1.00 (0.73 to 1.37) | 1.0 |  | M-CSF | | iSARS | 77 | 0.95 (0.87 to 1.03) | 0.22 |  | |
| MIF | iSARS | 110 | 1.02 (0.94 to 1.10) | 0.64 | |  | MIF | | iSARS | 33 | 0.94 (0.75 to 1.17) | 0.57 |  | MIF | | iSARS | 77 | 1.04 (0.96 to 1.13) | 0.36 |  | |
| MIG | iSARS | 110 | 1.07 (0.89 to 1.29) | 0.46 | |  | MIG | | iSARS | 33 | 1.17 (0.89 to 1.54) | 0.25 |  | MIG | | iSARS | 77 | 1.05 (0.82 to 1.34) | 0.71 |  | |
| MIP-1𝛼 | iSARS | 110 | 0.88 (0.64 to 1.21) | 0.41 | |  | MIP-1𝛼 | | iSARS | 33 | 0.97 (0.55 to 1.71) | 0.90 |  | MIP-1𝛼 | | iSARS | 77 | 0.84 (0.56 to 1.25) | 0.38 |  | |
| MIP-1𝛽 | iSARS | 110 | 0.94 (0.72 to 1.22) | 0.62 | |  | MIP-1𝛽 | | iSARS | 33 | 1.27 (0.82 to 1.97) | 0.28 |  | MIP-1𝛽 | | iSARS | 77 | 0.82 (0.58 to 1.15) | 0.25 |  | |
| 𝛽-NGF | iSARS | 110 | 1.11 (0.78 to 1.59) | 0.57 | |  | 𝛽-NGF | | iSARS | 33 | 1.44 (0.52 to 3.98) | 0.47 |  | 𝛽-NGF | | iSARS | 77 | 0.99 (0.82 to 1.2) | 0.94 |  | |
| PDGF-BB | iSARS | 110 | 0.96 (0.90 to 1.03) | 0.25 | |  | PDGF-BB | | iSARS | 33 | 0.89 (0.78 to 1.01) | 0.069 |  | PDGF-BB | | iSARS | 77 | 0.97 (0.89 to 1.06) | 0.53 |  | |
| RANTES | iSARS | 110 | 1.12 (1.01 to 1.25) | 0.039 | |  | RANTES | | iSARS | 33 | 1.05 (0.87 to 1.26) | 0.60 |  | RANTES | | iSARS | 77 | 1.15 (1 to 1.32) | 0.049 |  | |
| SCF | iSARS | 110 | 1.02 (0.91 to 1.15) | 0.70 | |  | SCF | | iSARS | 33 | 1.05 (0.81 to 1.36) | 0.69 |  | SCF | | iSARS | 77 | 1.03 (0.9 to 1.17) | 0.70 |  | |
| SCGF-𝛽 | iSARS | 110 | 0.97 (0.92 to 1.01) | 0.16 | |  | SCGF-𝛽 | | iSARS | 33 | 0.93 (0.85 to 1.02) | 0.13 |  | SCGF-𝛽 | | iSARS | 77 | 0.97 (0.91 to 1.03) | 0.33 |  | |
| SDF-1𝛼 | iSARS | 110 | 0.97 (0.91 to 1.02) | 0.23 | |  | SDF-1𝛼 | | iSARS | 33 | 0.91 (0.81 to 1.01) | 0.073 |  | SDF-1𝛼 | | iSARS | 77 | 0.98 (0.92 to 1.05) | 0.59 |  | |
| TNF-𝛼 | iSARS | 110 | 0.84 (0.70 to 1.02) | 0.081 | |  | TNF-𝛼 | | iSARS | 33 | 0.80 (0.51 to 1.25) | 0.31 |  | TNF-𝛼 | | iSARS | 77 | 0.84 (0.68 to 1.05) | 0.12 |  | |
| TNF-𝛽 | iSARS | 110 | 1.12 (1.03 to 1.22) | 0.012 | |  | TNF-𝛽 | | iSARS | 33 | 1.09 (0.93 to 1.27) | 0.28 |  | TNF-𝛽 | | iSARS | 77 | 1.12 (1 to 1.25) | 0.044 |  | |
| TRAIL | iSARS | 110 | 1.01 (0.87 to 1.18) | 0.88 | |  | TRAIL | | iSARS | 33 | 0.99 (0.66 to 1.48) | 0.96 |  | TRAIL | | iSARS | 77 | 1 (0.86 to 1.17) | 1.0 |  | |
| VEGF | iSARS | 110 | 0.98 (0.68 to 1.42) | 0.91 | |  | VEGF | | iSARS | 33 | 1.08 (0.31 to 3.81) | 0.90 |  | VEGF | | iSARS | 77 | 0.92 (0.78 to 1.09) | 0.32 |  | |
| CTACK | BCG | 115 | 1.20 (1.04 to 1.38) | 0.012 | |  | CTACK | | BCG | 36 | 1.16 (0.81 to 1.66) | 0.42 |  | CTACK | | BCG | 79 | 1.2 (1.05 to 1.38) | 0.010 |  | |
| EOTAXIN | BCG | 115 | 1.08 (1.00 to 1.16) | 0.038 | |  | EOTAXIN | | BCG | 36 | 1.01 (0.87 to 1.16) | 0.93 |  | EOTAXIN | | BCG | 79 | 1.13 (1.04 to 1.22) | 0.0060 |  | |
| FGF-basic | BCG | 115 | 1.01 (0.91 to 1.12) | 0.89 | |  | FGF-basic | | BCG | 36 | 1.03 (0.80 to 1.32) | 0.84 |  | FGF-basic | | BCG | 79 | 1.03 (0.95 to 1.12) | 0.44 |  | |
| G-CSF | BCG | 115 | 1.19 (1.02 to 1.39) | 0.024 | |  | G-CSF | | BCG | 36 | 1.18 (0.84 to 1.67) | 0.33 |  | G-CSF | | BCG | 79 | 1.2 (1.01 to 1.44) | 0.041 |  | |
| GM-CSF | BCG | 115 | 0.92 (0.76 to 1.10) | 0.36 | |  | GM-CSF | | BCG | 36 | 1.11 (0.70 to 1.75) | 0.65 |  | GM-CSF | | BCG | 79 | 0.83 (0.68 to 1) | 0.052 |  | |
| GRO-𝛼 | BCG | 115 | 1.00 (0.83 to 1.20) | 0.99 | |  | GRO-𝛼 | | BCG | 36 | 1.19 (0.92 to 1.55) | 0.17 |  | GRO-𝛼 | | BCG | 79 | 0.94 (0.74 to 1.2) | 0.62 |  | |
| HGF | BCG | 115 | 1.02 (0.94 to 1.12) | 0.59 | |  | HGF | | BCG | 36 | 1.06 (0.87 to 1.28) | 0.57 |  | HGF | | BCG | 79 | 1.03 (0.94 to 1.14) | 0.54 |  | |
| IFN-𝛼2 | BCG | 115 | 0.89 (0.76 to 1.05) | 0.16 | |  | IFN-𝛼2 | | BCG | 36 | 0.97 (0.63 to 1.51) | 0.89 |  | IFN-𝛼2 | | BCG | 79 | 0.84 (0.73 to 0.96) | 0.013 |  | |
| IFN-𝛾 | BCG | 115 | 1.40 (1.03 to 1.90) | 0.034 | |  | IFN-𝛾 | | BCG | 36 | 1.74 (0.97 to 3.12) | 0.062 |  | IFN-𝛾 | | BCG | 79 | 1.28 (0.89 to 1.85) | 0.17 |  | |
| IL-1𝛼 | BCG | 115 | 0.97 (0.83 to 1.14) | 0.71 | |  | IL-1𝛼 | | BCG | 36 | 1.11 (0.82 to 1.51) | 0.48 |  | IL-1𝛼 | | BCG | 79 | 0.91 (0.76 to 1.09) | 0.30 |  | |
| IL-1𝛽 | BCG | 115 | 0.92 (0.72 to 1.17) | 0.49 | |  | IL-1𝛽 | | BCG | 36 | 1.08 (0.68 to 1.74) | 0.73 |  | IL-1𝛽 | | BCG | 79 | 0.86 (0.64 to 1.18) | 0.35 |  | |
| IL1RA | BCG | 115 | 1.05 (0.89 to 1.24) | 0.54 | |  | IL-1Ra | | BCG | 36 | 1.16 (0.81 to 1.65) | 0.41 |  | IL1RA | | BCG | 79 | 1.09 (0.91 to 1.3) | 0.37 |  | |
| IL-2 | BCG | 115 | 1.02 (0.80 to 1.29) | 0.89 | |  | IL-2 | | BCG | 36 | 0.92 (0.61 to 1.38) | 0.68 |  | IL-2 | | BCG | 79 | 1.06 (0.81 to 1.4) | 0.66 |  | |
| IL-2R𝛼 | BCG | 115 | 1.03 (0.90 to 1.18) | 0.70 | |  | IL-2R𝛼 | | BCG | 36 | 1.13 (0.77 to 1.67) | 0.52 |  | IL-2R𝛼 | | BCG | 79 | 1.01 (0.92 to 1.1) | 0.88 |  | |
| IL-4 | BCG | 115 | 0.93 (0.84 to 1.04) | 0.21 | |  | IL-4 | | BCG | 36 | 1.06 (0.83 to 1.35) | 0.62 |  | IL-4 | | BCG | 79 | 0.91 (0.82 to 1.01) | 0.091 |  | |
| IL-5 | BCG | 115 | 1.04 (0.74 to 1.45) | 0.84 | |  | IL-5 | | BCG | 36 | 1.33 (0.46 to 3.86) | 0.58 |  | IL-5 | | BCG | 79 | 0.89 (0.74 to 1.06) | 0.19 |  | |
| IL-6 | BCG | 109 | 1.25 (0.87 to 1.81) | 0.23 | |  | IL-6 | | BCG | 33 | 1.46 (0.74 to 2.88) | 0.26 |  | IL-6 | | BCG | 76 | 1.19 (0.74 to 1.91) | 0.47 |  | |
| IL-8 | BCG | 82 | 0.82 (0.46 to 1.45) | 0.49 | |  | IL-8 | | BCG | 30 | 0.76 (0.21 to 2.77) | 0.66 |  | IL-8 | | BCG | 52 | 0.85 (0.45 to 1.6) | 0.61 |  | |
| IL-9 | BCG | 115 | 1.10 (1.00 to 1.21) | 0.044 | |  | IL-9 | | BCG | 36 | 1.34 (1.12 to 1.59) | 0.0020 |  | IL-9 | | BCG | 79 | 1.04 (0.93 to 1.16) | 0.52 |  | |
| IL-10 | BCG | 115 | 0.98 (0.75 to 1.27) | 0.86 | |  | IL-10 | | BCG | 36 | 1.16 (0.66 to 2.02) | 0.60 |  | IL-10 | | BCG | 79 | 0.98 (0.73 to 1.3) | 0.87 |  | |
| IL-12p70 | BCG | 115 | 0.91 (0.39 to 2.11) | 0.82 | |  | IL-12p70 | | BCG | 36 | 4.88 (0.97 to 24.65) | 0.055 |  | IL-12p70 | | BCG | 79 | 0.48 (0.18 to 1.29) | 0.14 |  | |
| IL-12p40 | BCG | 115 | 0.90 (0.72 to 1.13) | 0.37 | |  | IL-12p40 | | BCG | 36 | 1.08 (0.50 to 2.35) | 0.84 |  | IL-12p40 | | BCG | 79 | 0.86 (0.76 to 0.98) | 0.024 |  | |
| IL-13 | BCG | 115 | 1.21 (0.73 to 1.99) | 0.46 | |  | IL-13 | | BCG | 36 | 1.16 (0.30 to 4.51) | 0.83 |  | IL-13 | | BCG | 79 | 1.2 (0.73 to 1.97) | 0.46 |  | |
| IL-15 | BCG | 115 | 0.92 (0.67 to 1.27) | 0.62 | |  | IL-15 | | BCG | 36 | 0.95 (0.34 to 2.68) | 0.92 |  | IL-15 | | BCG | 79 | 0.87 (0.74 to 1.03) | 0.096 |  | |
| IL-16 | BCG | 115 | 0.96 (0.89 to 1.03) | 0.27 | |  | IL-16 | | BCG | 36 | 0.96 (0.81 to 1.14) | 0.63 |  | IL-16 | | BCG | 79 | 0.94 (0.87 to 1.02) | 0.13 |  | |
| IL-17 | BCG | 115 | 1.00 (0.88 to 1.14) | 0.99 | |  | IL-17 | | BCG | 36 | 1.03 (0.74 to 1.42) | 0.86 |  | IL-17 | | BCG | 79 | 0.99 (0.89 to 1.12) | 0.93 |  | |
| IL-18 | BCG | 115 | 1.06 (0.82 to 1.37) | 0.64 | |  | IL-18 | | BCG | 36 | 0.99 (0.64 to 1.54) | 0.98 |  | IL-18 | | BCG | 79 | 1.11 (0.93 to 1.33) | 0.25 |  | |
| IP-10 | BCG | 115 | 1.33 (1.04 to 1.69) | 0.024 | |  | IP-10 | | BCG | 36 | 1.49 (1.02 to 2.20) | 0.041 |  | IP-10 | | BCG | 79 | 1.27 (0.93 to 1.74) | 0.13 |  | |
| LIF | BCG | 115 | 1.00 (0.87 to 1.14) | 0.98 | |  | LIF | | BCG | 36 | 1.02 (0.73 to 1.41) | 0.91 |  | LIF | | BCG | 79 | 0.97 (0.87 to 1.08) | 0.60 |  | |
| MCP-1 | BCG | 81 | 1.00 (0.66 to 1.52) | 1.0 | |  | MCP-1 | | BCG | 25 | 0.95 (0.36 to 2.51) | 0.92 |  | MCP-1 | | BCG | 56 | 0.99 (0.6 to 1.66) | 0.98 |  | |
| MCP-3 | BCG | 115 | 0.92 (0.72 to 1.17) | 0.50 | |  | MCP-3 | | BCG | 36 | 0.97 (0.61 to 1.52) | 0.88 |  | MCP-3 | | BCG | 79 | 0.91 (0.66 to 1.24) | 0.54 |  | |
| M-CSF | BCG | 115 | 1.00 (0.77 to 1.29) | 0.98 | |  | M-CSF | | BCG | 36 | 0.83 (0.53 to 1.30) | 0.40 |  | M-CSF | | BCG | 79 | 0.96 (0.86 to 1.07) | 0.49 |  | |
| MIF | BCG | 115 | 1.03 (0.92 to 1.15) | 0.59 | |  | MIF | | BCG | 36 | 1.10 (0.88 to 1.37) | 0.38 |  | MIF | | BCG | 79 | 0.98 (0.86 to 1.11) | 0.71 |  | |
| MIG | BCG | 115 | 1.09 (0.89 to 1.35) | 0.40 | |  | MIG | | BCG | 36 | 1.23 (0.84 to 1.81) | 0.28 |  | MIG | | BCG | 79 | 1.07 (0.82 to 1.38) | 0.63 |  | |
| MIP-1𝛼 | BCG | 102 | 1.43 (0.97 to 2.10) | 0.071 | |  | MIP-1𝛼 | | BCG | 31 | 1.97 (0.84 to 4.63) | 0.11 |  | MIP-1𝛼 | | BCG | 71 | 1.19 (0.76 to 1.87) | 0.44 |  | |
| MIP-1𝛽 | BCG | 37 | 0.69 (0.18 to 2.68) | 0.58 | |  | MIP-1𝛽 | | BCG | 14 | 0.28 (0.03 to 2.45) | 0.19 |  | MIP-1𝛽 | | BCG | 23 | 0.71 (0.13 to 3.94) | 0.67 |  | |
| 𝛽-NGF | BCG | 115 | 1.03 (0.75 to 1.41) | 0.84 | |  | 𝛽-NGF | | BCG | 36 | 1.25 (0.45 to 3.45) | 0.65 |  | 𝛽-NGF | | BCG | 79 | 0.94 (0.79 to 1.11) | 0.43 |  | |
| PDGF-BB | BCG | 115 | 1.07 (0.98 to 1.18) | 0.14 | |  | PDGF-BB | | BCG | 36 | 1.21 (0.97 to 1.50) | 0.085 |  | PDGF-BB | | BCG | 79 | 1.05 (0.95 to 1.17) | 0.31 |  | |
| RANTES | BCG | 115 | 1.19 (1.00 to 1.42) | 0.046 | |  | RANTES | | BCG | 36 | 1.45 (1.09 to 1.92) | 0.011 |  | RANTES | | BCG | 79 | 1.13 (0.9 to 1.42) | 0.28 |  | |
| SCF | BCG | 115 | 0.97 (0.87 to 1.09) | 0.63 | |  | SCF | | BCG | 36 | 1.08 (0.92 to 1.28) | 0.35 |  | SCF | | BCG | 79 | 0.94 (0.84 to 1.05) | 0.26 |  | |
| SCGF-𝛽 | BCG | 115 | 1.07 (0.98 to 1.17) | 0.13 | |  | SCGF-𝛽 | | BCG | 36 | 1.12 (0.90 to 1.39) | 0.31 |  | SCGF-𝛽 | | BCG | 79 | 1.05 (0.96 to 1.15) | 0.27 |  | |
| SDF-1𝛼 | BCG | 115 | 1.06 (1.00 to 1.12) | 0.045 | |  | SDF-1𝛼 | | BCG | 36 | 1.08 (0.97 to 1.20) | 0.17 |  | SDF-1𝛼 | | BCG | 79 | 1.06 (0.99 to 1.14) | 0.094 |  | |
| TNF-𝛼 | BCG | 115 | 1.04 (0.83 to 1.32) | 0.71 | |  | TNF-𝛼 | | BCG | 36 | 1.20 (0.80 to 1.81) | 0.37 |  | TNF-𝛼 | | BCG | 79 | 0.97 (0.72 to 1.3) | 0.83 |  | |
| TNF-𝛽 | BCG | 115 | 1.15 (1.04 to 1.28) | 0.0090 | |  | TNF-𝛽 | | BCG | 36 | 1.33 (1.08 to 1.65) | 0.011 |  | TNF-𝛽 | | BCG | 79 | 1.1 (0.97 to 1.24) | 0.13 |  | |
| TRAIL | BCG | 115 | 1.16 (0.90 to 1.48) | 0.25 | |  | TRAIL | | BCG | 36 | 1.14 (0.86 to 1.52) | 0.34 |  | TRAIL | | BCG | 79 | 1.17 (0.83 to 1.64) | 0.37 |  | |
| VEGF | BCG | 115 | 1.28 (0.82 to 2.00) | 0.27 | |  | VEGF | | BCG | 36 | 2.12 (0.55 to 8.10) | 0.26 |  | VEGF | | BCG | 79 | 0.98 (0.82 to 1.17) | 0.82 |  | |
| CTACK | *C. albicans* | 115 | 1.18 (0.94 to 1.47) | 0.15 | |  | CTACK | | *C. albicans* | 36 | 1.26 (0.77 to 2.06) | 0.35 |  | CTACK | | *C. albicans* | 79 | 1.11 (0.86 to 1.42) | 0.42 |  | |
| EOTAXIN | *C. albicans* | 115 | 1.00 (0.91 to 1.10) | 0.95 | |  | EOTAXIN | | *C. albicans* | 36 | 0.87 (0.72 to 1.06) | 0.17 |  | EOTAXIN | | *C. albicans* | 79 | 1.05 (0.95 to 1.17) | 0.34 |  | |
| FGF-basic | *C. albicans* | 115 | 0.91 (0.74 to 1.11) | 0.33 | |  | FGF-basic | | *C. albicans* | 36 | 1.11 (0.75 to 1.64) | 0.60 |  | FGF-basic | | *C. albicans* | 79 | 0.86 (0.68 to 1.08) | 0.20 |  | |
| G-CSF | *C. albicans* | 115 | 0.86 (0.59 to 1.25) | 0.42 | |  | G-CSF | | *C. albicans* | 36 | 1.14 (0.50 to 2.61) | 0.75 |  | G-CSF | | *C. albicans* | 79 | 0.77 (0.5 to 1.19) | 0.24 |  | |
| GM-CSF | *C. albicans* | 115 | 0.76 (0.37 to 1.55) | 0.45 | |  | GM-CSF | | *C. albicans* | 36 | 1.67 (0.39 to 7.08) | 0.47 |  | GM-CSF | | *C. albicans* | 79 | 0.57 (0.24 to 1.36) | 0.20 |  | |
| GRO-𝛼 | *C. albicans* | 115 | 0.70 (0.44 to 1.11) | 0.13 | |  | GRO-𝛼 | | *C. albicans* | 36 | 1.19 (0.51 to 2.76) | 0.68 |  | GRO-𝛼 | | *C. albicans* | 79 | 0.58 (0.33 to 1.02) | 0.059 |  | |
| HGF | *C. albicans* | 115 | 0.94 (0.79 to 1.12) | 0.49 | |  | HGF | | *C. albicans* | 36 | 1.04 (0.72 to 1.50) | 0.83 |  | HGF | | *C. albicans* | 79 | 0.92 (0.74 to 1.13) | 0.41 |  | |
| IFN-𝛼2 | *C. albicans* | 115 | 0.59 (0.33 to 1.04) | 0.069 | |  | IFN-𝛼2 | | *C. albicans* | 36 | 0.73 (0.24 to 2.28) | 0.58 |  | IFN-𝛼2 | | *C. albicans* | 79 | 0.48 (0.24 to 0.93) | 0.030 |  | |
| IFN-𝛾 | *C. albicans* | 115 | 1.15 (0.90 to 1.48) | 0.27 | |  | IFN-𝛾 | | *C. albicans* | 36 | 1.34 (0.84 to 2.16) | 0.21 |  | IFN-𝛾 | | *C. albicans* | 79 | 1.09 (0.79 to 1.51) | 0.58 |  | |
| IL-1𝛼 | *C. albicans* | 115 | 0.65 (0.41 to 1.02) | 0.062 | |  | IL-1𝛼 | | *C. albicans* | 36 | 0.87 (0.41 to 1.84) | 0.70 |  | IL-1𝛼 | | *C. albicans* | 79 | 0.56 (0.31 to 1.01) | 0.054 |  | |
| IL-1𝛽 | *C. albicans* | 115 | 0.80 (0.49 to 1.32) | 0.38 | |  | IL-1𝛽 | | *C. albicans* | 36 | 1.12 (0.48 to 2.62) | 0.79 |  | IL-1𝛽 | | *C. albicans* | 79 | 0.69 (0.37 to 1.31) | 0.26 |  | |
| IL1RA | *C. albicans* | 115 | 0.95 (0.78 to 1.15) | 0.57 | |  | IL-1Ra | | *C. albicans* | 36 | 1.13 (0.83 to 1.54) | 0.43 |  | IL1RA | | *C. albicans* | 79 | 0.92 (0.73 to 1.16) | 0.48 |  | |
| IL-2 | *C. albicans* | 115 | 0.75 (0.39 to 1.45) | 0.38 | |  | IL-2 | | *C. albicans* | 36 | 0.89 (0.28 to 2.81) | 0.84 |  | IL-2 | | *C. albicans* | 79 | 0.73 (0.31 to 1.69) | 0.45 |  | |
| IL-2R𝛼 | *C. albicans* | 115 | 0.86 (0.69 to 1.06) | 0.15 | |  | IL-2R𝛼 | | *C. albicans* | 36 | 0.91 (0.63 to 1.32) | 0.61 |  | IL-2R𝛼 | | *C. albicans* | 79 | 0.85 (0.66 to 1.1) | 0.21 |  | |
| IL-4 | *C. albicans* | 115 | 0.75 (0.56 to 1.00) | 0.050 | |  | IL-4 | | *C. albicans* | 36 | 1.04 (0.61 to 1.79) | 0.88 |  | IL-4 | | *C. albicans* | 79 | 0.67 (0.48 to 0.95) | 0.026 |  | |
| IL-5 | *C. albicans* | 115 | 0.72 (0.31 to 1.64) | 0.43 | |  | IL-5 | | *C. albicans* | 36 | 1.17 (0.17 to 7.98) | 0.87 |  | IL-5 | | *C. albicans* | 79 | 0.56 (0.22 to 1.4) | 0.21 |  | |
| IL-6 | *C. albicans* | 114 | 0.85 (0.39 to 1.87) | 0.68 | |  | IL-6 | | *C. albicans* | 36 | 1.18 (0.22 to 6.19) | 0.84 |  | IL-6 | | *C. albicans* | 78 | 0.76 (0.31 to 1.87) | 0.54 |  | |
| IL-8 | *C. albicans* | 108 | 0.61 (0.32 to 1.16) | 0.13 | |  | IL-8 | | *C. albicans* | 34 | 1.11 (0.25 to 5.00) | 0.88 |  | IL-8 | | *C. albicans* | 74 | 0.48 (0.25 to 0.96) | 0.038 |  | |
| IL-9 | *C. albicans* | 115 | 1.00 (0.85 to 1.18) | 0.97 | |  | IL-9 | | *C. albicans* | 36 | 1.26 (0.93 to 1.71) | 0.13 |  | IL-9 | | *C. albicans* | 79 | 0.93 (0.76 to 1.13) | 0.45 |  | |
| IL-10 | *C. albicans* | 115 | 0.98 (0.53 to 1.79) | 0.94 | |  | IL-10 | | *C. albicans* | 36 | 1.07 (0.33 to 3.49) | 0.91 |  | IL-10 | | *C. albicans* | 79 | 0.95 (0.45 to 2) | 0.89 |  | |
| IL-12p70 | *C. albicans* | 115 | 1.78 (0.85 to 3.70) | 0.12 | |  | IL-12p70 | | *C. albicans* | 36 | 5.67 (1.71 to 18.78) | 0.0060 |  | IL-12p70 | | *C. albicans* | 79 | 0.97 (0.38 to 2.48) | 0.96 |  | |
| IL-12p40 | *C. albicans* | 115 | 0.67 (0.30 to 1.52) | 0.34 | |  | IL-12p40 | | *C. albicans* | 36 | 1.44 (0.24 to 8.78) | 0.68 |  | IL-12p40 | | *C. albicans* | 79 | 0.5 (0.19 to 1.3) | 0.16 |  | |
| IL-13 | *C. albicans* | 115 | 1.03 (0.47 to 2.26) | 0.94 | |  | IL-13 | | *C. albicans* | 36 | 2.40 (0.48 to 11.90) | 0.27 |  | IL-13 | | *C. albicans* | 79 | 0.8 (0.32 to 2.01) | 0.63 |  | |
| IL-15 | *C. albicans* | 115 | 0.80 (0.40 to 1.58) | 0.51 | |  | IL-15 | | *C. albicans* | 36 | 0.72 (0.16 to 3.24) | 0.66 |  | IL-15 | | *C. albicans* | 79 | 0.82 (0.37 to 1.79) | 0.61 |  | |
| IL-16 | *C. albicans* | 115 | 0.85 (0.68 to 1.06) | 0.15 | |  | IL-16 | | *C. albicans* | 36 | 0.93 (0.58 to 1.48) | 0.74 |  | IL-16 | | *C. albicans* | 79 | 0.81 (0.62 to 1.05) | 0.11 |  | |
| IL-17 | *C. albicans* | 115 | 0.78 (0.55 to 1.11) | 0.17 | |  | IL-17 | | *C. albicans* | 36 | 0.88 (0.45 to 1.74) | 0.70 |  | IL-17 | | *C. albicans* | 79 | 0.75 (0.49 to 1.15) | 0.18 |  | |
| IL-18 | *C. albicans* | 115 | 0.93 (0.58 to 1.48) | 0.76 | |  | IL-18 | | *C. albicans* | 36 | 0.84 (0.61 to 1.15) | 0.26 |  | IL-18 | | *C. albicans* | 79 | 0.91 (0.47 to 1.76) | 0.78 |  | |
| IP-10 | *C. albicans* | 115 | 1.01 (0.76 to 1.34) | 0.95 | |  | IP-10 | | *C. albicans* | 36 | 1.14 (0.73 to 1.78) | 0.55 |  | IP-10 | | *C. albicans* | 79 | 0.97 (0.68 to 1.39) | 0.87 |  | |
| LIF | *C. albicans* | 115 | 0.79 (0.55 to 1.14) | 0.20 | |  | LIF | | *C. albicans* | 36 | 0.99 (0.49 to 2.00) | 0.98 |  | LIF | | *C. albicans* | 79 | 0.68 (0.44 to 1.06) | 0.085 |  | |
| MCP-1 | *C. albicans* | 71 | 0.63 (0.36 to 1.09) | 0.098 | |  | MCP-1 | | *C. albicans* | 21 | 0.34 (0.06 to 1.91) | 0.20 |  | MCP-1 | | *C. albicans* | 50 | 0.65 (0.35 to 1.21) | 0.17 |  | |
| MCP-3 | *C. albicans* | 115 | 0.70 (0.50 to 0.98) | 0.038 | |  | MCP-3 | | *C. albicans* | 36 | 0.88 (0.47 to 1.65) | 0.69 |  | MCP-3 | | *C. albicans* | 79 | 0.65 (0.44 to 0.97) | 0.036 |  | |
| M-CSF | *C. albicans* | 115 | 0.94 (0.73 to 1.21) | 0.64 | |  | M-CSF | | *C. albicans* | 36 | 0.85 (0.59 to 1.22) | 0.36 |  | M-CSF | | *C. albicans* | 79 | 0.88 (0.73 to 1.05) | 0.16 |  | |
| MIF | *C. albicans* | 115 | 1.02 (0.85 to 1.22) | 0.85 | |  | MIF | | *C. albicans* | 36 | 1.08 (0.73 to 1.61) | 0.68 |  | MIF | | *C. albicans* | 79 | 1.01 (0.81 to 1.26) | 0.93 |  | |
| MIG | *C. albicans* | 115 | 0.95 (0.77 to 1.17) | 0.62 | |  | MIG | | *C. albicans* | 36 | 1.04 (0.71 to 1.50) | 0.85 |  | MIG | | *C. albicans* | 79 | 0.94 (0.72 to 1.23) | 0.65 |  | |
| MIP-1𝛼 | *C. albicans* | 110 | 0.72 (0.33 to 1.56) | 0.40 | |  | MIP-1𝛼 | | *C. albicans* | 34 | 1.29 (0.27 to 6.04) | 0.74 |  | MIP-1𝛼 | | *C. albicans* | 76 | 0.58 (0.23 to 1.46) | 0.25 |  | |
| MIP-1𝛽 | *C. albicans* | 114 | 0.81 (0.56 to 1.17) | 0.25 | |  | MIP-1𝛽 | | *C. albicans* | 36 | 1.18 (0.56 to 2.49) | 0.65 |  | MIP-1𝛽 | | *C. albicans* | 78 | 0.71 (0.46 to 1.09) | 0.12 |  | |
| 𝛽-NGF | *C. albicans* | 115 | 1.03 (0.67 to 1.57) | 0.89 | |  | 𝛽-NGF | | *C. albicans* | 36 | 1.42 (0.42 to 4.78) | 0.56 |  | 𝛽-NGF | | *C. albicans* | 79 | 0.88 (0.63 to 1.25) | 0.48 |  | |
| PDGF-BB | *C. albicans* | 115 | 0.96 (0.82 to 1.12) | 0.62 | |  | PDGF-BB | | *C. albicans* | 36 | 1.06 (0.76 to 1.49) | 0.71 |  | PDGF-BB | | *C. albicans* | 79 | 0.96 (0.81 to 1.13) | 0.59 |  | |
| RANTES | *C. albicans* | 115 | 0.98 (0.79 to 1.22) | 0.87 | |  | RANTES | | *C. albicans* | 36 | 1.25 (0.89 to 1.75) | 0.18 |  | RANTES | | *C. albicans* | 79 | 0.91 (0.69 to 1.19) | 0.49 |  | |
| SCF | *C. albicans* | 115 | 0.89 (0.71 to 1.11) | 0.29 | |  | SCF | | *C. albicans* | 36 | 1.15 (0.73 to 1.80) | 0.53 |  | SCF | | *C. albicans* | 79 | 0.8 (0.62 to 1.04) | 0.096 |  | |
| SCGF-𝛽 | *C. albicans* | 115 | 0.96 (0.90 to 1.03) | 0.30 | |  | SCGF-𝛽 | | *C. albicans* | 36 | 1.01 (0.90 to 1.14) | 0.87 |  | SCGF-𝛽 | | *C. albicans* | 79 | 0.95 (0.87 to 1.03) | 0.21 |  | |
| SDF-1𝛼 | *C. albicans* | 115 | 1.00 (0.93 to 1.08) | 0.98 | |  | SDF-1𝛼 | | *C. albicans* | 36 | 0.99 (0.86 to 1.14) | 0.90 |  | SDF-1𝛼 | | *C. albicans* | 79 | 1.01 (0.91 to 1.12) | 0.82 |  | |
| TNF-𝛼 | *C. albicans* | 115 | 0.97 (0.60 to 1.55) | 0.88 | |  | TNF-𝛼 | | *C. albicans* | 36 | 1.12 (0.49 to 2.54) | 0.78 |  | TNF-𝛼 | | *C. albicans* | 79 | 0.92 (0.5 to 1.69) | 0.78 |  | |
| TNF-𝛽 | *C. albicans* | 115 | 1.00 (0.83 to 1.20) | 0.98 | |  | TNF-𝛽 | | *C. albicans* | 36 | 1.26 (0.92 to 1.74) | 0.14 |  | TNF-𝛽 | | *C. albicans* | 79 | 0.92 (0.73 to 1.15) | 0.45 |  | |
| TRAIL | *C. albicans* | 115 | 0.85 (0.64 to 1.15) | 0.30 | |  | TRAIL | | *C. albicans* | 36 | 1.03 (0.60 to 1.78) | 0.91 |  | TRAIL | | *C. albicans* | 79 | 0.79 (0.54 to 1.15) | 0.21 |  | |
| VEGF | *C. albicans* | 115 | 1.25 (0.75 to 2.07) | 0.38 | |  | VEGF | | *C. albicans* | 36 | 1.30 (0.40 to 4.17) | 0.65 |  | VEGF | | *C. albicans* | 79 | 1.22 (0.7 to 2.11) | 0.47 |  | |
| CTACK | *E. coli* | 115 | 1.22 (1.05 to 1.43) | 0.011 | |  | CTACK | | *E. coli* | 36 | 1.25 (0.90 to 1.73) | 0.18 |  | CTACK | | *E. coli* | 79 | 1.2 (1.01 to 1.43) | 0.042 |  | |
| EOTAXIN | *E. coli* | 115 | 1.02 (0.93 to 1.12) | 0.64 | |  | EOTAXIN | | *E. coli* | 36 | 0.95 (0.80 to 1.13) | 0.57 |  | EOTAXIN | | *E. coli* | 79 | 1.07 (0.96 to 1.2) | 0.22 |  | |
| FGF-basic | *E. coli* | 115 | 1.05 (0.94 to 1.18) | 0.39 | |  | FGF-basic | | *E. coli* | 36 | 1.02 (0.80 to 1.30) | 0.86 |  | FGF-basic | | *E. coli* | 79 | 1.09 (0.96 to 1.23) | 0.17 |  | |
| G-CSF | *E. coli* | 115 | 1.14 (0.97 to 1.34) | 0.12 | |  | G-CSF | | *E. coli* | 36 | 1.02 (0.72 to 1.45) | 0.89 |  | G-CSF | | *E. coli* | 79 | 1.2 (0.98 to 1.46) | 0.071 |  | |
| GM-CSF | *E. coli* | 115 | 0.95 (0.81 to 1.10) | 0.49 | |  | GM-CSF | | *E. coli* | 36 | 0.99 (0.70 to 1.41) | 0.97 |  | GM-CSF | | *E. coli* | 79 | 0.94 (0.79 to 1.11) | 0.43 |  | |
| GRO-𝛼 | *E. coli* | 115 | 1.07 (0.93 to 1.22) | 0.34 | |  | GRO-𝛼 | | *E. coli* | 36 | 1.11 (0.85 to 1.46) | 0.43 |  | GRO-𝛼 | | *E. coli* | 79 | 1.06 (0.9 to 1.24) | 0.50 |  | |
| HGF | *E. coli* | 115 | 1.07 (0.96 to 1.20) | 0.22 | |  | HGF | | *E. coli* | 36 | 1.06 (0.84 to 1.34) | 0.63 |  | HGF | | *E. coli* | 79 | 1.1 (0.97 to 1.25) | 0.13 |  | |
| IFN-𝛼2 | *E. coli* | 115 | 0.93 (0.82 to 1.06) | 0.29 | |  | IFN-𝛼2 | | *E. coli* | 36 | 0.93 (0.69 to 1.26) | 0.63 |  | IFN-𝛼2 | | *E. coli* | 79 | 0.92 (0.8 to 1.06) | 0.22 |  | |
| IFN-𝛾 | *E. coli* | 115 | 1.50 (1.06 to 2.11) | 0.022 | |  | IFN-𝛾 | | *E. coli* | 36 | 2.02 (1.07 to 3.80) | 0.031 |  | IFN-𝛾 | | *E. coli* | 79 | 1.31 (0.86 to 2) | 0.21 |  | |
| IL-1𝛼 | *E. coli* | 115 | 1.21 (1.00 to 1.46) | 0.048 | |  | IL-1𝛼 | | *E. coli* | 36 | 1.16 (0.86 to 1.58) | 0.32 |  | IL-1𝛼 | | *E. coli* | 79 | 1.22 (0.96 to 1.55) | 0.11 |  | |
| IL-1𝛽 | *E. coli* | 115 | 1.34 (0.89 to 2.01) | 0.16 | |  | IL-1𝛽 | | *E. coli* | 36 | 0.88 (0.45 to 1.72) | 0.70 |  | IL-1𝛽 | | *E. coli* | 79 | 1.63 (0.96 to 2.75) | 0.070 |  | |
| IL1RA | *E. coli* | 115 | 1.09 (0.92 to 1.29) | 0.34 | |  | IL-1Ra | | *E. coli* | 36 | 1.18 (0.89 to 1.56) | 0.25 |  | IL1RA | | *E. coli* | 79 | 1.1 (0.89 to 1.36) | 0.38 |  | |
| IL-2 | *E. coli* | 115 | 1.16 (0.96 to 1.40) | 0.13 | |  | IL-2 | | *E. coli* | 36 | 1.17 (0.74 to 1.84) | 0.49 |  | IL-2 | | *E. coli* | 79 | 1.14 (0.93 to 1.39) | 0.21 |  | |
| IL-2R𝛼 | *E. coli* | 115 | 1.06 (0.94 to 1.19) | 0.31 | |  | IL-2R𝛼 | | *E. coli* | 36 | 1.03 (0.84 to 1.26) | 0.77 |  | IL-2R𝛼 | | *E. coli* | 79 | 1.09 (0.95 to 1.24) | 0.21 |  | |
| IL-4 | *E. coli* | 115 | 0.97 (0.87 to 1.08) | 0.59 | |  | IL-4 | | *E. coli* | 36 | 1.05 (0.83 to 1.33) | 0.69 |  | IL-4 | | *E. coli* | 79 | 0.95 (0.85 to 1.08) | 0.45 |  | |
| IL-5 | *E. coli* | 115 | 1.28 (0.82 to 2.00) | 0.27 | |  | IL-5 | | *E. coli* | 36 | 1.36 (0.47 to 3.95) | 0.56 |  | IL-5 | | *E. coli* | 79 | 1.25 (0.78 to 2) | 0.35 |  | |
| IL-6 | *E. coli* | 107 | 1.37 (0.94 to 1.99) | 0.10 | |  | IL-6 | | *E. coli* | 34 | 1.54 (0.84 to 2.82) | 0.16 |  | IL-6 | | *E. coli* | 73 | 1.34 (0.8 to 2.23) | 0.26 |  | |
| IL-8 | *E. coli* | 111 | 1.05 (0.81 to 1.36) | 0.71 | |  | IL-8 | | *E. coli* | 36 | 0.94 (0.60 to 1.47) | 0.78 |  | IL-8 | | *E. coli* | 75 | 1.06 (0.77 to 1.45) | 0.73 |  | |
| IL-9 | *E. coli* | 115 | 1.09 (0.98 to 1.20) | 0.12 | |  | IL-9 | | *E. coli* | 36 | 1.21 (0.96 to 1.52) | 0.096 |  | IL-9 | | *E. coli* | 79 | 1.06 (0.95 to 1.19) | 0.28 |  | |
| IL-10 | *E. coli* | 115 | 1.18 (0.87 to 1.60) | 0.28 | |  | IL-10 | | *E. coli* | 36 | 1.30 (0.84 to 2.01) | 0.23 |  | IL-10 | | *E. coli* | 79 | 1.18 (0.79 to 1.75) | 0.41 |  | |
| IL-12p70 | *E. coli* | 115 | 1.48 (0.70 to 3.10) | 0.30 | |  | IL-12p70 | | *E. coli* | 36 | 3.18 (0.82 to 12.27) | 0.090 |  | IL-12p70 | | *E. coli* | 79 | 1.08 (0.42 to 2.75) | 0.87 |  | |
| IL-12p40 | *E. coli* | 115 | 1.26 (0.89 to 1.79) | 0.19 | |  | IL-12p40 | | *E. coli* | 36 | 1.59 (0.73 to 3.46) | 0.23 |  | IL-12p40 | | *E. coli* | 79 | 1.2 (0.8 to 1.81) | 0.37 |  | |
| IL-13 | *E. coli* | 115 | 0.96 (0.58 to 1.59) | 0.88 | |  | IL-13 | | *E. coli* | 36 | 1.42 (0.37 to 5.45) | 0.60 |  | IL-13 | | *E. coli* | 79 | 0.8 (0.49 to 1.3) | 0.37 |  | |
| IL-15 | *E. coli* | 115 | 1.06 (0.70 to 1.59) | 0.78 | |  | IL-15 | | *E. coli* | 36 | 1.19 (0.31 to 4.64) | 0.79 |  | IL-15 | | *E. coli* | 79 | 0.96 (0.83 to 1.1) | 0.54 |  | |
| IL-16 | *E. coli* | 115 | 1.02 (0.91 to 1.15) | 0.69 | |  | IL-16 | | *E. coli* | 36 | 0.94 (0.74 to 1.19) | 0.60 |  | IL-16 | | *E. coli* | 79 | 1.04 (0.9 to 1.19) | 0.61 |  | |
| IL-17 | *E. coli* | 115 | 1.02 (0.89 to 1.17) | 0.75 | |  | IL-17 | | *E. coli* | 36 | 0.95 (0.69 to 1.29) | 0.71 |  | IL-17 | | *E. coli* | 79 | 1.07 (0.92 to 1.25) | 0.35 |  | |
| IL-18 | *E. coli* | 115 | 1.06 (0.88 to 1.27) | 0.55 | |  | IL-18 | | *E. coli* | 36 | 0.92 (0.67 to 1.27) | 0.61 |  | IL-18 | | *E. coli* | 79 | 1.13 (0.91 to 1.41) | 0.25 |  | |
| IP-10 | *E. coli* | 115 | 1.22 (0.92 to 1.62) | 0.17 | |  | IP-10 | | *E. coli* | 36 | 1.22 (0.73 to 2.03) | 0.43 |  | IP-10 | | *E. coli* | 79 | 1.2 (0.84 to 1.72) | 0.31 |  | |
| LIF | *E. coli* | 115 | 1.07 (0.93 to 1.24) | 0.35 | |  | LIF | | *E. coli* | 36 | 1.02 (0.76 to 1.38) | 0.88 |  | LIF | | *E. coli* | 79 | 1.08 (0.91 to 1.28) | 0.36 |  | |
| MCP-1 | *E. coli* | 107 | 0.83 (0.63 to 1.10) | 0.20 | |  | MCP-1 | | *E. coli* | 33 | 0.97 (0.55 to 1.73) | 0.92 |  | MCP-1 | | *E. coli* | 74 | 0.75 (0.53 to 1.05) | 0.090 |  | |
| MCP-3 | *E. coli* | 115 | 0.80 (0.63 to 1.02) | 0.068 | |  | MCP-3 | | *E. coli* | 36 | 0.88 (0.54 to 1.41) | 0.58 |  | MCP-3 | | *E. coli* | 79 | 0.76 (0.55 to 1.03) | 0.075 |  | |
| M-CSF | *E. coli* | 115 | 1.03 (0.88 to 1.21) | 0.71 | |  | M-CSF | | *E. coli* | 36 | 0.95 (0.72 to 1.25) | 0.69 |  | M-CSF | | *E. coli* | 79 | 1.01 (0.88 to 1.15) | 0.88 |  | |
| MIF | *E. coli* | 115 | 1.09 (0.95 to 1.25) | 0.22 | |  | MIF | | *E. coli* | 36 | 1.06 (0.77 to 1.45) | 0.72 |  | MIF | | *E. coli* | 79 | 1.14 (0.98 to 1.34) | 0.092 |  | |
| MIG | *E. coli* | 115 | 1.02 (0.85 to 1.23) | 0.82 | |  | MIG | | *E. coli* | 36 | 1.04 (0.74 to 1.46) | 0.80 |  | MIG | | *E. coli* | 79 | 1.02 (0.8 to 1.3) | 0.87 |  | |
| MIP-1𝛼 | *E. coli* | 103 | 1.49 (1.03 to 2.16) | 0.034 | |  | MIP-1𝛼 | | *E. coli* | 31 | 1.35 (0.66 to 2.76) | 0.39 |  | MIP-1𝛼 | | *E. coli* | 72 | 1.53 (0.96 to 2.46) | 0.076 |  | |
| MIP-1𝛽 | *E. coli* | 21 | 1.53 (0.31 to 7.64) | 0.57 | |  | MIP-1𝛽 | | *E. coli* | 7 | 0.00 (0.00 to 0.00) | <0.0001 |  | MIP-1𝛽 | | *E. coli* | 14 | 1.82 (0.15 to 22.62) | 0.54 |  | |
| 𝛽-NGF | *E. coli* | 115 | 1.19 (0.80 to 1.77) | 0.38 | |  | 𝛽-NGF | | *E. coli* | 36 | 1.52 (0.42 to 5.49) | 0.51 |  | 𝛽-NGF | | *E. coli* | 79 | 1.05 (0.91 to 1.21) | 0.52 |  | |
| PDGF-BB | *E. coli* | 115 | 1.01 (0.91 to 1.12) | 0.83 | |  | PDGF-BB | | *E. coli* | 36 | 1.04 (0.84 to 1.28) | 0.72 |  | PDGF-BB | | *E. coli* | 79 | 1.03 (0.91 to 1.16) | 0.64 |  | |
| RANTES | *E. coli* | 115 | 1.17 (1.01 to 1.36) | 0.041 | |  | RANTES | | *E. coli* | 36 | 1.24 (0.98 to 1.59) | 0.076 |  | RANTES | | *E. coli* | 79 | 1.17 (0.97 to 1.42) | 0.099 |  | |
| SCF | *E. coli* | 115 | 0.98 (0.89 to 1.09) | 0.77 | |  | SCF | | *E. coli* | 36 | 1.02 (0.85 to 1.23) | 0.82 |  | SCF | | *E. coli* | 79 | 0.96 (0.86 to 1.08) | 0.51 |  | |
| SCGF-𝛽 | *E. coli* | 115 | 0.96 (0.88 to 1.04) | 0.26 | |  | SCGF-𝛽 | | *E. coli* | 36 | 0.97 (0.81 to 1.18) | 0.79 |  | SCGF-𝛽 | | *E. coli* | 79 | 0.95 (0.87 to 1.04) | 0.31 |  | |
| SDF-1𝛼 | *E. coli* | 115 | 1.02 (0.95 to 1.10) | 0.54 | |  | SDF-1𝛼 | | *E. coli* | 36 | 1.04 (0.91 to 1.19) | 0.56 |  | SDF-1𝛼 | | *E. coli* | 79 | 1.03 (0.94 to 1.13) | 0.53 |  | |
| TNF-𝛼 | *E. coli* | 115 | 1.35 (0.98 to 1.86) | 0.067 | |  | TNF-𝛼 | | *E. coli* | 36 | 1.28 (0.71 to 2.31) | 0.40 |  | TNF-𝛼 | | *E. coli* | 79 | 1.41 (0.94 to 2.12) | 0.091 |  | |
| TNF-𝛽 | *E. coli* | 115 | 1.17 (1.06 to 1.30) | 0.003 | |  | TNF-𝛽 | | *E. coli* | 36 | 1.28 (1.08 to 1.51) | 0.0070 |  | TNF-𝛽 | | *E. coli* | 79 | 1.15 (1.01 to 1.31) | 0.037 |  | |
| TRAIL | *E. coli* | 115 | 1.11 (0.90 to 1.38) | 0.33 | |  | TRAIL | | *E. coli* | 36 | 0.87 (0.51 to 1.51) | 0.62 |  | TRAIL | | *E. coli* | 79 | 1.22 (0.98 to 1.51) | 0.068 |  | |
| VEGF | *E. coli* | 115 | 1.18 (0.81 to 1.72) | 0.39 | |  | VEGF | | *E. coli* | 36 | 1.52 (0.45 to 5.21) | 0.49 |  | VEGF | | *E. coli* | 79 | 1.01 (0.86 to 1.19) | 0.86 |  | |
| CTACK | R848 | 115 | 1.31 (1.13 to 1.52) | <0.0001 | |  | CTACK | | R848 | 36 | 1.26 (0.91 to 1.73) | 0.15 |  | CTACK | | R848 | 79 | 1.31 (1.11 to 1.55) | 0.0020 |  | |
| EOTAXIN | R848 | 115 | 1.06 (0.99 to 1.13) | 0.12 | |  | EOTAXIN | | R848 | 36 | 0.98 (0.84 to 1.15) | 0.82 |  | EOTAXIN | | R848 | 79 | 1.1 (1.02 to 1.19) | 0.018 |  | |
| FGF-basic | R848 | 115 | 1.08 (0.97 to 1.20) | 0.17 | |  | FGF-basic | | R848 | 36 | 1.07 (0.83 to 1.37) | 0.59 |  | FGF-basic | | R848 | 79 | 1.1 (1 to 1.21) | 0.054 |  | |
| G-CSF | R848 | 115 | 1.22 (1.04 to 1.44) | 0.016 | |  | G-CSF | | R848 | 36 | 1.17 (0.81 to 1.70) | 0.38 |  | G-CSF | | R848 | 79 | 1.25 (1.03 to 1.51) | 0.021 |  | |
| GM-CSF | R848 | 115 | 1.17 (0.84 to 1.64) | 0.35 | |  | GM-CSF | | R848 | 36 | 1.07 (0.76 to 1.51) | 0.68 |  | GM-CSF | | R848 | 79 | 1.2 (0.74 to 1.94) | 0.45 |  | |
| GRO-𝛼 | R848 | 115 | 1.09 (0.85 to 1.40) | 0.50 | |  | GRO-𝛼 | | R848 | 36 | 1.30 (0.57 to 2.97) | 0.52 |  | GRO-𝛼 | | R848 | 79 | 1 (0.91 to 1.09) | 0.96 |  | |
| HGF | R848 | 115 | 1.02 (0.91 to 1.15) | 0.73 | |  | HGF | | R848 | 36 | 1.10 (0.82 to 1.48) | 0.52 |  | HGF | | R848 | 79 | 1 (0.89 to 1.13) | 0.93 |  | |
| IFN-𝛼2 | R848 | 115 | 0.97 (0.82 to 1.16) | 0.77 | |  | IFN-𝛼2 | | R848 | 36 | 1.10 (0.70 to 1.73) | 0.67 |  | IFN-𝛼2 | | R848 | 79 | 0.91 (0.76 to 1.08) | 0.25 |  | |
| IFN-𝛾 | R848 | 115 | 1.71 (1.10 to 2.67) | 0.017 | |  | IFN-𝛾 | | R848 | 36 | 2.72 (1.19 to 6.17) | 0.019 |  | IFN-𝛾 | | R848 | 79 | 1.35 (0.8 to 2.28) | 0.25 |  | |
| IL-1𝛼 | R848 | 115 | 1.01 (0.87 to 1.19) | 0.87 | |  | IL-1𝛼 | | R848 | 36 | 1.02 (0.76 to 1.38) | 0.88 |  | IL-1𝛼 | | R848 | 79 | 0.99 (0.83 to 1.19) | 0.93 |  | |
| IL-1𝛽 | R848 | 115 | 1.15 (0.81 to 1.63) | 0.43 | |  | IL-1𝛽 | | R848 | 36 | 1.04 (0.63 to 1.70) | 0.88 |  | IL-1𝛽 | | R848 | 79 | 1.21 (0.75 to 1.96) | 0.43 |  | |
| IL1RA | R848 | 115 | 1.06 (0.91 to 1.23) | 0.43 | |  | IL-1Ra | | R848 | 36 | 1.13 (0.81 to 1.57) | 0.45 |  | IL1RA | | R848 | 79 | 1.07 (0.91 to 1.26) | 0.41 |  | |
| IL-2 | R848 | 115 | 1.14 (0.93 to 1.40) | 0.21 | |  | IL-2 | | R848 | 36 | 1.12 (0.82 to 1.54) | 0.47 |  | IL-2 | | R848 | 79 | 1.15 (0.87 to 1.51) | 0.31 |  | |
| IL-2R𝛼 | R848 | 115 | 1.10 (0.98 to 1.24) | 0.11 | |  | IL-2R𝛼 | | R848 | 36 | 1.13 (0.90 to 1.42) | 0.29 |  | IL-2R𝛼 | | R848 | 79 | 1.1 (0.96 to 1.25) | 0.16 |  | |
| IL-4 | R848 | 115 | 0.96 (0.88 to 1.06) | 0.41 | |  | IL-4 | | R848 | 36 | 1.05 (0.83 to 1.32) | 0.67 |  | IL-4 | | R848 | 79 | 0.94 (0.86 to 1.03) | 0.21 |  | |
| IL-5 | R848 | 115 | 1.09 (0.78 to 1.52) | 0.63 | |  | IL-5 | | R848 | 36 | 1.39 (0.47 to 4.09) | 0.53 |  | IL-5 | | R848 | 79 | 0.92 (0.78 to 1.08) | 0.30 |  | |
| IL-6 | R848 | 108 | 1.29 (0.85 to 1.93) | 0.22 | |  | IL-6 | | R848 | 35 | 0.90 (0.29 to 2.81) | 0.85 |  | IL-6 | | R848 | 73 | 1.48 (1.01 to 2.16) | 0.044 |  | |
| IL-8 | R848 | 115 | 1.08 (0.80 to 1.45) | 0.62 | |  | IL-8 | | R848 | 36 | 0.94 (0.62 to 1.44) | 0.77 |  | IL-8 | | R848 | 79 | 1.17 (0.8 to 1.71) | 0.42 |  | |
| IL-9 | R848 | 115 | 1.15 (1.02 to 1.31) | 0.025 | |  | IL-9 | | R848 | 36 | 1.36 (1.09 to 1.71) | 0.0090 |  | IL-9 | | R848 | 79 | 1.08 (0.93 to 1.26) | 0.31 |  | |
| IL-10 | R848 | 115 | 1.16 (0.91 to 1.48) | 0.24 | |  | IL-10 | | R848 | 36 | 1.21 (0.71 to 2.08) | 0.47 |  | IL-10 | | R848 | 79 | 1.17 (0.88 to 1.56) | 0.26 |  | |
| IL-12p70 | R848 | 115 | 1.29 (0.83 to 2.02) | 0.25 | |  | IL-12p70 | | R848 | 36 | 1.21 (0.53 to 2.74) | 0.64 |  | IL-12p70 | | R848 | 79 | 1.27 (0.72 to 2.22) | 0.40 |  | |
| IL-12p40 | R848 | 115 | 1.08 (0.76 to 1.53) | 0.66 | |  | IL-12p40 | | R848 | 36 | 1.43 (0.72 to 2.82) | 0.29 |  | IL-12p40 | | R848 | 79 | 1 (0.65 to 1.52) | 0.98 |  | |
| IL-13 | R848 | 115 | 1.15 (0.74 to 1.80) | 0.53 | |  | IL-13 | | R848 | 36 | 1.81 (0.44 to 7.36) | 0.40 |  | IL-13 | | R848 | 79 | 0.91 (0.68 to 1.22) | 0.54 |  | |
| IL-15 | R848 | 115 | 0.97 (0.71 to 1.32) | 0.84 | |  | IL-15 | | R848 | 36 | 1.00 (0.35 to 2.86) | 1.0 |  | IL-15 | | R848 | 79 | 0.91 (0.8 to 1.02) | 0.12 |  | |
| IL-16 | R848 | 115 | 1.02 (0.94 to 1.10) | 0.69 | |  | IL-16 | | R848 | 36 | 0.97 (0.79 to 1.19) | 0.75 |  | IL-16 | | R848 | 79 | 1.04 (0.96 to 1.12) | 0.37 |  | |
| IL-17 | R848 | 115 | 1.05 (0.93 to 1.18) | 0.47 | |  | IL-17 | | R848 | 36 | 1.01 (0.75 to 1.38) | 0.92 |  | IL-17 | | R848 | 79 | 1.06 (0.94 to 1.19) | 0.34 |  | |
| IL-18 | R848 | 115 | 1.02 (0.85 to 1.22) | 0.84 | |  | IL-18 | | R848 | 36 | 0.92 (0.71 to 1.18) | 0.47 |  | IL-18 | | R848 | 79 | 1.09 (0.88 to 1.35) | 0.45 |  | |
| IP-10 | R848 | 115 | 1.12 (0.90 to 1.39) | 0.32 | |  | IP-10 | | R848 | 36 | 0.90 (0.60 to 1.36) | 0.60 |  | IP-10 | | R848 | 79 | 1.26 (0.97 to 1.65) | 0.083 |  | |
| LIF | R848 | 115 | 1.12 (0.98 to 1.27) | 0.092 | |  | LIF | | R848 | 36 | 1.04 (0.76 to 1.41) | 0.81 |  | LIF | | R848 | 79 | 1.14 (1 to 1.3) | 0.056 |  | |
| MCP-1 | R848 | 108 | 1.20 (0.89 to 1.62) | 0.22 | |  | MCP-1 | | R848 | 34 | 1.31 (0.64 to 2.68) | 0.44 |  | MCP-1 | | R848 | 74 | 1.19 (0.85 to 1.66) | 0.31 |  | |
| MCP-3 | R848 | 115 | 0.94 (0.72 to 1.23) | 0.65 | |  | MCP-3 | | R848 | 36 | 1.06 (0.63 to 1.76) | 0.83 |  | MCP-3 | | R848 | 79 | 0.91 (0.65 to 1.26) | 0.56 |  | |
| M-CSF | R848 | 115 | 1.06 (0.90 to 1.25) | 0.46 | |  | M-CSF | | R848 | 36 | 1.00 (0.77 to 1.30) | 1.0 |  | M-CSF | | R848 | 79 | 1.03 (0.9 to 1.18) | 0.67 |  | |
| MIF | R848 | 115 | 1.10 (0.96 to 1.26) | 0.16 | |  | MIF | | R848 | 36 | 1.10 (0.78 to 1.57) | 0.58 |  | MIF | | R848 | 79 | 1.13 (0.98 to 1.3) | 0.080 |  | |
| MIG | R848 | 115 | 1.06 (0.83 to 1.35) | 0.64 | |  | MIG | | R848 | 36 | 1.25 (0.80 to 1.97) | 0.31 |  | MIG | | R848 | 79 | 1 (0.74 to 1.34) | 0.97 |  | |
| MIP-1𝛼 | R848 | 102 | 1.62 (1.08 to 2.42) | 0.020 | |  | MIP-1𝛼 | | R848 | 31 | 1.56 (0.72 to 3.37) | 0.24 |  | MIP-1𝛼 | | R848 | 71 | 1.6 (0.95 to 2.69) | 0.077 |  | |
| MIP-1𝛽 | R848 | 24 | 1.93 (0.28 to 13.18) | 0.47 | |  | MIP-1𝛽 | | R848 | 7 | 0.21 (0.00 to 0.00) | <0.0001 |  | MIP-1𝛽 | | R848 | 17 | 1.23 (0.1 to 16) | 0.85 |  | |
| 𝛽-NGF | R848 | 115 | 1.08 (0.79 to 1.48) | 0.61 | |  | 𝛽-NGF | | R848 | 36 | 1.20 (0.43 to 3.38) | 0.72 |  | 𝛽-NGF | | R848 | 79 | 1 (0.86 to 1.16) | 0.99 |  | |
| PDGF-BB | R848 | 115 | 1.07 (0.97 to 1.17) | 0.19 | |  | PDGF-BB | | R848 | 36 | 1.05 (0.87 to 1.28) | 0.57 |  | PDGF-BB | | R848 | 79 | 1.09 (0.97 to 1.22) | 0.17 |  | |
| RANTES | R848 | 115 | 1.18 (1.01 to 1.39) | 0.043 | |  | RANTES | | R848 | 36 | 1.26 (1.01 to 1.58) | 0.042 |  | RANTES | | R848 | 79 | 1.19 (0.96 to 1.47) | 0.12 |  | |
| SCF | R848 | 115 | 1.01 (0.92 to 1.11) | 0.87 | |  | SCF | | R848 | 36 | 1.07 (0.87 to 1.30) | 0.52 |  | SCF | | R848 | 79 | 0.99 (0.91 to 1.06) | 0.71 |  | |
| SCGF-𝛽 | R848 | 115 | 1.03 (0.97 to 1.10) | 0.34 | |  | SCGF-𝛽 | | R848 | 36 | 1.06 (0.92 to 1.22) | 0.43 |  | SCGF-𝛽 | | R848 | 79 | 1.02 (0.95 to 1.09) | 0.58 |  | |
| SDF-1𝛼 | R848 | 115 | 1.09 (1.01 to 1.18) | 0.019 | |  | SDF-1𝛼 | | R848 | 36 | 1.11 (0.98 to 1.26) | 0.084 |  | SDF-1𝛼 | | R848 | 79 | 1.09 (0.99 to 1.2) | 0.094 |  | |
| TNF-𝛼 | R848 | 115 | 1.24 (1.00 to 1.55) | 0.054 | |  | TNF-𝛼 | | R848 | 36 | 1.40 (0.93 to 2.11) | 0.11 |  | TNF-𝛼 | | R848 | 79 | 1.21 (0.91 to 1.6) | 0.19 |  | |
| TNF-𝛽 | R848 | 115 | 1.23 (1.07 to 1.41) | 0.0040 | |  | TNF-𝛽 | | R848 | 36 | 1.36 (1.07 to 1.71) | 0.013 |  | TNF-𝛽 | | R848 | 79 | 1.19 (1 to 1.43) | 0.055 |  | |
| TRAIL | R848 | 115 | 1.18 (1.02 to 1.37) | 0.023 | |  | TRAIL | | R848 | 36 | 1.11 (0.82 to 1.49) | 0.49 |  | TRAIL | | R848 | 79 | 1.2 (1.01 to 1.43) | 0.037 |  | |
| VEGF | R848 | 115 | 1.09 (0.81 to 1.47) | 0.55 | |  | VEGF | | R848 | 36 | 1.29 (0.49 to 3.35) | 0.60 |  | VEGF | | R848 | 79 | 0.98 (0.86 to 1.12) | 0.73 |  | |
| CTACK | *S. aureus* | 115 | 1.29 (1.00 to 1.66) | 0.053 | |  | CTACK | | *S. aureus* | 36 | 1.07 (0.71 to 1.60) | 0.74 |  | CTACK | | *S. aureus* | 79 | 1.35 (0.97 to 1.89) | 0.076 |  | |
| EOTAXIN | *S. aureus* | 115 | 1.03 (0.90 to 1.18) | 0.70 | |  | EOTAXIN | | *S. aureus* | 36 | 0.88 (0.74 to 1.05) | 0.15 |  | EOTAXIN | | *S. aureus* | 79 | 1.09 (0.91 to 1.31) | 0.35 |  | |
| FGF-basic | *S. aureus* | 115 | 1.08 (0.91 to 1.29) | 0.37 | |  | FGF-basic | | *S. aureus* | 36 | 1.00 (0.73 to 1.38) | 0.98 |  | FGF-basic | | *S. aureus* | 79 | 1.14 (0.92 to 1.43) | 0.23 |  | |
| G-CSF | *S. aureus* | 115 | 1.26 (1.01 to 1.58) | 0.041 | |  | G-CSF | | *S. aureus* | 36 | 1.21 (0.82 to 1.78) | 0.32 |  | G-CSF | | *S. aureus* | 79 | 1.3 (0.97 to 1.74) | 0.076 |  | |
| GM-CSF | *S. aureus* | 115 | 1.28 (0.72 to 2.26) | 0.40 | |  | GM-CSF | | *S. aureus* | 36 | 1.10 (0.38 to 3.21) | 0.86 |  | GM-CSF | | *S. aureus* | 79 | 1.45 (0.69 to 3.03) | 0.32 |  | |
| GRO-𝛼 | *S. aureus* | 115 | 1.13 (0.76 to 1.68) | 0.55 | |  | GRO-𝛼 | | *S. aureus* | 36 | 1.28 (0.72 to 2.27) | 0.39 |  | GRO-𝛼 | | *S. aureus* | 79 | 1.11 (0.65 to 1.9) | 0.70 |  | |
| HGF | *S. aureus* | 115 | 1.08 (0.92 to 1.27) | 0.36 | |  | HGF | | *S. aureus* | 36 | 1.00 (0.75 to 1.33) | 1.0 |  | HGF | | *S. aureus* | 79 | 1.13 (0.92 to 1.39) | 0.25 |  | |
| IFN-𝛼2 | *S. aureus* | 115 | 0.94 (0.69 to 1.30) | 0.72 | |  | IFN-𝛼2 | | *S. aureus* | 36 | 0.82 (0.41 to 1.65) | 0.57 |  | IFN-𝛼2 | | *S. aureus* | 79 | 0.96 (0.67 to 1.39) | 0.83 |  | |
| IFN-𝛾 | *S. aureus* | 115 | 1.40 (1.07 to 1.83) | 0.014 | |  | IFN-𝛾 | | *S. aureus* | 36 | 1.47 (0.80 to 2.69) | 0.21 |  | IFN-𝛾 | | *S. aureus* | 79 | 1.38 (1.01 to 1.87) | 0.040 |  | |
| IL-1𝛼 | *S. aureus* | 115 | 0.99 (0.76 to 1.29) | 0.93 | |  | IL-1𝛼 | | *S. aureus* | 36 | 0.88 (0.55 to 1.39) | 0.57 |  | IL-1𝛼 | | *S. aureus* | 79 | 1.02 (0.72 to 1.44) | 0.90 |  | |
| IL-1𝛽 | *S. aureus* | 115 | 1.11 (0.68 to 1.82) | 0.67 | |  | IL-1𝛽 | | *S. aureus* | 36 | 0.83 (0.42 to 1.62) | 0.57 |  | IL-1𝛽 | | *S. aureus* | 79 | 1.27 (0.66 to 2.44) | 0.47 |  | |
| IL1RA | *S. aureus* | 115 | 1.09 (0.89 to 1.32) | 0.39 | |  | IL-1Ra | | *S. aureus* | 36 | 1.12 (0.83 to 1.50) | 0.45 |  | IL1RA | | *S. aureus* | 79 | 1.12 (0.87 to 1.44) | 0.39 |  | |
| IL-2 | *S. aureus* | 115 | 1.03 (0.69 to 1.54) | 0.89 | |  | IL-2 | | *S. aureus* | 36 | 0.85 (0.47 to 1.54) | 0.58 |  | IL-2 | | *S. aureus* | 79 | 1.15 (0.67 to 1.99) | 0.61 |  | |
| IL-2R𝛼 | *S. aureus* | 115 | 1.03 (0.76 to 1.39) | 0.85 | |  | IL-2R𝛼 | | *S. aureus* | 36 | 0.91 (0.67 to 1.24) | 0.54 |  | IL-2R𝛼 | | *S. aureus* | 79 | 1.1 (0.72 to 1.69) | 0.66 |  | |
| IL-4 | *S. aureus* | 115 | 0.97 (0.80 to 1.17) | 0.73 | |  | IL-4 | | *S. aureus* | 36 | 0.99 (0.74 to 1.33) | 0.95 |  | IL-4 | | *S. aureus* | 79 | 0.98 (0.76 to 1.27) | 0.88 |  | |
| IL-5 | *S. aureus* | 115 | 1.14 (0.67 to 1.94) | 0.63 | |  | IL-5 | | *S. aureus* | 36 | 1.04 (0.36 to 2.99) | 0.93 |  | IL-5 | | *S. aureus* | 79 | 1.13 (0.58 to 2.19) | 0.73 |  | |
| IL-6 | *S. aureus* | 114 | 1.29 (0.78 to 2.13) | 0.33 | |  | IL-6 | | *S. aureus* | 36 | 0.88 (0.37 to 2.07) | 0.76 |  | IL-6 | | *S. aureus* | 78 | 1.61 (0.86 to 3.01) | 0.13 |  | |
| IL-8 | *S. aureus* | 107 | 1.08 (0.65 to 1.80) | 0.77 | |  | IL-8 | | *S. aureus* | 35 | 0.96 (0.38 to 2.39) | 0.92 |  | IL-8 | | *S. aureus* | 72 | 1.32 (0.69 to 2.54) | 0.40 |  | |
| IL-9 | *S. aureus* | 115 | 1.23 (0.83 to 1.81) | 0.30 | |  | IL-9 | | *S. aureus* | 36 | 1.62 (0.61 to 4.29) | 0.32 |  | IL-9 | | *S. aureus* | 79 | 1.14 (0.76 to 1.7) | 0.53 |  | |
| IL-10 | *S. aureus* | 115 | 1.20 (0.74 to 1.94) | 0.46 | |  | IL-10 | | *S. aureus* | 36 | 1.09 (0.55 to 2.19) | 0.79 |  | IL-10 | | *S. aureus* | 79 | 1.33 (0.7 to 2.53) | 0.39 |  | |
| IL-12p70 | *S. aureus* | 115 | 1.13 (0.53 to 2.43) | 0.75 | |  | IL-12p70 | | *S. aureus* | 36 | 1.25 (0.24 to 6.46) | 0.78 |  | IL-12p70 | | *S. aureus* | 79 | 1.08 (0.43 to 2.74) | 0.86 |  | |
| IL-12p40 | *S. aureus* | 115 | 1.22 (0.77 to 1.92) | 0.39 | |  | IL-12p40 | | *S. aureus* | 36 | 1.42 (0.52 to 3.90) | 0.48 |  | IL-12p40 | | *S. aureus* | 79 | 1.2 (0.7 to 2.04) | 0.50 |  | |
| IL-13 | *S. aureus* | 115 | 1.53 (0.73 to 3.18) | 0.25 | |  | IL-13 | | *S. aureus* | 36 | 1.77 (0.35 to 9.02) | 0.48 |  | IL-13 | | *S. aureus* | 79 | 1.45 (0.61 to 3.46) | 0.40 |  | |
| IL-15 | *S. aureus* | 115 | 0.84 (0.47 to 1.51) | 0.55 | |  | IL-15 | | *S. aureus* | 36 | 0.34 (0.10 to 1.13) | 0.076 |  | IL-15 | | *S. aureus* | 79 | 1.15 (0.59 to 2.23) | 0.68 |  | |
| IL-16 | *S. aureus* | 115 | 1.01 (0.76 to 1.36) | 0.92 | |  | IL-16 | | *S. aureus* | 36 | 0.90 (0.65 to 1.24) | 0.51 |  | IL-16 | | *S. aureus* | 79 | 1.13 (0.75 to 1.71) | 0.54 |  | |
| IL-17 | *S. aureus* | 115 | 1.01 (0.80 to 1.29) | 0.91 | |  | IL-17 | | *S. aureus* | 36 | 0.84 (0.55 to 1.31) | 0.43 |  | IL-17 | | *S. aureus* | 79 | 1.12 (0.82 to 1.52) | 0.48 |  | |
| IL-18 | *S. aureus* | 115 | 1.21 (0.75 to 1.97) | 0.43 | |  | IL-18 | | *S. aureus* | 36 | 0.66 (0.46 to 0.94) | 0.022 |  | IL-18 | | *S. aureus* | 79 | 1.49 (0.75 to 2.94) | 0.25 |  | |
| IP-10 | *S. aureus* | 115 | 1.36 (0.91 to 2.04) | 0.14 | |  | IP-10 | | *S. aureus* | 36 | 1.08 (0.58 to 2.03) | 0.80 |  | IP-10 | | *S. aureus* | 79 | 1.43 (0.84 to 2.45) | 0.18 |  | |
| LIF | *S. aureus* | 115 | 1.10 (0.89 to 1.37) | 0.38 | |  | LIF | | *S. aureus* | 36 | 0.90 (0.63 to 1.30) | 0.58 |  | LIF | | *S. aureus* | 79 | 1.16 (0.88 to 1.52) | 0.28 |  | |
| MCP-1 | *S. aureus* | 70 | 1.20 (0.63 to 2.27) | 0.57 | |  | MCP-1 | | *S. aureus* | 22 | 1.42 (0.48 to 4.21) | 0.50 |  | MCP-1 | | *S. aureus* | 48 | 1.14 (0.47 to 2.76) | 0.76 |  | |
| MCP-3 | *S. aureus* | 115 | 1.01 (0.72 to 1.41) | 0.97 | |  | MCP-3 | | *S. aureus* | 36 | 1.12 (0.71 to 1.78) | 0.62 |  | MCP-3 | | *S. aureus* | 79 | 0.96 (0.61 to 1.52) | 0.87 |  | |
| M-CSF | *S. aureus* | 115 | 0.99 (0.75 to 1.31) | 0.95 | |  | M-CSF | | *S. aureus* | 36 | 0.78 (0.53 to 1.16) | 0.22 |  | M-CSF | | *S. aureus* | 79 | 1.03 (0.74 to 1.44) | 0.87 |  | |
| MIF | *S. aureus* | 115 | 1.07 (0.91 to 1.25) | 0.44 | |  | MIF | | *S. aureus* | 36 | 1.07 (0.78 to 1.45) | 0.67 |  | MIF | | *S. aureus* | 79 | 1.09 (0.89 to 1.33) | 0.40 |  | |
| MIG | *S. aureus* | 115 | 1.07 (0.76 to 1.51) | 0.68 | |  | MIG | | *S. aureus* | 36 | 1.10 (0.60 to 2.00) | 0.75 |  | MIG | | *S. aureus* | 79 | 1.07 (0.69 to 1.66) | 0.76 |  | |
| MIP-1𝛼 | *S. aureus* | 110 | 1.46 (0.85 to 2.51) | 0.17 | |  | MIP-1𝛼 | | *S. aureus* | 34 | 1.81 (0.74 to 4.41) | 0.19 |  | MIP-1𝛼 | | *S. aureus* | 76 | 1.37 (0.67 to 2.79) | 0.38 |  | |
| MIP-1𝛽 | *S. aureus* | 112 | 1.30 (0.95 to 1.78) | 0.097 | |  | MIP-1𝛽 | | *S. aureus* | 35 | 1.25 (0.69 to 2.26) | 0.45 |  | MIP-1𝛽 | | *S. aureus* | 77 | 1.37 (0.94 to 2.01) | 0.10 |  | |
| 𝛽-NGF | *S. aureus* | 115 | 1.24 (0.69 to 2.23) | 0.48 | |  | 𝛽-NGF | | *S. aureus* | 36 | 0.78 (0.25 to 2.39) | 0.65 |  | 𝛽-NGF | | *S. aureus* | 79 | 1.46 (0.71 to 3.01) | 0.30 |  | |
| PDGF-BB | *S. aureus* | 115 | 1.08 (0.79 to 1.48) | 0.63 | |  | PDGF-BB | | *S. aureus* | 36 | 0.99 (0.73 to 1.34) | 0.93 |  | PDGF-BB | | *S. aureus* | 79 | 1.21 (0.78 to 1.87) | 0.39 |  | |
| RANTES | *S. aureus* | 115 | 1.16 (0.92 to 1.46) | 0.21 | |  | RANTES | | *S. aureus* | 36 | 1.03 (0.67 to 1.58) | 0.88 |  | RANTES | | *S. aureus* | 79 | 1.24 (0.93 to 1.65) | 0.15 |  | |
| SCF | *S. aureus* | 115 | 1.06 (0.81 to 1.39) | 0.65 | |  | SCF | | *S. aureus* | 36 | 1.04 (0.83 to 1.29) | 0.75 |  | SCF | | *S. aureus* | 79 | 1.1 (0.75 to 1.62) | 0.62 |  | |
| SCGF-𝛽 | *S. aureus* | 115 | 1.05 (0.78 to 1.41) | 0.75 | |  | SCGF-𝛽 | | *S. aureus* | 36 | 1.02 (0.87 to 1.19) | 0.83 |  | SCGF-𝛽 | | *S. aureus* | 79 | 1.08 (0.69 to 1.67) | 0.74 |  | |
| SDF-1𝛼 | *S. aureus* | 115 | 1.07 (0.80 to 1.43) | 0.64 | |  | SDF-1𝛼 | | *S. aureus* | 36 | 1.02 (0.90 to 1.17) | 0.71 |  | SDF-1𝛼 | | *S. aureus* | 79 | 1.11 (0.72 to 1.71) | 0.63 |  | |
| TNF-𝛼 | *S. aureus* | 112 | 1.14 (0.76 to 1.74) | 0.58 | |  | TNF-𝛼 | | *S. aureus* | 36 | 0.84 (0.42 to 1.67) | 0.61 |  | TNF-𝛼 | | *S. aureus* | 79 | 1.61 (0.82 to 3.16) | 0.16 |  | |
| TNF-𝛽 | *S. aureus* | 112 | 1.14 (0.76 to 1.75) | 0.58 | |  | TNF-𝛽 | | *S. aureus* | 36 | 1.22 (0.89 to 1.66) | 0.20 |  | TNF-𝛽 | | *S. aureus* | 79 | 1.17 (0.87 to 1.57) | 0.29 |  | |
| TRAIL | *S. aureus* | 112 | 1.14 (0.76 to 1.75) | 0.59 | |  | TRAIL | | *S. aureus* | 36 | 0.95 (0.63 to 1.43) | 0.79 |  | TRAIL | | *S. aureus* | 79 | 1.25 (0.91 to 1.73) | 0.17 |  | |
| VEGF | *S. aureus* | 112 | 1.14 (0.76 to 1.75) | 0.59 | |  | VEGF | | *S. aureus* | 36 | 1.61 (0.40 to 6.56) | 0.49 |  | VEGF | | *S. aureus* | 79 | 1.61 (0.82 to 3.18) | 0.17 |  | |

GMR geometric mean ratio; 95% CI, 95% confidence interval

| **Supplementary Table 7b:** Cytokine respones 28 days after 2 doses of BNT162b2 compared to ChAdOx1-S vaccination | | | | |  | | **Supplementary Table 8b:** Cytokine respones 28 days after 2 doses of BNT162b2 compared to ChAdOx1-S vaccination in the subgroup of particiants who did not receive BCG vaccination in the BRACE trial | | | | |  | **Supplementary Table 9b:** Cytokine respones 28 days after 2 doses of BNT162b2 compared to ChAdOx1-S vaccination in the subgroup of particiants who did receive BCG vaccination in the BRACE trial | | | | | | |
| --- | --- | --- | --- | --- | --- | --- | --- | --- | --- | --- | --- | --- | --- | --- | --- | --- | --- | --- | --- |
| Cytokine | Stimulant | n | DoM (95% CI) | p-value | |  | Cytokine | Stimulant | n | DoM (95% CI) | p-value |  | Cytokine | Stimulant | n | DoM (95% CI) | p-value |  |  |
| IL-3 | iSARS | 110 | -1.51 (-3.89 to 0.87) | 0.21 | |  | IL-3 | iSARS | 33 | 0.76 (-4.7 to 6.22) | 0.77 |  | IL-3 | iSARS | 77 | -1.7 (-4.12 to 0.72) | 0.17 |  |  |
| IL-7 | iSARS | 110 | 0 (-0.96 to 0.96) | 1.0 | |  | IL-7 | iSARS | 33 | 0 (-24.72 to 24.72) | 1.0 |  | IL-7 | iSARS | 77 | 0 (-3.8 to 3.8) | 1.0 |  |  |
| IL-3 | BCG | 115 | -0.66 (-5.13 to 3.81) | 0.77 | |  | IL-3 | BCG | 36 | -0.35 (-18.15 to 17.44) | 0.97 |  | IL-3 | BCG | 79 | -3.23 (-8.51 to 2.05) | 0.23 |  |  |
| IL-7 | BCG | 115 | 5.75 (-11.62 to 23.11) | 0.51 | |  | IL-7 | BCG | 36 | 11.8 (-11.08 to 34.67) | 0.30 |  | IL-7 | BCG | 79 | -0.83 (-15.51 to 13.85) | 0.91 |  |  |
| IL-3 | *C. albicans* | 115 | -1.92 (-4.29 to 0.46) | 0.11 | |  | IL-3 | *C. albicans* | 36 | 2.54 (-4.41 to 9.49) | 0.46 |  | IL-3 | *C. albicans* | 79 | -2.07 (-5.14 to 1) | 0.18 |  |  |
| IL-7 | *C. albicans* | 115 | 0 (-2.21 to 2.21) | 1.0 | |  | IL-7 | *C. albicans* | 36 | 0.47 (-21.72 to 22.66) | 0.97 |  | IL-7 | *C. albicans* | 79 | 0 (-2.67 to 2.67) | 1.0 |  |  |
| IL-3 | *E. coli* | 115 | 1.85 (-1.77 to 5.47) | 0.31 | |  | IL-3 | *E. coli* | 36 | -0.3 (-11.6 to 10.99) | 0.96 |  | IL-3 | *E. coli* | 79 | 1.62 (-2.84 to 6.08) | 0.47 |  |  |
| IL-7 | *E. coli* | 115 | 7.13 (-4.71 to 18.96) | 0.24 | |  | IL-7 | *E. coli* | 36 | 5.84 (-34.13 to 45.82) | 0.77 |  | IL-7 | *E. coli* | 79 | -0.82 (-13.01 to 11.37) | 0.89 |  |  |
| IL-3 | R848 | 115 | -0.61 (-4.12 to 2.9) | 0.73 | |  | IL-3 | R848 | 36 | 2.03 (-12.45 to 16.51) | 0.78 |  | IL-3 | R848 | 79 | -0.99 (-6.52 to 4.54) | 0.72 |  |  |
| IL-7 | R848 | 115 | 5.22 (-11.1 to 21.54) | 0.53 | |  | IL-7 | R848 | 36 | 2.59 (-37.8 to 42.98) | 0.90 |  | IL-7 | R848 | 79 | -1.9 (-25.37 to 21.57) | 0.87 |  |  |
| IL-3 | *S. aureus* | 115 | -1.4 (-4.5 to 1.71) | 0.37 | |  | IL-3 | *S. aureus* | 36 | -2.82 (-10.77 to 5.13) | 0.47 |  | IL-3 | *S. aureus* | 79 | -1.24 (-4.94 to 2.46) | 0.51 |  |  |
| IL-7 | *S. aureus* | 115 | 0 (-2.11 to 2.11) | 1.0 | |  | IL-7 | *S. aureus* | 36 | 0 (-31.87 to 31.87) | 1.0 |  | IL-7 | *S. aureus* | 79 | 0 (-3.9 to 3.9) | 1.0 |  |  |

DoM difference in Medians; 95% CI, 95% confidence interval

| **Supplementary Table 7c**: Sensitivity analysis of Cytokine response 28 days after 2 doses of BNT162b2 compared to ChAdOx1-S vaccination with values above ULD replaced as double highest value | | | | |
| --- | --- | --- | --- | --- |
| Cytokine | Stimulant | n | GMR (95% CI) | p-value |
| IL-6 | iSARS | 110 | 1.11 (0.8 to 1.54) | 0.53 |
| IL-8 | iSARS | 110 | 0.87 (0.68 to 1.11) | 0.25 |
| MCP-1 | iSARS | 110 | 1.2 (0.45 to 3.2) | 0.71 |
| MIP-1𝛼 | iSARS | 110 | 0.88 (0.64 to 1.21) | 0.41 |
| MIP-1𝛽 | iSARS | 110 | 0.94 (0.72 to 1.22) | 0.62 |
| IL-6 | BCG | 115 | 1.35 (0.66 to 2.79) | 0.41 |
| IL-8 | BCG | 115 | 1.42 (0.36 to 5.62) | 0.62 |
| MCP-1 | BCG | 115 | 0.99 (0.34 to 2.89) | 0.99 |
| MIP-1𝛼 | BCG | 115 | 1.11 (0.53 to 2.31) | 0.79 |
| MIP-1𝛽 | BCG | 115 | 0.61 (0.13 to 2.79) | 0.52 |
| IL-6 | *C. albicans* | 115 | 0.77 (0.33 to 1.81) | 0.54 |
| IL-8 | *C. albicans* | 115 | 0.5 (0.19 to 1.31) | 0.16 |
| MCP-1 | *C. albicans* | 115 | 0.73 (0.23 to 2.3) | 0.59 |
| MIP-1𝛼 | *C. albicans* | 115 | 0.62 (0.24 to 1.56) | 0.30 |
| MIP-1𝛽 | *C. albicans* | 115 | 0.72 (0.43 to 1.22) | 0.22 |
| IL-6 | *E. coli* | 115 | 1.26 (0.56 to 2.83) | 0.57 |
| IL-8 | *E. coli* | 115 | 1.12 (0.61 to 2.05) | 0.71 |
| MCP-1 | *E. coli* | 115 | 0.64 (0.33 to 1.24) | 0.18 |
| MIP-1𝛼 | *E. coli* | 115 | 1.28 (0.62 to 2.65) | 0.50 |
| MIP-1𝛽 | *E. coli* | 115 | 0.98 (0.28 to 3.45) | 0.98 |
| IL-6 | R848 | 115 | 1.75 (0.79 to 3.85) | 0.16 |
| IL-8 | R848 | 115 | 1.08 (0.8 to 1.45) | 0.62 |
| MCP-1 | R848 | 115 | 0.96 (0.49 to 1.89) | 0.91 |
| MIP-1𝛼 | R848 | 115 | 1.27 (0.59 to 2.71) | 0.54 |
| MIP-1𝛽 | R848 | 115 | 1.31 (0.35 to 4.9) | 0.69 |
| IL-6 | *S. aureus* | 115 | 1.06 (0.58 to 1.92) | 0.85 |
| IL-8 | *S. aureus* | 115 | 0.69 (0.26 to 1.8) | 0.44 |
| MCP-1 | *S. aureus* | 115 | 2.09 (0.66 to 6.6) | 0.20 |
| MIP-1𝛼 | *S. aureus* | 115 | 1.35 (0.66 to 2.76) | 0.41 |
| MIP-1𝛽 | *S. aureus* | 115 | 1.09 (0.56 to 2.11) | 0.80 |
