# Supplementary figures and images for: Specific and off-target immune responses following COVID-19 vaccination with ChAdOx1-S and BNT162b2 vaccines—an exploratory sub-study of the BRACE trial
Source: eBioMedicine. 2024 Apr 24;103:105100. doi: 10.1016/j.ebiom.2024.105100 (PMC11058726; doi:10.1016/j.ebiom.2024.105100)

Supplementary Figure 1  
a

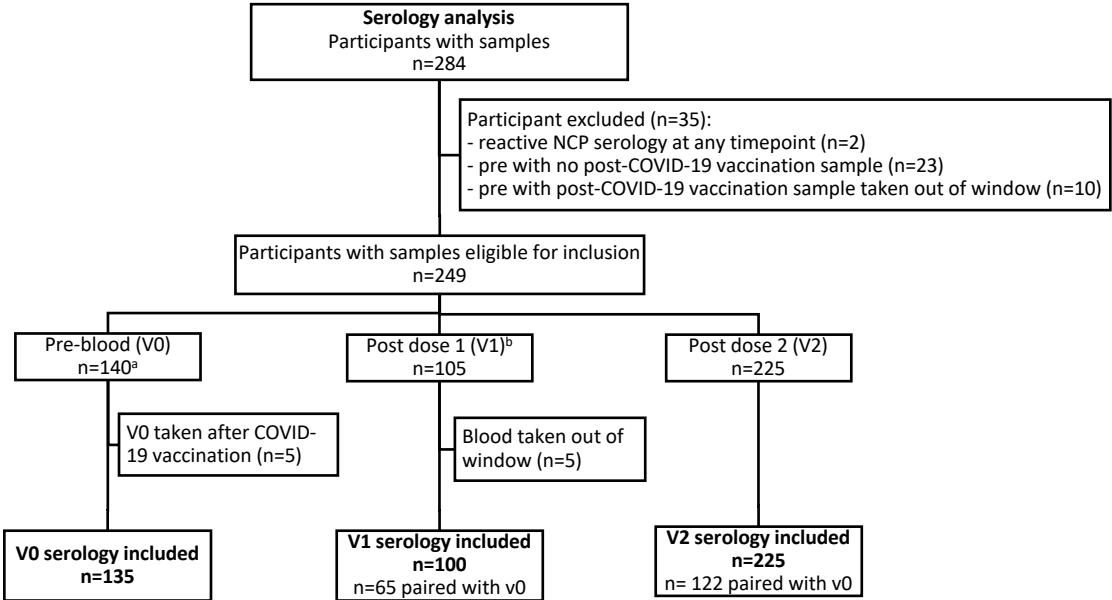

b

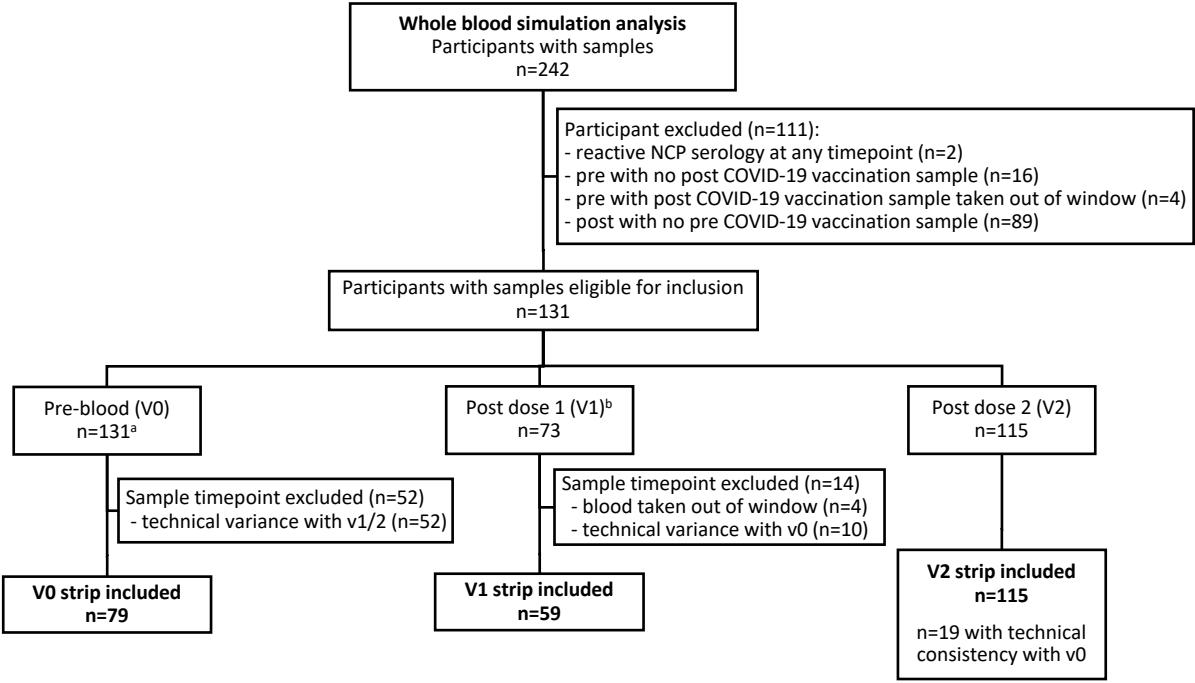

Supplement: Supplementary Fig. S1 — Participant study flow diagrams. Diagram of participants and samples included for (a) serology and (b) whole blood stimulation analysis. aexcludes v1 samples collected under alternative protocol in South Australia. [file mmc3.pdf]

## Supplementary Figure 2

a

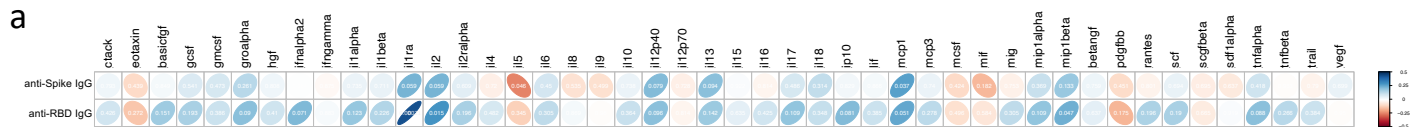

b

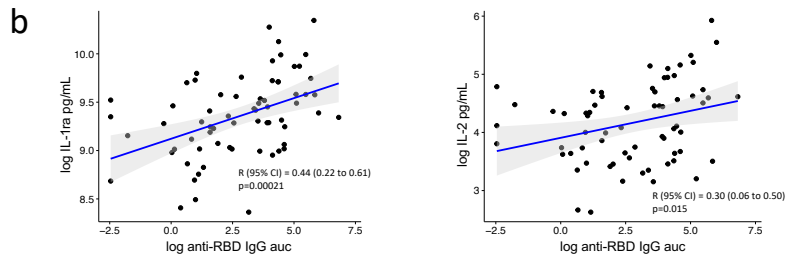

C

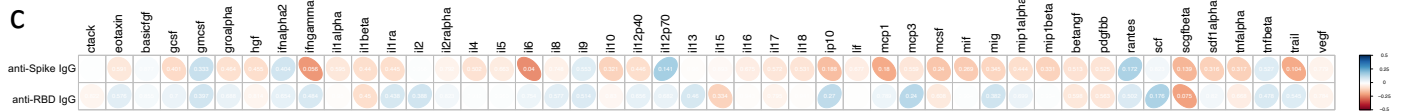

d

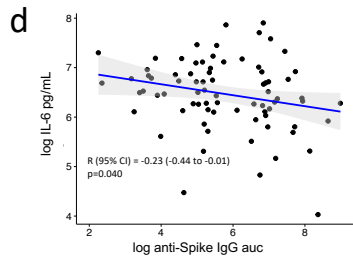

e

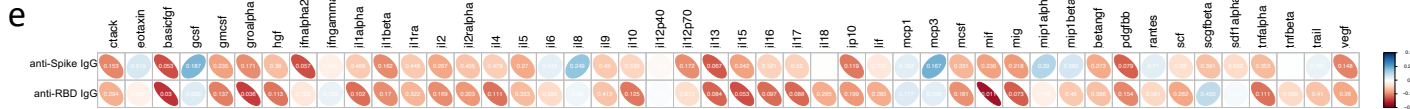

**f**

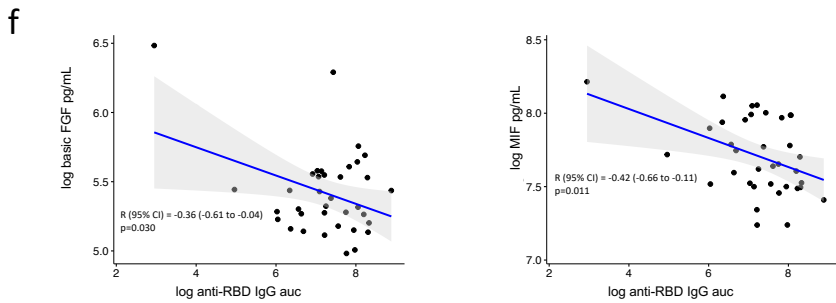

Supplement: Supplementary Fig. S2 — Correlations between IgG and iSARS responses following ChAdOx1-S and BNT162b2 vaccination. (a, c, e) Heatmaps of Pearson correlation’s co-efficients and (b, d, f) representative scatter plots showing correlation between serum IgG response and iSARS stimulation induced cytokines 28 (±3 days) after (a and b) one (V1, n = 69) and (c and d) two doses (V2, n = 78) of ChAdOx1-S and (e and f) two doses (V2, n = 78) of BNT162b2 vaccination. Data were log transformed prior to Pearson’s correlation, o-values for Pearson’s correlation are presented. Heatmaps: Red indicates a negative correlation, whereas blue indicates a positive correlation. Scatter plots: blue line depicts regression line, grey indicates 95% confidence interval (CI). [file mmc4.pdf]

# Supplementary Figure 3

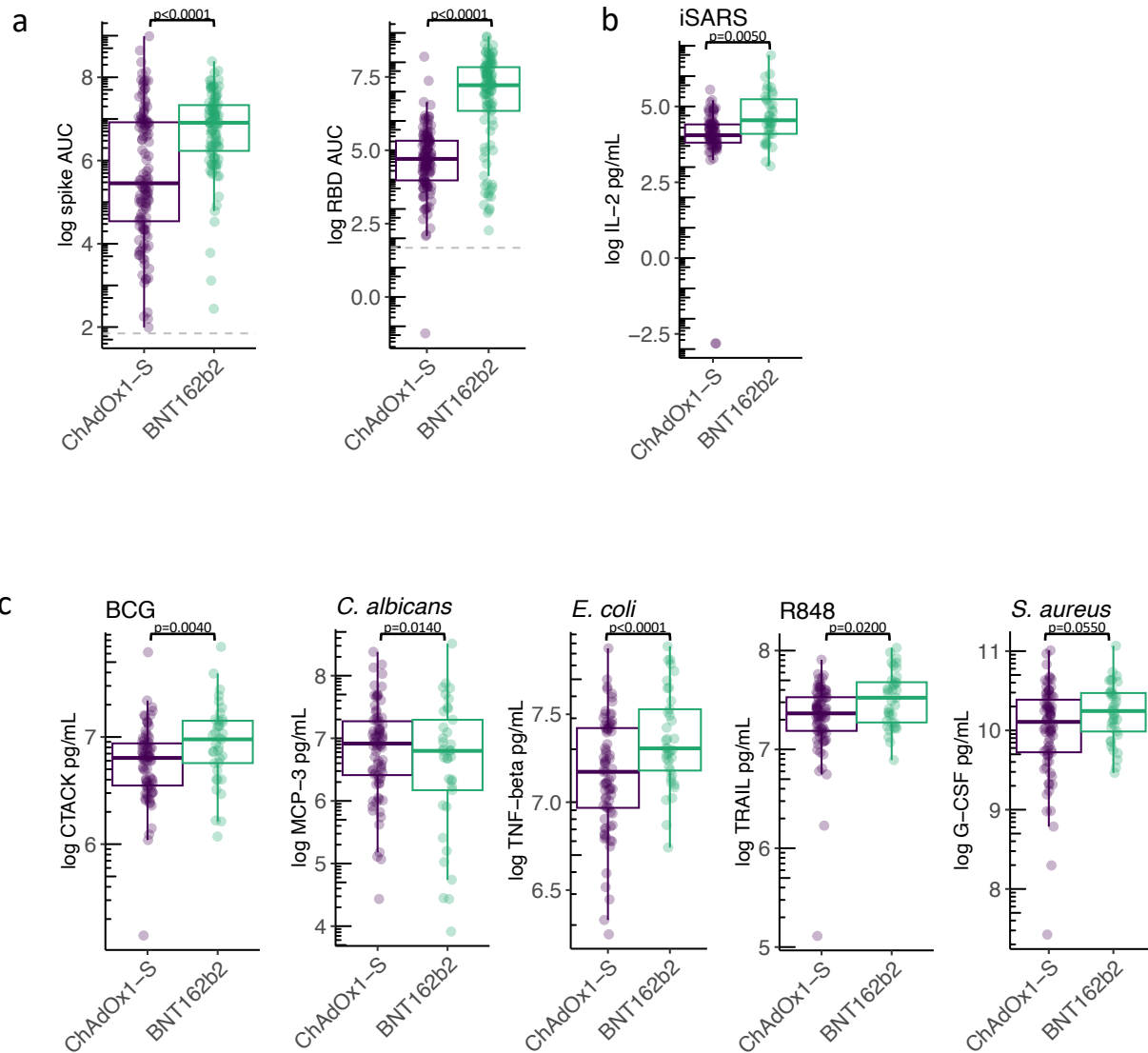

Supplement: Supplementary Fig. S3 — Specific and off-target in vitro immune responses 28 days after the second dose of BNT162b2 compared to ChAdOx1-S. (a) Tukey boxplots and dot plot overlay depicting unadjusted log transformed anti-Spike and anti-RBD IgG AUC (n = 225). Grey dotted line represents the assay cut-off defined based on pre-2019 serum samples from healthy adults. (b and c) Tukey boxplots (with dot plot overlay) depicting unadjusted log transformed cytokine pg/mL secreted in response to stimulation of whole blood with (b) γ-irradiated SARS-CoV-2 (iSARS) and (c) BCG, C. albicans, E. coli, S. aureus and R848. Differences between vaccines determined by linear regression of log-transformed antibody and cytokine responses (adjusted only for control i.e., nil or iVero). [file mmc5.pdf]
