# Supplementary material for: Specific and off-target immune responses following COVID-19 vaccination with ChAdOx1-S and BNT162b2 vaccines—an exploratory sub-study of the BRACE trial
Source: eBioMedicine. 2024 Apr 24;103:105100. doi: 10.1016/j.ebiom.2024.105100 (PMC11058726; doi:10.1016/j.ebiom.2024.105100)
Supplement: BRACE trial Consortium Group [file mmc6.docx]

**BRACE Trial consortium group**

| **First Names + Middle Names** | **Last Names** |
| --- | --- |
| Nigel | Curtis |
| Andrew | Davidson |
| Kaya | Gardiner |
| Amanda | Gwee |
| Tenaya | Jamieson |
| Nicole | Messina |
| Thilanka | Morawakage |
| Susan | Perlen |
| Kirsten | Perrett |
| Laure | Pittet |
| Amber | Sastry |
| Jia Wei | Teo |
| Francesca | Orsini |
| Katherine | Lee |
| Cecilia | Moore |
| Suzanna | Vidmar |
| Laure | Pittet |
| Rashida | Ali |
| Ross | Dunn |
| Peta | Edler |
| Grace | Gell |
| Casey | Goodall |
| Richard | Hall |
| Ann | Krastev |
| Nathan | La |
| Ellie | McDonald |
| Nick | McPhate |
| Thao | Nguyen |
| Jack | Ren |
| Luke | Stevens |
| Nicole | Messina |
| Ahmed | Alamrousi |
| Rhian | Bonnici |
| Thanh | Dang |
| Susie | Germano |
| Jenny | Hua |
| Rebecca | McElroy |
| Monica | Razmovska |
| Scott | Reddiex |
| Xiaofang | Wang |
| Jeremy | Anderson |
| Kristy | Azzopardi |
| Vicki | Bennett-Wood |
| Anna | Czajko |
| Nadia | Mazarakis |
| Conor | McCafferty |
| Frances | Oppedisano |
| Belinda | Ortika |
| Casey | Pell |
| Leena | Spry |
| Ryan | Toh |
| Sunitha | Velagapudi |
| Amanda | Vlahos |
| Ashleigh | Wee-Hee |
| Pedro | Ramos |
| Karina | De La Cruz |
| Dinusha | Gamage |
| Anushka | Karunanayake |
| Isabella | Mezzetti |
| Benjamin | Ong |
| Ronita | Singh |
| Enoshini | Sooriyarachchi |
| Suellen | Nicholson |
| Natalie | Cain |
| Rianne | Brizuela |
| Han | Huang |
| Veronica | Abruzzo |
| Morgan | Bealing |
| Patricia | Bimboese |
| Kirsty | Bowes |
| Emma | Burrell |
| Joyce | Chan |
| Jac | Cushnahan |
| Hannah | Elborough |
| Olivia | Elkington |
| Kieran | Fahey |
| Monique | Fernandez |
| Catherine | Flynn |
| Sarah | Fowler |
| Marie | Gentile Andrit |
| Bojana | Gladanac |
| Catherine | Hammond |
| Norine | Ma |
| Sam | Macalister |
| Emmah | Milojevic |
| Jesutofunmi | Mojeed |
| Jill | Nguyen |
| Liz | O'Donnell |
| Nadia | Olivier |
| Isabelle | Ooi |
| Stephanie | Reynolds |
| Lisa | Shen |
| Barb | Sherry |
| Judith | Spotswood |
| Jamie | Wedderburn |
| Angela | Younes |
| Donna | Legge |
| Jason | Bell |
| Jo | Cheah |
| Annie | Cobbledick |
| Kee | Lim |
| Sonja | Elia |
| Lynne | Addlem |
| Anna | Bourke |
| Clare | Brophy |
| Nadine | Henare |
| Narelle | Jenkins |
| Francesca | Machingaifa |
| Skye | Miller |
| Kirsten | Mitchell |
| Sigrid | Pitkin |
| Kate | Wall |
| Paola | Villanueva |
| Nigel | Crawford |
| Laure | Pittet |
| Wendy | Norton |
| Niki | Tan |
| Thilakavathi | Chengodu |
| Diane | Dawson |
| Victoria | Gordon |
| Tony | Korman |
| Jess | O'Bryan |
| Veronica | Abruzzo |
| Sophie | Agius |
| Samantha | Bannister |
| Jess | Bucholc |
| Alison | Burns |
| Beatriz | Camesella |
| John | Carlin |
| Marianna | Ciaverella |
| Maxwell | Curtis |
| Stephanie | Firth |
| Christina | Guo |
| Matthew | Hannan |
| Erin | Hill |
| Sri | Joshi |
| Katherine | Lieschke |
| Megan | Mathers |
| Sasha | Odoi |
| Ashleigh | Rak |
| Chris | Richards |
| Leah | Steve |
| Carolyn | Stewart |
| Eva | Sudbury |
| Helen | Thomson |
| Emma | Watts |
| Fiona | Williams |
| Angela | Young |
| Penny | Glenn |
| Andrew | Kaynes |
| Amandine | Philippart De Floy |
| Sandy | Buchanan |
| Thijs | Sondag |
| Ivy | Xie |
| Harriet | Edmund |
| Bridie | Byrne |
| Tom | Keeble |
| Belle | Ngien |
| Fran | Noonan |
| Michelle | Wearing-Smith |
| Alison | Clarke |
| Pemma | Davies |
| Oliver | Eastwood |
| Alric | Ellinghaus |
| Rachid | Ghieh |
| Zahra | Hilton |
| Emma | Jennings |
| Athina | Kakkos |
| Iris | Liang |
| Katie | Nicol |
| Sally | O'Callaghan |
| Helen | Osman |
| Gowri | Rajaram |
| Sophia | Ratcliffe |
| Victoria | Rayner |
| Ashleigh | Salmon |
| Angela | Scheppokat |
| Aimee | Stevens |
| Rebekah | Street |
| Nicholas | Toogood |
| Nicholas | Wood |
| Twinkle | Bahaduri |
| Therese | Baulman |
| Jennifer | Byrne |
| Candace | Carter |
| Mary | Corbett |
| Aiken | Dao |
| Maria | Desylva |
| Andrew | Dunn |
| Evangeline | Gardiner |
| Rosemary | Joyce |
| Rama | Kandasamy |
| Craig | Munns |
| Lisa | Pelayo |
| Ketaki | Sharma |
| Katrina | Sterling |
| Caitlin | Uren |
| Clinton | Colaco |
| Mark | Douglas |
| Kate | Hamilton |
| Adam | Bartlett |
| Brendan | McMullan |
| Pamela | Palasanthiran |
| Phoebe | Williams |
| Justin | Beardsley |
| Nikki | Bergant |
| Renier | Lagunday |
| Kristen | Overton |
| Jeffrey | Post |
| Yasmeen | Al-Hindawi |
| Sarah | Barney |
| Anthony | Byrne |
| Lee | Mead |
| Marshall | Plit |
| David | Lynn |
| Saoirse | Benson |
| Stephen | Blake |
| Rochelle | Botten |
| Tee Yee | Chern |
| Georgina | Eden |
| Liddy | Griffith |
| Jane | James |
| Miriam | Lynn |
| Angela | Markow |
| Domenic | Sacca |
| Natalie | Stevens |
| Steve | Wesselingh |
| Catriona | Doran |
| Simone | Barry |
| Alice | Sawka |
| Sue | Evans |
| Louise | Goodchild |
| Christine | Heath |
| Meredith | Krieg |
| Helen | Marshall |
| Mark | McMillan |
| Mary | Walker |
| Peter | Richmond |
| Nelly | Amenyogbe |
| Christina | Anthony |
| Annabelle | Arnold |
| Beth | Arrowsmith |
| Rym | Ben-Othman |
| Sharon | Clark |
| Jemma | Dunnill |
| Nat | Eiffler |
| Krist | Ewe |
| Carolyn | Finucane |
| Lorraine | Flynn |
| Camille | Gibson |
| Lucy | Hartnell |
| Elysia | Hollams |
| Heidi | Hutton |
| Lance | Jarvis |
| Jane | Jones |
| Jan | Jones |
| Karen | Jones |
| Jennifer | Kent |
| Tobias | Kollmann |
| Debbie | Lalich |
| Wenna | Lee |
| Rachel | Lim |
| Sonia | McAlister |
| Fiona | McDonald |
| Andrea | Meehan |
| Asma | Minhaj |
| Lisa | Montgomery |
| Melissa | O’Donnell |
| Jaslyn | Ong |
| Joanne | Ong |
| Kimberley | Parkin |
| Glady | Perez |
| Catherine | Power |
| Shadie | Rezazadeh |
| Holly | Richmond |
| Sally | Rogers |
| Nikki | Schultz |
| Margaret | Shave |
| Patrycja | Skut |
| Lisa | Stiglmayer |
| Alexandra | Truelove |
| Ushma | Wadia |
| Rachael | Wallace |
| Justin | Waring |
| Michelle | England |
| Erin | Latkovic |
| Laurens | Manning |
| Susan | Herrmann |
| Michaela | Lucas |
| Marcus | Lacerda |
| Paulo Henrique | Andrade |
| Fabiane Bianca | Barbosa |
| Dayanne | Barros |
| Larissa | Brasil |
| Ana Greyce | Capella |
| Ramon | Castro |
| Erlane | Costa |
| Dilcimar | de Souza |
| Maianne | Dias |
| José | Dias |
| Klenilson | Ferreira |
| Paula | Figueiredo |
| Thamires | Freitas |
| Ana Carolina | Furtado |
| Larissa | Gama |
| Vanessa | Godinho |
| Cintia | Gouy |
| Daniele | Hinojosa |
| Bruno | Jardim |
| Tyane | Jardim |
| Joel | Junior |
| Augustto | Lima |
| Bernardo | Maia |
| Adriana | Marins |
| Kelry | Mazurega |
| Tercilene | Medeiros |
| Rosangela | Melo |
| Marinete | Moraes |
| Elizandra | Nascimento |
| Juliana | Neves |
| Maria Gabriela | Oliveira |
| Thais | Oliveira |
| Ingrid | Oliveira |
| Arthur | Otsuka |
| Rayssa | Paes |
| Handerson | Pereira |
| Gabrielle | Pereira |
| Christiane | Prado |
| Evelyn | Queiroz |
| Laleyska | Rodrigues |
| Bebeto | Rodrigues |
| Vanderson | Sampaio |
| Anna Gabriela | Santos |
| Daniel | Santos |
| Tilza | Santos |
| Evelyn | Santos |
| Ariandra | Sartim |
| Ana Beatriz | Silva |
| Juliana | Silva |
| Emanuelle | Silva |
| Mariana | Simão |
| Caroline | Soares |
| Antonny | Sousa |
| Alexandre | Trindade |
| Fernando | Val |
| Adria | Vasconcelos |
| Heline | Vasconcelos |
| Julio | Croda |
| Carolinne | Abreu |
| Katya Martinez | Almeida |
| Camila Bitencourt de | Andrade |
| Jhenyfer Thalyta Campos | Angelo |
| Ghislaine Gonçalvez de Araújo | Arcanjo |
| Bianca Maria Silva Menezes | Arruda |
| Wellyngthon Espindola | Ayala |
| Adelita Agripina Refosco | Barbosa |
| Felipe Zampieri Vieira | Batista |
| Fabiani de Morais | Batista |
| Miriam de Jesus | Costa |
| Mariana Garcia | Croda |
| Lais Alves da | Cruz |
| Roberta Carolina Pereira | Diogo |
| Rodrigo Cezar Dutra | Escobar |
| Iara Rodrigues | Fernandes |
| Leticia Ramires | Figueiredo |
| Leandro Galdino Cavalcanti | Gonçalves |
| Sarita | Lahdo |
| Joyce dos Santos | Lencina |
| Guilherme Teodoro de | Lima |
| Larissa Santos | Matos |
| Bruna Tayara Leopoldina | Meireles |
| Debora Quadros | Moreira |
| Lilian Batista Silva | Muranaka |
| Adriely de | Oliveira |
| Karla Regina Warszawski de | Oliveira |
| Matheus Vieira de | Oliveira |
| Roberto Dias de | Oliveira |
| Andrea Antonia Souza de Almeida dos Reis | Pereira |
| Marco | Puga |
| Caroliny Veron | Ramos |
| Thaynara Haynara Souza da | Rosa |
| Karla Lopes dos | Santos |
| Claudinalva Ribeiro dos | Santos |
| Dyenyffer Stéffany Leopoldina dos | Santos |
| Karina Marques | Santos |
| Paulo César Pereira da | Silva |
| Paulo Victor Rocha da | Silva |
| Débora dos Santos | Silva |
| Patricia Vieira da | Silva |
| Bruno Freitas da Rosa | Soares |
| Mariana Gazzoni | Sperotto |
| Mariana Mayumi | Tadokoro |
| Daniel | Tsuha |
| Hugo Miguel Ramos | Vieira |
| Margareth Maria Pretti | Dalcolmo |
| Cíntia Maria Lopes | Alves da Paixão |
| Gabriela Corrêa E | Castro |
| Simone Silva | Collopy |
| Renato | da Costa Silva |
| Samyra Almeida | da Silveira |
| Alda Maria | Da-Cruz |
| Alessandra Maria da Silva Passos | de Carvalho |
| Rita | de Cássia Batista |
| Maria Luciana Silva | De Freitas |
| Aline Gerhardt | de Oliveira Ferreira |
| Ana Paula Conceição | de Souza |
| Paola Cerbino | Doblas |
| Ayla Alcoforado da Silva | dos Santos |
| Vanessa Cristine de Moraes | dos Santos |
| Dayane Alves | dos Santos Gomes |
| Anderson Lage | Fortunato |
| Adriano | Gomes-Silva |
| Monique Pinto | Gonçalves |
| Paulo Leandro Garcia Meireless | Junior |
| Estela | Martins da Costa Carvalho |
| Fernando do Couto | Motta |
| Ligia Maria | Olivo de Mendonça |
| Girlene dos Santos | Pandine |
| Rosa Maria Plácido | Pereira |
| Ivan | Ramos Maia |
| Jorge Luiz da | Rocha |
| João Victor Paiva | Romano |
| Glauce dos | Santos |
| Erica Fernandes da | Silva |
| Marilda Agudo Mendonça Teixeira de | Siqueira |
| Ágatha Cristinne Prudêncio | Soares |
| Marc | Bonten |
| Sandra Franch | Arroyo |
| Henny Ophorst-den | Besten |
| Anna | Boon |
| Karin M | Brakke |
| Axel | Janssen |
| Marijke A.H. | Koopmans |
| Toos | Lemmens |
| Titia | Leurink |
| Cristina | Prat-Aymerich |
| Engelien | Septer-Bijleveld |
| Kimberly | Stadhouders |
| Darren | Troeman |
| Marije | van der Waal |
| Marjoleine | van Opdorp |
| Nicolette | van Sluis |
| Beatrijs | Wolters |
| Jan | Kluytmans |
| Jannie | Romme |
| Wouter | van den Bijllaardt |
| Linda | van Mook |
| M.M.L (Miranda) | van Rijen |
| P.M.G. | Filius |
| Jet | Gisolf |
| Frances | Greven |
| Danique | Huijbens |
| Robert | Jan Hassing |
| R.C. | Pon |
| Lieke | Preijers |
| J.H. | van Leusen |
| Harald | Verheij |
| Wim | Boersma |
| Evelien | Brans |
| Paul | Kloeg |
| Kitty | Molenaar-Groot |
| Nhat Khanh | Nguyen |
| Nienke | Paternotte |
| Anke | Rol |
| Lida | Stooper |
| Helga | Dijkstra |
| Esther | Eggenhuizen |
| Lucas | Huijs |
| Simone | Moorlag |
| Mihai | Netea |
| Eva | Pranger |
| Esther | Taks |
| Jaap | ten Oever |
| Rob | ter Heine |
| Kitty | Blauwendraat |
| Bob | Meek |
| Isil | Erkaya |
| Houda | Harbech |
| Nienke | Roescher |
| Rifka | Peeters |
| Menno | te Riele |
| Carmen | Zhou |
| Esther | Calbo |
| Cristina Badia | Marti |
| Emma Triviño | Palomares |
| Tomás Perez | Porcuna |
| Anabel | Barriocanal |
| Ana Maria | Barriocanal |
| Irma | Casas |
| Jose | Dominguez |
| Maria | Esteve |
| Alicia | Lacoma |
| Irene | Latorre |
| Gemma | Molina |
| Barbara | Molina |
| Antoni | Rosell |
| Sandra | Vidal |
| Lydia | Barrera |
| Natalia | Bustos |
| Ines Portillo | Calderón |
| David Gutierrez | Campos |
| Jose Manuel | Carretero |
| Angel Dominguez | Castellano |
| Renato | Compagnone |
| Encarnacion Ramirez | de Arellano |
| Almudena | de la Serna |
| Maria Dolores | del Toro Lopez |
| Marie-Alix Clement | Espindola |
| Ana Belen Martin | Gutierrez |
| Alvaro Pascual | Hernandez |
| Virginia Palomo | Jiménez |
| Elisa | Moreno |
| Nicolas | Navarrete |
| Teresa Rodriguez | Paño |
| Jesús | Rodríguez-Baño |
| Enriqueta | Tristán |
| Maria Jose Rios | Villegas |
| Atsegiñe Canga | Garces |
| Erika | Castro Amo |
| Raquel | Coya Guerrero |
| Josune | Goikoetxea |
| Leticia | Jorge |
| Cristina | Perez |
| María Carmen Fariñas | Álvarez |
| Manuel Gutierrez | Cuadra |
| Francisco Arnaiz | de las Revillas Almajano |
| Pilar Bohedo | Garcia |
| Teresa Giménez | Poderos |
| Claudia González | Rico |
| Blanca | Sanchez |
| Olga | Valero |
| Noelia | Vega |
| John | Campbell |
| Anna | Barnes |
| Helen | Catterick |
| Tim | Cranston |
| Phoebe | Dawe |
| Emily | Fletcher |
| Liam | Fouracre |
| Alison | Gifford |
| John | Kirkwood |
| Christopher | Martin |
| Amy | McAnew |
| Marcus | Mitchell |
| Georgina | Newman |
| Abby | O'Connell |
| Jakob | Onysk |
| Lynne | Quinn |
| Shelley | Rhodes |
| Samuel | Stone |
| Lorrie | Symons |
| Harry | Tripp |
| Adilia | Warris |
| Darcy | Watkins |
| Bethany | Whale |
| Alex | Harding |
| Gemma | Lockhart |
| Kate | Sidaway-Lee |
| John | Campbell |
| Sam | Hilton |
| Sarah | Manton |
| Daniel | Webber-Rookes |
| Rachel | Winder |
| James | Moore |
| Freya | Bateman |
| Michael | Gibbons |
| Bridget | Knight |
| Julie | Moss |
| Sarah | Statton |
| Josephine | Studham |
| Lydia | Hall |
| Will | Moyle |
| Tamsin | Venton |
